# Histone H3K27 demethylase KDM6A is an epigenetic gatekeeper of mTORC1 signalling in cancer

**DOI:** 10.1136/gutjnl-2021-325405

**Published:** 2021-09-11

**Authors:** Steffie Revia, Agnieszka Seretny, Lena Wendler, Ana Banito, Christoph Eckert, Kersten Breuer, Anand Mayakonda, Pavlo Lutsik, Matthias Evert, Silvia Ribback, Suchira Gallage, Ismaiel Chikh Bakri, Kai Breuhahn, Peter Schirmacher, Stefan Heinrich, Matthias M Gaida, Mathias Heikenwälder, Diego F Calvisi, Christoph Plass, Scott W Lowe, Darjus F Tschaharganeh

**Affiliations:** 1 Helmholtz-University Group “Cell Plasticity and Epigenetic Remodeling”, German Cancer Research Center (DKFZ) & Institute of Pathology, University Hospital, Heidelberg, Germany; 2 Pediatric Soft Tissue Sarcoma Research Group, German Cancer Research Center (DKFZ), Heidelberg, Germany; 3 Division of Cancer Epigenomics, German Cancer Research Center (DKFZ), Heidelberg, Germany; 4 Institute of Pathology, University of Regensburg, Regensburg, Germany; 5 Institute of Pathology, University Hospital Greifswald, Greifswald, Germany; 6 Division of Chronic Inflammation and Cancer, German Cancer Research Center (DKFZ), Heidelberg, Germany; 7 Institute of Pathology, University Hospital Heidelberg, Heidelberg, Germany; 8 Department of Surgery, University Medical Center Mainz, JGU-Mainz, Mainz, Germany; 9 Institute of Pathology, University Medical Center Mainz, JGU-Mainz, Mainz, Germany; 10 Research Center for Immunotherapy, University Medical Center Mainz, JGU-Mainz, Mainz, Germany; 11 Joint Unit Immunopathology, Institute of Pathology, University Medical Center, JGU-Mainz, Mainz, Germany; 12 TRON, Translational Oncology, University Medical Center, JGU-Mainz, Mainz, Germany; 13 Department of Cancer Biology and Genetics, Sloan Kettering Institute, Memorial Sloan Kettering Cancer Center, New York, NY, USA; 14 Howard Hughes Medical Institute, Chevy Chase, MD, USA

**Keywords:** gastrointestinal cancer, hepatobiliary cancer, hepatocellular carcinoma, molecular carcinogenesis

## Abstract

**Objective:**

Large-scale genome sequencing efforts of human tumours identified epigenetic modifiers as one of the most frequently mutated gene class in human cancer. However, how these mutations drive tumour development and tumour progression are largely unknown. Here, we investigated the function of the histone demethylase KDM6A in gastrointestinal cancers, such as liver cancer and pancreatic cancer.

**Design:**

Genetic alterations as well as expression analyses of KDM6A were performed in patients with liver cancer. Genetic mouse models of liver and pancreatic cancer coupled with Kdm6a-deficiency were investigated, transcriptomic and epigenetic profiling was performed, and in vivo and in vitro drug treatments were conducted.

**Results:**

KDM6A expression was lost in 30% of patients with liver cancer. Kdm6a deletion significantly accelerated tumour development in murine liver and pancreatic cancer models. Kdm6a-deficient tumours showed hyperactivation of mTORC1 signalling, whereas endogenous Kdm6a re-expression by inducible RNA-interference in established Kdm6a-deficient tumours diminished mTORC1 activity resulting in attenuated tumour progression. Genome-wide transcriptional and epigenetic profiling revealed direct binding of Kdm6a to crucial negative regulators of mTORC1, such as Deptor, and subsequent transcriptional activation by epigenetic remodelling. Moreover, in vitro and in vivo genetic epistasis experiments illustrated a crucial function of Deptor and mTORC1 in Kdm6a-dependent tumour suppression. Importantly, KDM6A expression in human tumours correlates with mTORC1 activity and KDM6A-deficient tumours exhibit increased sensitivity to mTORC1 inhibition.

**Conclusion:**

KDM6A is an important tumour suppressor in gastrointestinal cancers and acts as an epigenetic toggle for mTORC1 signalling. Patients with KDM6A-deficient tumours could benefit of targeted therapy focusing on mTORC1 inhibition.

**Significance of this study**

**What is already known on this subject?**

- Large-scale next generation cancer genome sequencing efforts consistently reveal that alterations in genes involved in establishing and interpreting epigenetic landscapes are among the most frequent events in human tumourigenesis. Despite this prevalence, mechanistic insights into how these mutations functionally contribute to cancer development and intersect with other pathways involved in tumourigenesis remain largely unknown.

**What are the new findings?**

- By integrating genomic, genetic and preclinical data we identify the histone demethylase KDM6A as a potent tumour suppressor in liver and pancreatic cancer, provide a mechanistic explanation how KDM6A mediates tumour suppression and a therapeutic strategy how KDM6A-deficient tumours can be treated.

**How might it impact on clinical practice in the foreseeable future?**

- By demonstrating that KDM6A acts as an epigenetic toggle for mTORC1 signalling and that KDM6A deficient tumours respond to mTORC1 inhibitors, we envision to implement KDM6A as a biomarker for mTORC1 centred therapies in gastrointestinal cancers and therefore aid to the emerging field of personalised medicine.

## Introduction

Recent whole-genome sequencing efforts of human tumours catalogued the mutational landscape of virtual every cancer type.^1^ These data revealed many well-known driver genes but also implicated novel genes to be involved in tumourigenesis. In particular, genes encoding chromatin modifiers were found to be altered in many different cancer types,^2–4^ implying their important role in tumour development. However, although some studies could functionally validate their contribution to tumourigenesis,^5 6^ it is still largely unknown for most of these genes how they are mechanistically involved in cancer progression.

The mixed-lineage leukemia protein 3/4 (MLL3/4) complex proteins associated with set1 (COMPASS)-like complex is a multicomponent complex involved in remodelling the epigenetic landscape to facilitate efficient transcriptional activation.^7–9^ This complex contains KMT2C (MLL3) and KMT2D (MLL4), both histone H3K4 methyltransferases, KDM6A (UTX), a H3K27 demethylase, several scaffold proteins (ASH2, WDR5, RBBP5 and hDPY30) also present in other COMPASS complexes, and other proteins specific for this complex (PTIP/PAXIP1, PA1/PAGR1 and NCOA6).^10 11^ Importantly, sequencing data of human tumours identified truncating mutations of the catalytically active components *KMT2C*, *KMT2D* and *KDM6A* of this complex,^12–16^ suggesting that disruption of their activity can contribute to tumourigenesis. However, despite these observations, mechanistic insights into how these mutations functionally contribute to cancer development and intersect with other pathways involved in tumourigenesis remain largely unknown.

## Results

**Figure 1:**
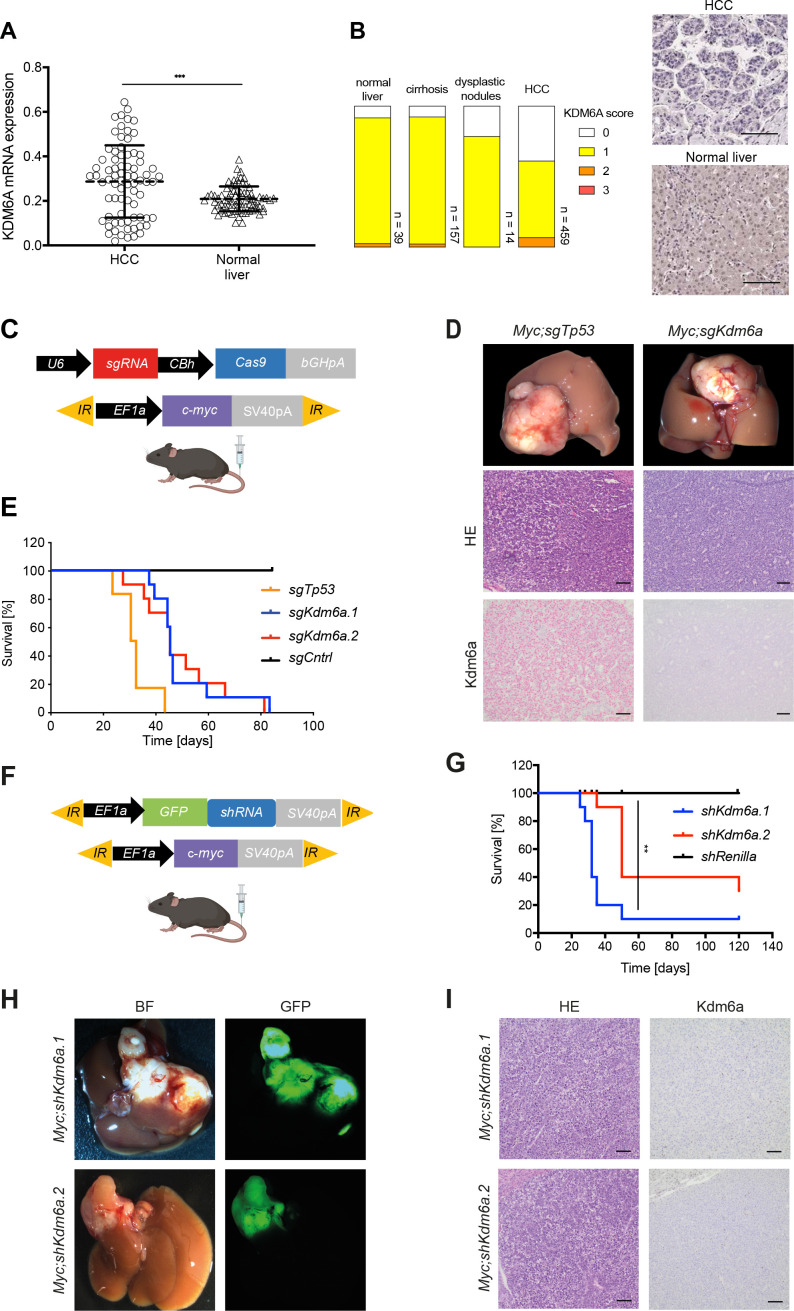
Kdm6a disruption causes liver tumour formation in conjunction with oncogenic Myc. (A) Messenger RNA expression of KDM6A in a clinical cohort of 76 human hepatocellular carcinomas (HCCs). *KDM6A* expression was assessed both in the surrounding healthy liver tissue and tumour tissues. Unpaired t-test, p=0.0001. (B) Left, quantification of KDM6A expression as evaluated by immunohistochemistry in the tissue microarrays classified into normal liver tissues, cirrhotic tissues, dysplastic nodules and HCCs. Right, representative results of KDM6A staining. Image of HCC that scored 0 in the upper panel and normal liver tissue that scored 2 in the lower panel. (C) Vectors permitting transient expression of Cas9 and single guide RNAs (sgRNAs) targeting *Kdm6a* (top), and Sleeping Beauty transposon-based stable expression of c-myc (bottom) were used to generate Myc;sgKdm6a liver tumour via hydrodynamic gene delivery via tail vein injection. (D) Histopathology of liver tumours generated by c-myc overexpression and either *Kdm6a* or *Tp53* knockout. Top, tumour nodules in Myc;sgTp53 and Myc;sgKdm6a injected mice visualised by dissection microscope. Middle, HE staining. Bottom, Kdm6a staining. (E) Survival of mice injected with sgRNAs targeting *Kdm6a* (red and blue line, each n=10), *Tp53* (yellow line, n=6) or green fluorescence protein (GFP) (black line, n=3) as control. (F) Vectors for in vivo short hairpin RNA (shRNA)-mediated gene silencing in the setting of c-myc overexpression. The shRNAs targeting Kdm6a or Renilla luciferase as control, were constitutively expressed and linked to GFP expression. (G) Survival of Myc;shKdm6a mice with two independent shRNA (red and blue line, each n=10) and Myc;shRenilla mice (black line, n=10); log-rank test, **p value=0.0014 (H) Dissection microscope pictures of tumour nodules observed in Myc;shKdm6a mice, note that shRNA-expression in linked to GFP. (I) Histopathology of Myc;shKdm6a liver tumours. Left, HE staining of liver tumours depicted above. Right, Kdm6a staining of corresponding tumours. Scale bars, 50 µm.

To gain a complete overview of genetic alterations of all MLL3/4 COMPASS-like complex members, we mined publicly available pan-cancer sequencing data (www.cbioportal.org 17 18) for truncating mutations, deep deletions and shallow deletions (online supplemental figure 1) and found that indeed the three catalytic subunits showed the highest alteration frequencies (*KDM6A*: 21%, *KMT2D*: 15%, *KMT2C*: 14%). Notably, these three genes showed high numbers of truncating mutations (online supplemental figure 1), indicating that tumours select for loss of function of respective proteins. Considering these genetic events only in *KDM6A*, *KMT2D* and *KMT2C* clearly demonstrated that the highest frequencies are observed in solid cancers (online supplemental figure 2, online supplemental figure 3A), such as gastrointestinal or urological cancer types, whereas functional studies of these genes had previously focused on haematological cancer types.^19–23^ We further analysed the The Cancer Genome Atlas Project (TCGA) data set for hepatocellular carcinoma (HCC), which is an extremely aggressive solid tumour that has emerged as the fourth most frequent cause of cancer deaths worldwide.^24^ HCC is commonly characterised by the frequent amplifications encompassing the *MYC* oncogene, mutations in Wnt-pathway components and/or inactivating mutations in the *TP53* and *CDKN2A* tumour suppressor genes but also exhibits frequently alterations in chromatin modifiers.^25^ Our analyses revealed that similar to other solid tumour types *KDM6A*, *KMT2D* and *KMT2C* showed high frequencies of deep and shallow deletions as well as truncating mutations (online supplemental figure 3B). Notably, *KDM6A* appeared to be the most frequently mutated gene, accounting for 28% of patient samples. Interestingly, *KDM6A* is located on the X-chromosome and was previously shown to escape X-inactivation,^26 27^ thus being a putative monoallelic tumour suppressor exclusively in men. To gain further insights into the role of *KDM6A* in human liver cancer, we analysed *KDM6A* messenger RNA (mRNA) transcript in a cohort containing 76 HCCs and corresponding normal liver tissue (online supplemental table 1). From this cohort, we found that 30% of HCCs expressed lower mRNA levels in comparison to the normal livers (figure 1A) and some HCCs exhibited higher *KDM6A* mRNA levels. Moreover, we probed for KDM6A protein expression in a tissue microarray comprising 39 normal liver tissues and 459 HCCs (online supplemental table 2) and found that nearly all normal liver tissues expressed a detectable but low nuclear expression of KDM6A, whereas KDM6A was absent in approximately 30% of HCCs (figure 1B) and a small proportion of HCC cases showed elevated KDM6A expression. Interestingly, we already observed a decrease in KDM6A expression in dysplastic nodules, the bonafide precursor lesion of HCCs. Thus, our results demonstrate that KDM6A is lost in more than 30% of human HCCs and that this loss could potentially be attributed to genomic deletions of the *KDM6A* locus.

10.1136/gutjnl-2021-325405.supp1 Supplementary data

To determine the functional consequences of Kdm6a loss in liver tumour development, we exploited a powerful mouse model in which genetic elements can be introduced directly into adult wild-type hepatocytes using hydrodynamic gene delivery via tail vein injection (HDTVi). This procedure can introduce cancer predisposing lesions into a subset of hepatocytes using recombinant transposon vectors that permit stable integration of oncogenic complementary DNAs (cDNAs) (transposon vector) or by introducing plasmids encoding Cas9 and single guide RNAs (sgRNAs) that disrupt tumour suppressor genes through genome editing.^28–30^ Strikingly, C57BL/6 mice injected with a transposon vector expressing c-myc (Myc) in conjunction with two independent CRISPR/Cas9 constructs targeting *Kdm6a* (sgKdm6a) (figure 1C) succumbed as rapid from disease as mice injected with CRISPR/Cas9 construct targeting *Tp53* (sgTp53), whereas mice receiving c-myc and a control CRISPR/Cas9 plasmid remained healthy (figure 1D), as described before.^31^ Myc;sgKdm6a and Myc;sgTp53 injected mice developed bonafide HCCs and as expected Kdm6a expression was only absent in Myc;sgKdm6a tumours (figure 1E). Accordingly, T7-endonuclease assay (online supplemental figure 3C) and DNA sequencing (online supplemental figure 3D) of the genomic target region in tumuor-derived cell lines revealed indel mutations in *Kdm6a* and western blot analyses of these cells showed loss of Kdm6a protein (online supplemental figure 3E). Furthermore, sustained suppression by two validated Kdm6a short hairpin RNAs (shRNAs) also cooperated with Myc to produce HCCs on HDTVi, providing further validation of the tumour suppressive action of Kdm6a using an orthogonal approach (figure 1F–I, online supplemental figure 3F). As we also found human HCCs with elevated KDM6A expression, we tested the ability of enforced Kdm6a overexpression to cooperate with c-myc to form liver tumours. However, animals receiving transposon vectors encoding *Kdm6a* cDNA and c-myc did not succumb from disease (online supplemental figure 3G). Therefore, Kdm6a loss cooperates with c-myc to drive liver tumour development.

**Figure 2:**
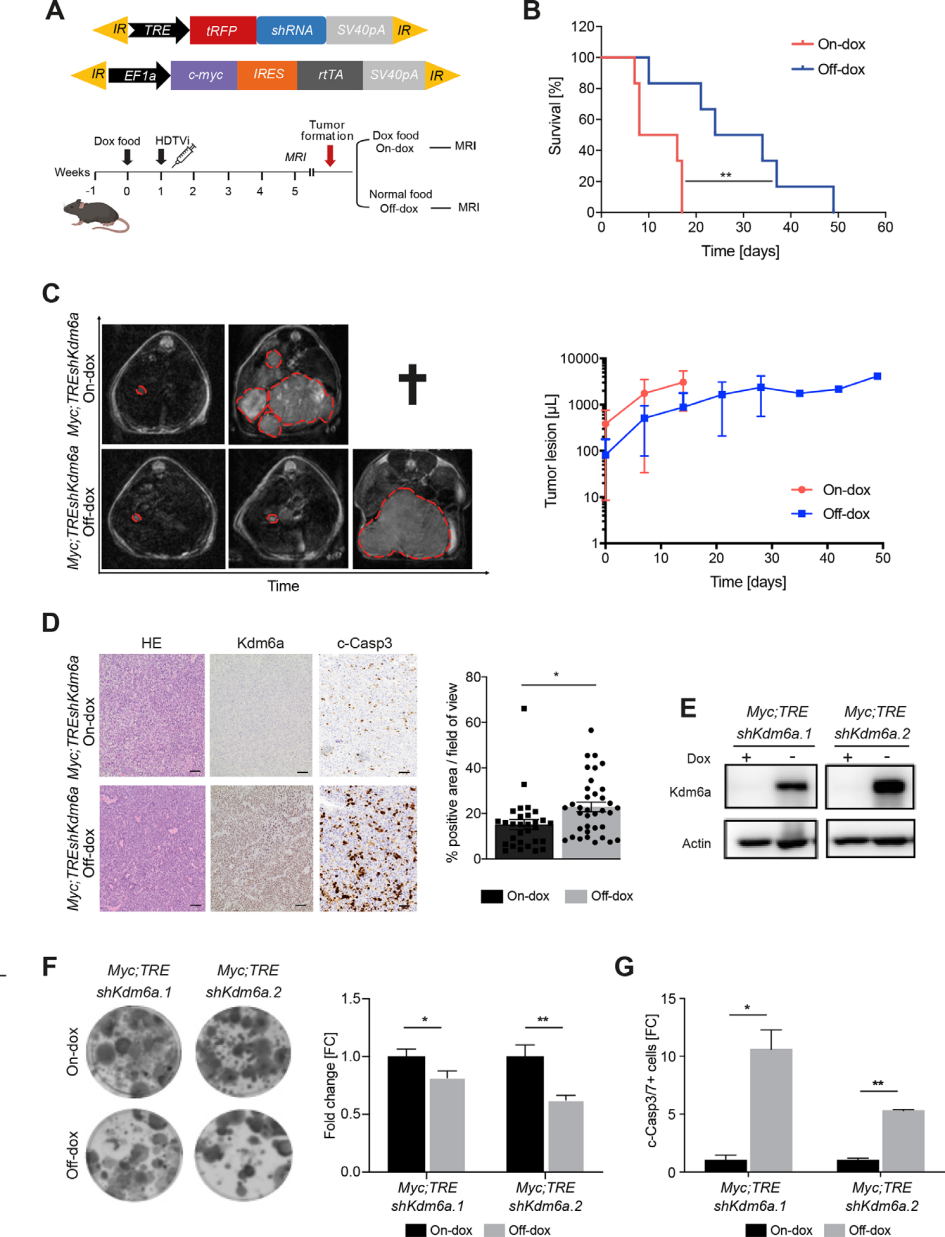
Sustained KDM6A loss is important for tumour maintenance. (A) Schematic view of the Dox-inducible shKdm6a-mediated knockdown mice (Myc;TREshKdm6a) and MRI experimental setting. (B) Survival of Myc;TREshKdm6a mice in the presence (blue line; off-Dox, n=6 mice per group) or absence (red line; on-Dox, n=6) of KDM6A; log-rank test, **p value=0.0067. (C) Left, representative MRI scans of Myc;TREshKdm6a liver tumours (marked in red) at different time points. Right, time course quantification of lesion volume in Myc;TREshKdm6a tumour-bearing mice, (blue line, off-Dox, n=6 mice per group; red line, on-Dox, n=4) (D) Left, representative immunohistochemistry (IHC) stainings of Myc;TREshKdm6a liver tumours. scale bar, 50 µm. Right, quantification of IHC staining for cleaved caspase-3 (c-Casp3). Unpaired t-test, n=6 and 30 fields of view, *p value=0.0136. (E) Immunoblot analyses before (on-Dox) and after (off-Dox) Kdm6a restoration in Myc;TREshKdm6a cell lines expressing two independent Kdm6a shRNAs. Actin served as a loading control. Representative result of n=3. (F) Left, colony formation assay of indicated cell lines with and without Dox for 10 days. Representative result of n=3. Right, quantification of colony formation assay. Values are mean±SD, n=3. Unpaired t-test, *p value=0.022, **p value=0.0085. (G) Active caspase-3/7 labelling in Myc;TREshKdm6a cell lines grown for 6 days with or without Dox. Error bars represent mean±SD; n=3. Paired t-test **p=0.0039 *p=0.0124. Dox, doxycycline; FC, fold change; HDTVi, hydrodynamic gene delivery via tail vein injection.

Next, we asked if sustained Kdm6a suppression is important for tumour maintenance. To this end we injected C57/Bl6 mice with transposon vectors encoding for c-myc and the reverse-tetracycline transactivator (rtTA) and a second transposon vector with expressing a Tet-responsive element (TRE) promoter regulated shRNA targeting Kdm6a linked to turbo red fluorescence protein (tRFP) (figure 2A). Of note, this system allows potent Kdm6a suppression in cells receiving both vectors only when mice are fed doxycycline (Dox) containing food, whereas mice receiving normal chow express endogenous levels of Kdm6a protein. One week before the injection mice were fed with Dox-chow and tumour onset was monitored via MRI (figure 2A). Once tumours were detected, we divided the cohort in two groups: one group further receiving Dox-food (sustained Kdm6a repression) and the second group was placed on normal chow (endogenous Kdm6a reactivation). Remarkably, we found a significant survival benefit of tumour bearing mice on Dox-withdrawal (figure 2B) and analyses of MRI time-courses showed rapid tumour progression in mice with sustained Kdm6a suppression, whereas tumours were stalled in mice with endogenous Kdm6a re-expression, however they eventually progressed at later time points (figure 2C). As expected tumours on-Dox mice expressed tRFP (and thus the shRNA) whereas tRFP was absent in off-Dox tumours (online supplemental figure 4A). Consequently, Kdm6a protein levels were undetectable in on-Dox tumours, but strong nuclear expression of Kdm6a was visible in off-Dox tumours (figure 2D). Although tumour cell proliferation was unchanged between both groups (data not shown), we found a massive induction of apoptosis in off-Dox tumour cells indicated by cleaved-caspase 3 (c-casp3) staining (figure 2D). Using primary cell lines derived from tumours before Dox switch (Myc;TREshKdm6a cells), we observed potent Kdm6a induction on mRNA (online supplemental figure 4B) and protein levels (figure 2E) on Dox withdrawal. Kdm6a re-expressing cells formed fewer colonies (figure 2F) and we detected rapid induction of apoptosis on Kdm6a restoration (figure 2G). Importantly, re-expression of *Kdm6a* cDNA in Myc;sgKdm6a cells (online supplemental figure 5A, B) was accompanied by a disadvantage in cell competition assays (online supplemental figure 5C), reduced colony formation (online supplemental figure 5D, E) and induction of apoptosis (online supplemental figure 5F), thus mirroring the results of endogenous Kdm6a re-expression. Collectively, these experiments demonstrate that sustained Kdm6a suppression protects from apoptosis and is required for tumour maintenance in vivo and in vitro.

**Figure 3:**
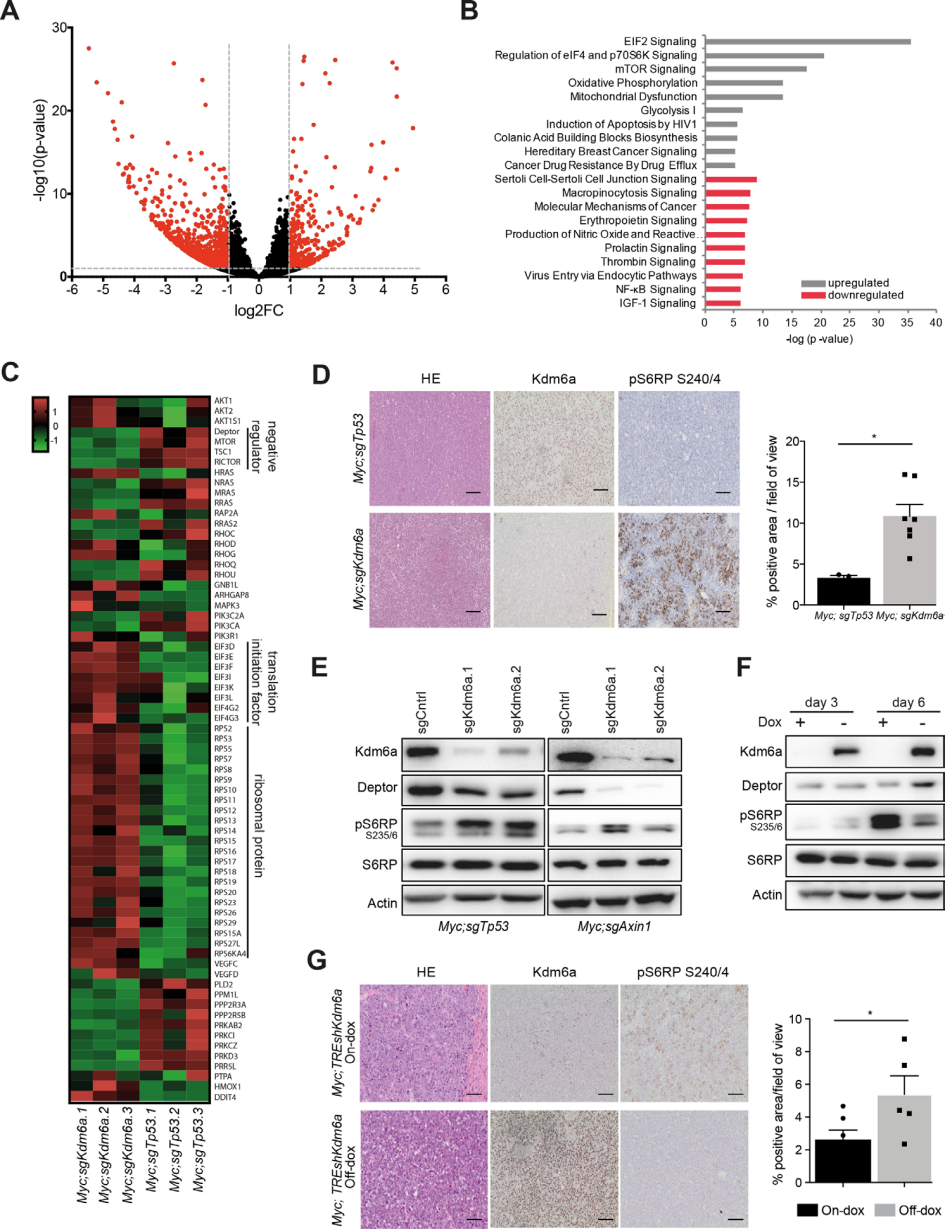
Kdm6a loss leads to distinct transcriptional changes and hyperactivation of mTORC1 signalling. (A) Volcano plot of differentially expressed genes revealed by RNA-seq of Myc;sgKdm6a and Myc;sgTp53 liver tumour-derived cell lines, n=3 independent cell lines per group. Genes with more than twofold expression change and exceeding adjusted p value<0.05 are indicated in red. (B) Top 10 affected canonical pathways from RNA-seq data based on Ingenuity Pathway Analysis. (C) Heatmap of differentially regulated mTOR pathway genes in Myc;sgKdm6a and Myc;sgTp53 cell lines, n=3 independent cell lines per group. (D) Left, representative immunohistochemistry (IHC) images of Kdm6a and pS6RP staining in Myc;sgTp53 and Myc;sgKdm6a murine livers at endpoint of experiment. Scale bar, 50 µm. Right, quantification of IHC staining for pS6RP. Unpaired t-test, *p value=0.0103. (E) Immunoblot analyses of Myc;sgTp53 and Myc;sgAxin1 cell lines expressing either single guide RNAs targeting Kdm6a or empty vector as control; representative results of n=3. (F) Time course immunoblotting of Myc;TREshKdm6a cell line for mTOR signalling pathway in the presence (off-Dox) or absence (on-Dox) of Kdm6a; representative results of n=3. (G) Left, representative images of Kdm6a and pS6RP expression detected by IHC in murine livers at endpoint of experiment in the presence or absence of Dox. Scale bar, 50 µm. Right, quantification of IHC staining for pS6RP. Unpaired t-test, p=0.0494. Dox, doxycycline.

Kdm6a is part of the MLL3/MLL4 COMPASS-like complex, which epigenetically regulates gene expression by influencing chromatin accessibility on histone modifications.^7 11^ Thus, to interrogate potential target genes mediating the tumour suppressive activity of Kdm6a, we profiled the transcriptome of Myc;sgKdm6a cell lines. We found 331 genes upregulated (log2-fold change < −1, p value<0.05) and 910 genes downregulated (log2-fold change >1, p value<0.05) compared with Myc;sgTp53 cell lines (figure 3A), Interestingly, Ingenuity Pathway Analyses revealed upregulation of EIF2 signalling, regulation of eIF4 and p70S6K signalling, and mTOR signalling as the most significant upregulated pathways in Myc;sgKdm6a cells (figure 3B). Closer examination for expression changes of individual components in the mTOR pathway (figure 3C) revealed that negative regulators (*Deptor, Tsc1*) were downregulated across all three Myc;sgKdm6a cell lines, whereas translation initiation factors and ribosomal proteins, which are crucial downstream factors of mTORC1 (online supplemental figure 6A). Quantitative reverse transcription quantitative PCR (RT-qPCR) analyses of Myc;sgKdm6a and Myc;sgTp53 cell lines validated upregulation of *Deptor* in Myc;sgTp53 cell lines (online supplemental figure 6B). Moreover, we could observe a marked mRNA upregulation of *Deptor* 6 days after endogenous Kdm6a restoration in Myc;TREshKdm6a cell lines (online supplemental figure 6C), further indicating a direct relationship between Kdm6a expression levels and transcriptional activation of Deptor in an isogenic setting.

Next, we investigated if the observed transcriptional changes translate into mTORC1 pathway activation. Immunohistochemical analyses of murine Myc;sgKdm6a liver tumours revealed increased phosphorylation of the mTORC1 downstream target S6RP (pS6RP) compared with Myc;sgTp53 liver tumours (figure 3D), indicating hyperactivation of mTORC1 in these tumours. Additionally, Deptor protein was highly abundant in Myc;sgTp53 cells compared with Myc;sgKdm6a cells (online supplemental figure 6D). Strikingly, we observed decrease of Deptor expression and accompanied increase in S6RP phosphorylation on CRISPR/Cas9 mediated knockout of Kdm6a in isogenic murine cancer cell lines derived from Myc;sgTp53 and Myc;sgAxin1 tumours (figure 3E). Furthermore, using Myc;TREshKdm6a cells as an additional isogenic system we found increased protein expression of Deptor as well as decreased pS6RP 6 days after endogenous Kdm6a re-expression (figure 3F). Furthermore, immunohistochemical staining pS6RP of murine liver tumours derived from our previous in vivo reactivation experiments corroborated these results (figure 3G). Hence, these data suggest that Kdm6a can serve as an epigenetic toggle by fostering the transcriptional activation of Deptor resulting in downregulation of mTORC1 signalling.

**Figure 4:**
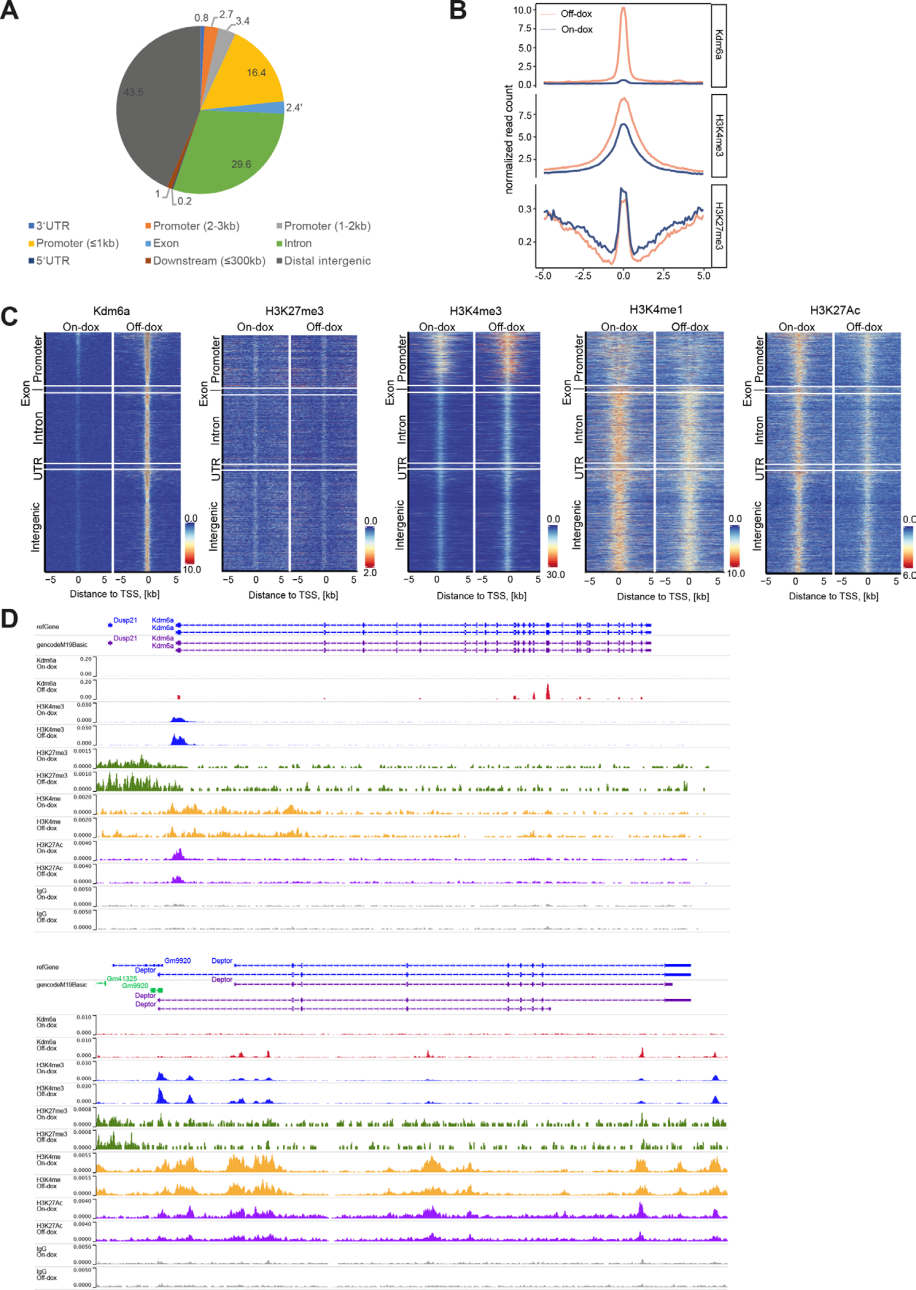
Genome-wide profiling of histone modifications and Kdm6a. (A) Binding sites of Kdm6a genome-wide based on cleavage under target and release using nuclease (CUT&RUN) profiling. (B) Profile plots of Kdm6a, H3K4me3 and H3K27me3 in the proximity of Kdm6a binding sites; n=1. (C) Global heatmaps of Kdm6a and histone modifications (H3K4me3, H3K4me1, H3K27ac, H3K27me3) from CUT&RUN signals in Myc;TREshKdm6a cell line in the presence (off-Dox) or absence (on-Dox) of Kdm6a sorted by Kdm6a signal; n=1. (D) Kdm6a, H3K27ac, H3K4me3, H3K4me1 and H3K27me3 occupancies at *Kdm6a* (top), and *Deptor* (bottom) loci in the Myc;TREshKdm6a liver tumour-derived cell line. TSS, transcriptional start site; UTR, ultranslated region.

Next, to further identify direct targets of Kdm6a we profiled genome-wide binding of Kdm6a. We decided to use Myc;TREshKdm6a with (off-Dox) or without (on-Dox) endogenous Kdm6a re-expression to interrogate direct changes caused by Kdm6a. Cleavage under target and release using nuclease (CUT&RUN) analyses identified 1028 genes enriched in Kdm6a binding 6 days after endogenous Kdm6a re-expression (online supplemental table 3). Interestingly, we found that about 25% of Kdm6a peaks were located close to promoter sites (figure 4A), suggesting Kdm6a could potentially permit transcriptional activation at these genes. Further, we profiled histone marks (H3K27me3, H3K4me1, H3K4me3 and H3K27ac) in cells with (off-Dox) or without (on-Dox) endogenous Kdm6a re-expression and did not identify global changes in these marks. However, when we focused on chromatin regions with Kdm6a binding we could observe reduction of H3K27me3 and gain of H3K4me3 (figure 4B), indicating changes towards permissive chromatin on Kdm6a restoration, particular in promoter regions (figure 4C). Interestingly, we identified strong Kdm6a binding within Kdm6a itself, accompanied by H3K4me3 gain and H3K27me3 loss (figure 4D) and the same changes were also observed in the negative mTORC1 pathway regulator Deptor (figure 4D). Hence, Kdm6a restoration leads to distinct binding of Kdm6a to chromatin accompanied with permissive chromatin changes at promoters, among others in Deptor.

**Figure 5:**
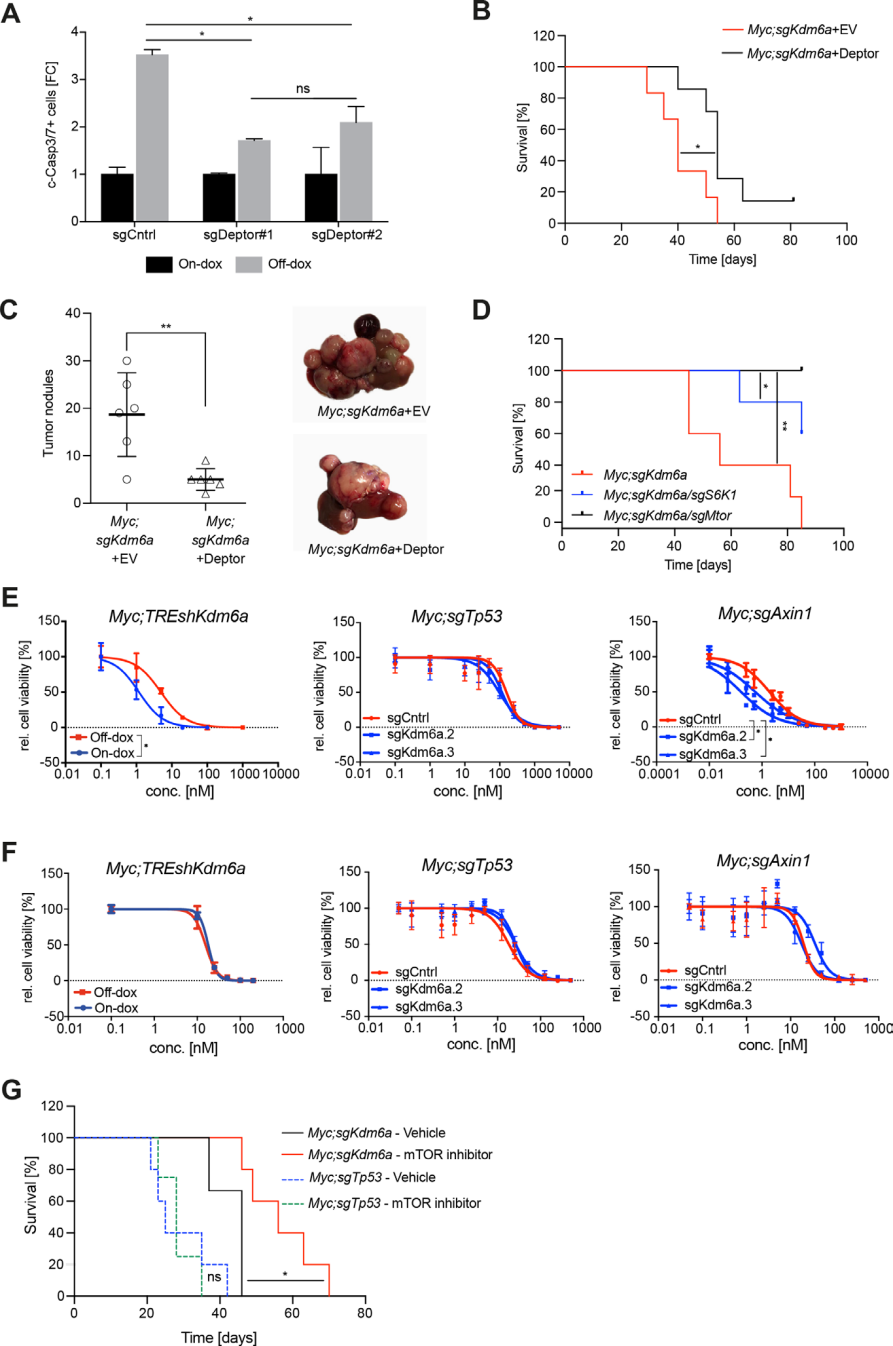
Kmd6a-deficient cells and tumours are dependent on mTORC1 signalling. (A) Caspase-3/7 assay in Myc;TREshKdm6a cells expressing either single guide RNAs targeting *Deptor* or empty vector as control 6 days after endogenous Kdm6a restoration. Error bars represent mean±SD; n=3. Two-way analysis of variance (ANOVA) test, *p value<0.05. (B) Survival curves of Myc;sgKdm6a mice with ectopic Deptor overexpression (black line, n=7) or without ectopic Deptor expression (red line, n=6); log-rank test, *p value=0.0231 (C) Left, quantification of number of tumour nodules of Myc;sgKdm6a mice with ectopic Deptor overexpression (n=7) or without ectopic Deptor expression (n=6). Unpaired t-test, **p value=0.043. Right, representative tumour nodules visualised by dissection microscope. (D) Survival curves of in Myc;sgKdm6a mice (red line, n=6) compared with combined *S6K1* knockout (blue line, n=5) or *Mtor* knockout (black line, n=5); log-rank test, *p value=0.0246, **p value=0.0017. (E) Dose response curve of Torin-1 and (F) Dactolisib in Myc;TREshKdm6a, Myc;sgTp53 and Myc;sgAxin1 cells as analysed by CellTiter-Blue in the presence (red line) or absence (blue line) of Kdm6a. Error bars represent mean±SD. Dose response curves are representative results of n=3 independent experiments. Differences between logIC50 values were determined with unpaired t-test for Myc;TREshKdm6a cell lines and one-way ANOVA for Myc;sgTp53 and Myc;sgAxin1 cell lines, *p value<0.0001. (G) Survival curve of Myc;sgKdm6a mice treated with mTOR inhibitor (rapamycin, red line; n=5) or vehicle (saline, black line; n=3); log-rank test, *p value=0.0274 and Myc;sgTp53 mice with rapamycin (green dotted line, n=4) or vehicle (blue dotted line, n=5); log-rank test, p value=0.8777.

As Deptor is a potent negative regulator of mTORC1 signalling^32–34^ and we identified strong Kdm6a binding as well as pronounced protein expression changes of Deptor on Kdm6a restoration, we next aimed to dissect the role of Deptor in mediating phenotypic effects on Kdm6a restoration. To this end we deleted *Deptor* in Myc;TREshKdm6a cells using CRISPR/Cas9 and monitored these cells following Kdm6a restoration. Consistent with our previous results, re-expression of endogenous Kdm6a in Myc;TREshKdm6a harbouring a control sgRNA induced apoptosis, whereas this apoptotic response was markedly reduced in Myc;TREshKdm6a cells with deletion of *Deptor* (figure 5A). Furthermore, we also designed an in vivo experiment to further validate this causal relationship. Using hydrodynamic delivery, we injected mice with a transposon vector expressing c-myc in conjunction with CRISPR/Cas9 constructs targeting *Kdm6a* and additionally either with a transposon vector expressing *Deptor* cDNA or a control vector. Remarkably, we observed a significant survival benefit (figure 5B) and a significant reduction in tumour nodules in mice with Deptor co-expression compared with controls (figure 5C). Moreover, to clarify the role of mTORC1 signalling in Kdm6a-dependent tumour suppression, we used hydrodynamic delivery of a transposon vector expressing c-myc in conjunction with double CRISPR/Cas9 constructs targeting *Kdm6a* and *Mtor* or *S6K1*, two key downstream molecules of mTORC1 signalling, simultaneously. Whereas mice receiving double CRISPR/Cas9 constructs with Kdm6a and control guide succumbed rapidly from disease, mice with double CRISPR/Cas9 constructs encoding for Kdm6a and Mtor or S6K1 sgRNAs showed prolonged survival and massive reduction in tumour nodules (figure 5D). Thus, Deptor and mTORC1 signalling are crucial determinants for Kdm6a-dependent tumour suppression.

The mTORC1 signalling pathway is aberrantly expressed in many different cancer types and consequently potent inhibitors of this pathway were developed. As our results revealed that Kdm6a-deficient tumours exhibit a high mTORC1 activity, we next tested if they are particularly sensitive to pharmacological mTORC1 inhibition. Indeed, we observed a strong sensitivity to Torin-1, a potent mTORC1 inhibitor, in Myc;sgKdm6a cell lines compared with Myc;sgTp53 cells (online supplemental figure 7A), whereas we could not observe a different response to Dactolisib, a drug inhibiting PI3K (online supplemental figure 7B). Importantly, we observed in an isogenic setting that Myc;TREshKdm6a cells without Kdm6a expression as well as Myc;sgTp53 cells and Myc;sgAxin1 cells with CRISPR/Cas9 mediated *Kdm6a* knockout (sgKdm6a) exhibited remarkable increase in Torin-1 sensitivity (figure 5E), whereas we could not reveal a difference in treatment response with Dactolisib in respect to the Kdm6a status (figure 5F). Inspired by our in vitro observations, we generated mouse cohorts harbouring either autochthonous Myc;sgTp53 or Myc;sgKdm6a liver tumours via hydrodynamic delivery and treated them with rapamycin. As observed in our in vitro experiments, only mice with Myc;sgKdm6a tumours responded to rapamycin treatment, which led to significant prolonged survival (figure 5G). Hence, Kdm6a-deficiency predicts therapeutic response towards mTORC1 inhibitors.

**Figure 6:**
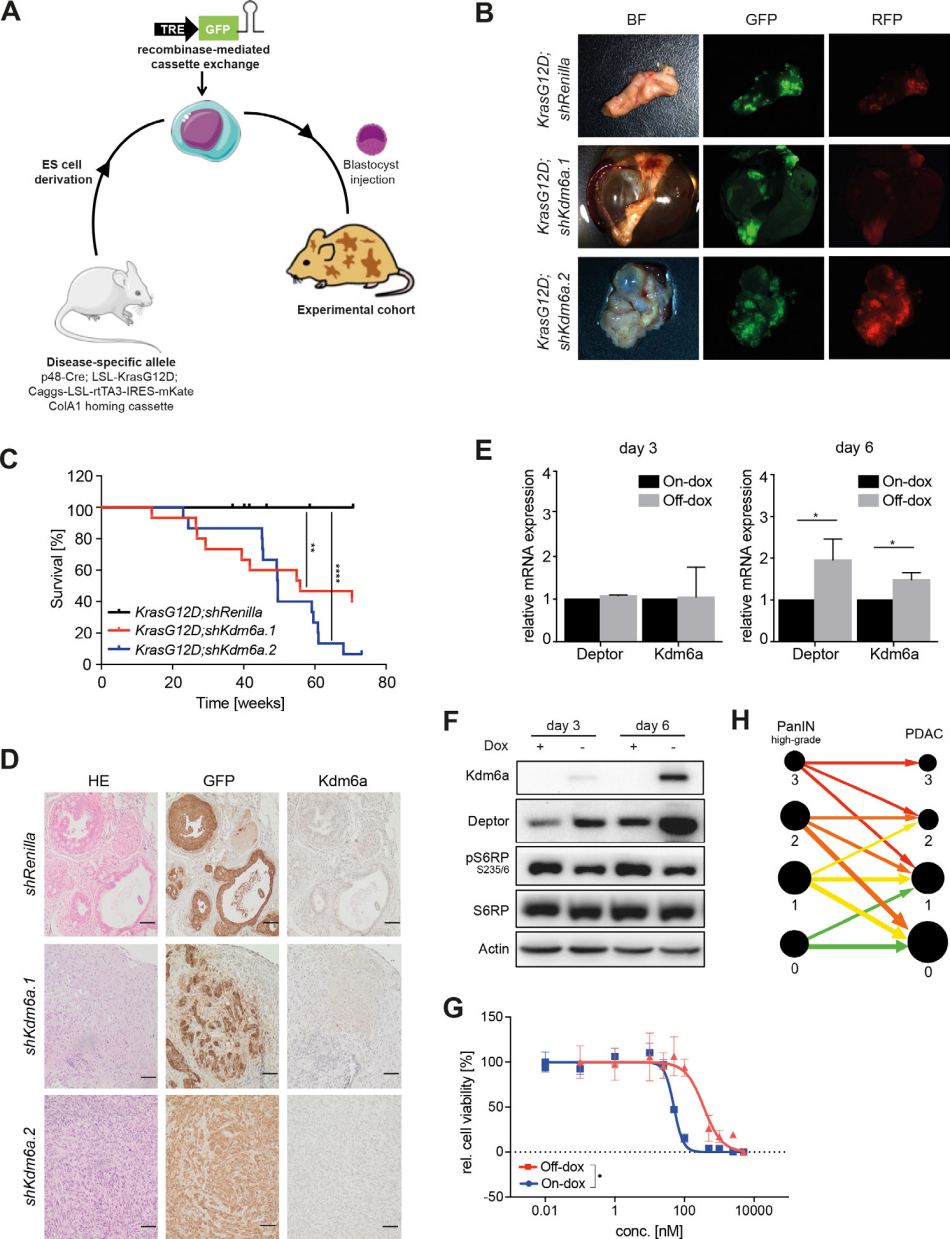
Loss of Kdm6a in combination with oncogenic KrasG12D leads to pancreatic ductal adenocarcinomas. (A) Schematic display of embyonic stem cell (ESC)-based PDAC mouse model generation. (B) Tumour nodules with cyst or normal pancreas observed in KrasG12D;shKdm6a and KrasG12D;shRenilla mice expressing short hairpin RNA (shRNA)-linked green fluorescent protein (GFP) and rtTA3-linked red fluorescent protein (RFP). (C) Survival of KrasG12D;shKdm6a mice (blue and red lines; n=15 for each shRNA) and KrasG12D;shRenilla (black line; n=13) mice as control; log-rank test, **p value=0.0024, ****p value<0.0001. (D) Immunohistochemical staining of HE, GFP and Kdm6a, in KrasG12D;shKdm6a and KrasG12D;shRenilla mice. Scale bar, 50 µm. (E) Quantitative PCR for Kdm6a and Deptor expression in KrasG12D;shKdm6a cell lines in the presence or absence of Dox. Representative results of n=3 independent experiments. Paired t-test, *p value<0.0482. (F) Immunoblot analyses of mTORC1 signalling in KrasG12D;shKdm6a cell line in the presence (off-Dox) or absence (on-Dox) of Kdm6a. Representative result of n=3. (G) Dose response curve of Torin-1 in KrasG12D;shKdm6a cell in the presence (red line; off-Dox) or absence (blue line; on-Dox) of Kdm6a. Error bars represent mean±SD; n=3. Differences between logIC50 values were determined with unpaired t-test, p value>0.0001. (H) Visual representation of changes in KDM6a expression over time in progression from high-grade PanINs to PDAC in clinical cohort of 90 patients. KDM6A expression was scored on the scale from 0 to 3. Size of nodes represents number of patients and size of arrows corresponds to proportional changes in KDM6a levels over time. BF, bright field.

To address the question if the observed effects of Kdm6a are only applicable to liver tumours and only valid in the context of oncogenic c-myc, we additionally created a Kdm6a-deficient mouse model of pancreatic cancer. Harnessing a previously established model of pancreatic cancer that relies on multiallelic embryonic stem cells that harbour a pancreas-specific Cre driver (p-48 Cre), a conditional mutant Kras allele (LSL-KrasG12D), a conditional reverse tetracycline transactivator allele (Caggs-LSL-rtTA3-IRES-Kate) as well as a homing cassette in the ColA1 locus, we inserted TRE-promoter driven and GFP-labelled shRNAs targeting *Kdm6a* (shKdm6a) or Renilla luciferase (shRenilla) using recombinase mediated cassette exchange and generated chimeric mice for further experimental use (figure 6A). Whereas pancreata of mice expressing shRenilla did not exhibit macroscopic abnormalities despite GFP and thus shRNA expression, pancreata of shKdm6a expressing mice showed macroscopically cystic changes and enlargement as well as tumour nodules (figure 6B). Consequently, we observed a significant shorter survival of mice with pancreas-specific Kdm6a suppression (figure 6C). Histologically, we observed bonafide invasive pancreatic ductal adenocarcinomas with infrequent metastatic spread to the liver in shKdm6a expressing mice, whereas only pancreatic intraepithelial neoplasias (PanIN) of different degrees and no invasive tumours were detected in shRenilla mice (figure 6D). As expected tumours showed GFP expression (indicating shRNA expression) and Kdm6a expression was absent in shKdm6a tumour samples (figure 6D). Further, we generated primary cell lines of these tumours and performed similar Kdm6a re-expression experiments as conducted before in liver cell lines. Strikingly, as observed in liver cells endogenous Kdm6a re-expression triggered expression of Deptor and Tsc2 and consequently led reduced phosphorylation of mTORC1 downstream targets (figure 6E, F). Moreover, we also observed a similar shift towards pharmacological mTORC1 inhibitor sensitivity dependent on Kdm6a expression status (figure 6G). Moreover, we examined the expression status of KDM6A in a unique patient cohort comprising matched high-grade PanIN and pancreatic ductal adenocarcinomas (PDAC) of the same patient (online supplemental table 4). Interestingly, we observed that most of the high-grade PanINs showed still retained KDM6A expression and that expression decreased during the progression to PDAC (figure 6H), implicating an active selection for low KDM6A during carcinogenesis. Thus, Kdm6a is a potent tumour suppressor in pancreatic cancer and Kdm6a-deficient pancreatic adenocarcinomas show hyperactive mTORC1 signalling analogous to liver tumours.

**Figure 7:**
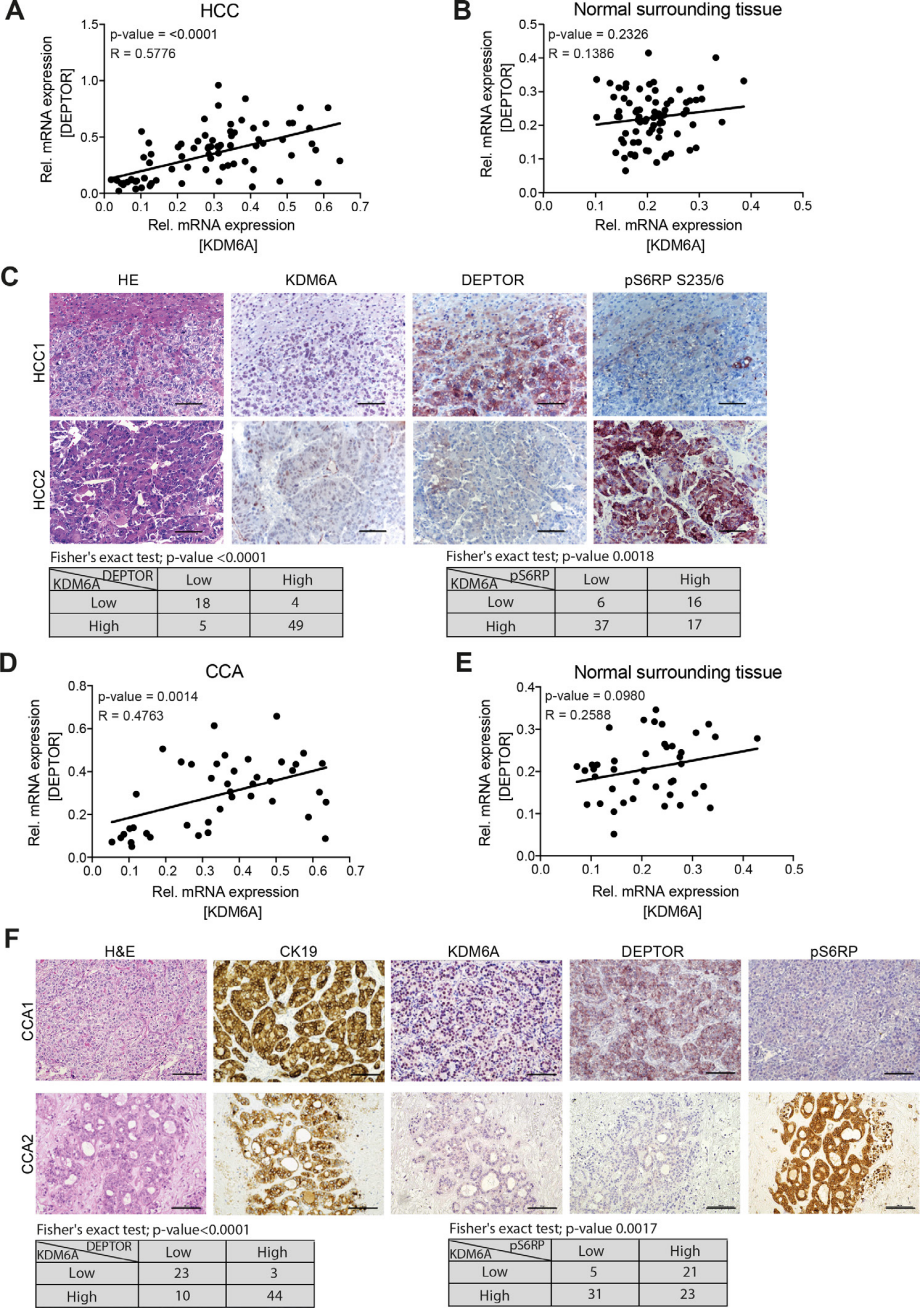
KDM6A expression correlates with DEPTOR expression and activation of mTORC1 in human HCC and cholangiocarcinoma (CCA). Correlation between KDM6A and DEPTOR expression in (A) HCC and (B) in normal surrounding tissue of clinical cohort consisting of 76 human HCCs. (C) Immunohistochemical staining of HE, KDM6A, DEPTOR and pS6RP in two representative patients with HCC. Scale bar represent 100 µm. The significance of the correlation between KDM6A/DEPTOR and KDM6A/pS6RP are shown in the table below using Fisher’s exact test. Correlation between KDM6A and DEPTOR expression in (D) CCA and (E) in normal surrounding tissue of clinical cohort consisting of 80 human CCAs. (F) Immunohistochemical staining of HE, KDM6A, DEPTOR and pS6RP in two representative patients with CCA. Scale bar represent 100 µm. The significance of the correlation between KDM6A/DEPTOR and KDM6A/pS6RP are shown in the table below using Fisher’s exact test. HCC, hepatocellular carcinoma; mRNA, messenger RNA.

Finally, to translate our findings into the human setting, we first correlated mRNA expression levels of *KDM6A* and *DEPTOR* in a cohort of 76 human HCCs and corresponding normal liver samples and found that only in HCC samples *KDM6A* and *DEPTOR* transcripts showed a strong positive correlation (R=0.4236, p=0.0022 vs R=0.04983, p=0.7311) (figure 7A, B). Additionally, we used publicly available transcriptomic data (GEPIA portal) for the TCGA liver cancer data set and confirmed a strong positive correlation between *KDM6A* and *DEPTOR* (R=0.32, p=3.9e^–10^) (online supplemental figure 8A). This correlation was not only confined to liver cancer, as using a pan-cancer data set (GEPIA portal) for *KDM6A* and *DEPTOR* mRNA expression also revealed a significant positive correlation of both transcripts (R=0.25, p=9.1e^–141^) (online supplemental figure 8B). Moreover, we also determined immunohistochemically protein expression in 76 human HCCs for KDM6A, DEPTOR and pS6RP. By classifying tumours in low and high expression levels of individual proteins, we found a significant correlation of low KDM6A expression with low DEPTOR expression and vice versa (figure 7C). Furthermore, tumours with low KDM6A levels were typically associated with high pS6RP levels. Additionally, we performed the same analyses also for a cohort of patients with cholangiocarcinoma (n=80), the second most common liver cancer, and found similar results as observed for HCCs, where low KDM6A protein levels are accompanied with low DEPTOR and high pS6RP levels (figure 7D–F). Collectively, these data show that KDM6A expression correlates with DEPTOR expression in human tumours and is accompanied with activation of mTORC1.

## Discussion

Emerging technological advances to interrogate cancer genomes have pointed us to chromatin modifiers as important components for tumourigenesis.^35 36^ Still, it appears obscure how these genes are functionally involved in this process. By combining powerful genetics, genomics and animal modelling tools as well as human patient data, we surprisingly identify the histone H3K27-demethylase KDM6A as an epigenetic switch for mTORC1 signalling via transcriptional activation of negative pathway regulators in solid cancers. Thus, our results link a poorly understood epigenetic tumour suppressor gene with a major signalling hub in human cancer.

Using publicly available sequencing data of human tumours, we found that in particular the catalytically active components of the MLL3/MLL4 COMPASS-like complex *MLL3*, *MLL4* and *KDM6A* show a high alteration frequency that presumably lead to reduced protein expression. Notably, these analyses revealed that solid cancers have the highest prevalence of these alterations, whereas previous studies that functionally analysed these proteins in tumourigenesis were focusing on haematological malignancies.^19 23 37^ We concentrated on KDM6A, as it showed the highest alteration frequencies, and identified by screening a large amount of human liver cancers that KDM6A protein expression was lost in about 30% of patients. This high incidence could be due to the genomic location of *KDM6A* on the X-chromosome, where a single allelic loss in men could lead to complete loss of expression. Indeed, this phenomenon was previously observed in leukaemias, where KDM6A acts as a gender-specific tumour suppressor.^37^

To interrogate the function of KDM6A in liver cancer we applied a powerful mouse model and found that Kdm6a loss in combination with c-myc leads rapidly to liver tumours and further by establishing an endogenous reactivation system for Kdm6a we show that sustained loss of Kdm6a is important for liver tumour maintenance, thereby clearly underscoring the importance of Kdm6a as a tumour suppressor in liver cancer. We identified apoptosis as one of the main tumour suppressive capacities mediated by Kdm6a and elevated mTORC1 activity on Kdm6a loss. It is widely accepted that aberrant c-myc expression leads to an apoptotic response^38–41^ in healthy tissue preventing transformation and that addition of an additional genetic alteration that blocks the apoptotic response governs rapid oncogenic transformation.^40 42 43^ Interestingly, a synergistic effect of mTROC1 activation and c-myc was previously described in liver tumours and lymphomas.^44 45^ Thus, it seems reasonable that Kdm6a-dependent mTORC1 deregulation is the main driver of transformation in our system.

Transcriptional and epigenetic profiling identified *Deptor* as a target gene of Kdm6a, which is bound by Kdm6a and epigenetically remodelled fostering its transcriptional activation. DEPTOR was initially identified as a crucial negative regulator of mTOR^32^ and it was shown that decreased DEPTOR expression promotes cell growth and survival by mTOR. Interestingly, we found in genetic epistasis experiments that Kdm6a-dependent tumour suppression was largely Deptor dependent, indicating that Deptor is one of the main Kdm6a targets mediating its tumour suppressive abilities. While several genetic alterations leading to aberrant mTORC1 signalling and cancer progression are well-characterised,^46–48^ this is to our knowledge the first study that reveals an epigenetic component that toggles signalling activity of this pathway.

Finally, we were able to translate our findings to patients with cancer. We found that KDM6A expression positively correlates with DEPTOR expression in liver cancer samples. Strikingly, by expanding our murine studies to pancreatic cancer we also found the association between Kdm6a, Deptor and mTORC1 signalling, indicating that these observations are valid beyond liver cancer. Indeed, by mining human patient data, we found a positive correlation between KDM6A and DEPTOR also in other solid cancer types. Although KDM6A undoubtedly controls other cancer relevant genes and pathways beyond mTORC1, our data reveal that KDM6A-deficient tumours are sensitive to mTORC1 inhibition and suggest that KDM6A could serve as a biomarker for therapies focussing on mTORC1. It will be important to determine in future experiments, if this relationship between KDM6A and mTORC1 is only restricted on cancer cells or is also important in normal physiology. Interestingly, it was recently shown that metformin can directly bind to KDM6A and it is tempting to speculate that this could influence mTORC1 signalling and therefore explain some of the mechanistic mode of action of metformin treatment.^49^ One avenue for addressing the function of KDM6A in normal physiology could be patients suffering from Kabuki syndrome, which have a germline deletion in KDM6A.^50^

Collectively, our results reveal an unanticipated epigenetic mechanism that connects a somatically mutated chromatin modifier to a well-characterised signalling pathway network with a major role in cancer.

## Materials and methods

### Molecular cloning

For CRISPR/Cas9-mediated genome editing, sgRNAs were subcloned into pX330 or pLentiCRISPR v2 according to Feng Zhang protocol.^51^ Briefly, BbsI-digested pX330 or pLentiCRISPR v2 were ligated to the annealed and phosphorylated sgRNA. All constructs were subjected to Sanger sequencing with human U6 primer before used in the study.

For shRNA cloning, potent shRNAs were predicted using the algorithm by Pelossof *et al* 52 and cloned into the MLPe vector (MSCV-LTR-miR-E-PGK-Puro-IRES-GFP) as described before.^53^ The hairpins were ordered as 97-mer oligos and were PCR amplified with miRE-XhoI and miRE-EcoRI primer. The PCR product was then digested with EcoRI-HF and XhoI and purified before ligated to the EcoRI-HF/XhoI-digested MLPe backbone. To evaluate the knockdown efficiency of the shRNAs, target cells were transduced with a multiplicity of infection (MOI) <0.7 to achieve single copy integration. After puromycin selection cells were harvested and subjected to western blot. Knockdown efficiency was compared with cells transduced with Renilla luciferase, a non-targeting control. Two most potent shRNAs against *KDM6A* were chosen and further cloned into pT3-TRE-tRFP-miR-E and pCol-TGM. Both plasmids were also digested with EcoRI-HF and XhoI, and ligated to the EcoRI-HF/XhoI-digested PCR amplicons (hairpin). All plasmids were sequenced with miR-E primer before used in the study. Oligonucleotides sequences of sgRNA, shRNA and primers are listed in online supplemental table 5.

For overexpression plasmid, pT3-EF1a-MYC-IRES-Deptor was constructed by PCR amplification of Deptor from Addgene plasmid 21 334 and was cloned into pT3-EF1a-MYC-IRES-rtTA3, which was digested with MscI and XmaI with NEBuilder HiFi DNA Assembly (NEB) according to the manufacturer’s protocol.

### Animal experiments

The group size for individual animal experiments was determined on the basis of our experience with previous similar experiments. For hydrodynamic tail vein injections, 8 weeks old female C57Bl/6 animals were purchased from Envigo. 5 µg DNA of pT3-EF1a-MYC, 20 µg of pX330 expressing indicated sgRNAs together with CMV-SB13 Transposase (1:5 ratio); 20 µg of pT3-TRE-tRFP-shRNA, 5 µg of pT3-myc-IRES-rtTA3 and CMV-SB13; 5 µg of pT3-EF1a-MYC-IRES-Deptor, 20 µg of pX330 sgRNA and CMV-SB13 were prepared in a sterile 0.9% Sodium chloride (NaCl) solution and injected into the lateral tail vein with a total volume corresponding 10% of body weight in 5–7 s as described before.

All animals were monitored daily and animal experiments were performed in compliance with all relevant ethical regulations determined in the animal permit. On euthanasia, relevant organs from experimental mice were visually inspected, harvested and photographed. Tumour samples were taken to obtain genomic DNA, RNA, protein and the rest were incubated in 4% paraformaldehyde for at least 24 hours for further use. All animal experiments were approved by the regional board Karlsruhe, Germany.

### ESC-based pancreatic cancer

Embryonic stem cells (ESCs) harbouring disease predisposing alleles as described before were used to introduce the conditional *KDM6A* knockdown by generating pCol-TGM plasmids containing the shRNA. Two shRNAs against *KDM6A* and one control shRNA against Renilla luciferase were cloned into pCol-TGM plasmid and were electroporated into the ESCs together with pCAGs-FLPe to mediate recombinase-mediated cassette exchange. After electroporation ESCs were selected for hygromycin resistance and only ESCs with successful integration of the targeting construct at the ColA1a locus confer hygromycin resistance. After selection, resistant clones were picked and expanded. Targeted clones were used for blastocyst injections (in cooperation with the DKFZ transgenic core facility) to generate cohorts of chimeric mice, which were directly used for further experiments. Mice received Dox-containing diet at the age of 4 weeks in order to activate shRNA expression and thus *KDM6A* knockdown. Disease onset was monitored by weekly palpation.

### MRI

MRI was carried out by our small animal imaging core facility in DKFZ using a Bruker BioSpec 1Tesla (Ettlingen, Germany). For the imaging, mice were anaesthetised with 3% sevoflurane in air. T2-weighted imaging were performed using a T2_RARE_ sequence axial: TE=84 ms, TR=4806.1582 ms, FOV 30×30 mm, slice thickness 1 mm, averages=4, Scan Time 461.39 s, echo spacing 8 ms, rare factor 10, slices 20, image size 192×192, flip angle 180. If liver lesions can be observed in T2, contrast-enhanced T1 measurement (80 µl ProHance, 0.5 mmol/kg, Bracco, intraperitoneal injection) were carried out to visualise and quantify tumour growth. Unfortunately, the liver tumours did not accumulate the contrast reagent and thus a volumetric size determination with T1 was not possible. The size determination was then performed using T2-weighted MRI images. The region of interest (ROI) were drawn manually in each layer and total volume of the lesion from the individual ROI was calculated with ParaVision software (Bruker). The evaluation was carried out by the same person throughout the study.

### Immunohistochemistry

Samples were fixed in 4% paraformaldehyde for at least 72 hours, embedded in paraffin and sliced into 2 µm thick sections for immunohistochemistry (IHC) staining.

For Kdm6a and GFP staining in mouse tissue, slides were deparaffinised with xylene, rehydrated through a descending alcohol series and washed in water. For antigen retrieval, slides were put in sodium citrate buffer (pH 6.0) and boiled in a pressure cooker for 8 min followed by cooling down under running water for 5 min. Subsequently slides were blocked with 3% hydrogen peroxide for endogenous peroxidase activity, washed for 1 min under running water and twice with phosphate-buffered saline (PBS) and blocked with 5% bovine serum albumine (BSA) in PBS with 0.05% Triton X-100 at room temperature for 1 hour. Slides were incubated with the rabbit monoclonal anti-KDM6A overnight at 4°C. Slides were then washed three times with PBS+0.05% Triton X-100, incubated with ImmPRESS goat anti rabbit or mouse IgG Polymer Kit, peroxidase reagent (Vector Laboratories #MP-7451) for 30 min at room temperature and washed further twice with PBS+0.05% Triton X-100. Staining was visualised using ImmPACT DAB Peroxidase Substrate Kit (Vector Laboratories #SK-4105) according to the manufacturer’s protocol and were counterstained with haematoxylin. Finally, the slides were washed through ascending alcohol series that ended with xylol and mounted with Surgipath Micromount Mounting Medium (Leica # 3801731).

For c-casp3 and both pS6RP staining in mouse tissue, the BOND-MAX (Leica Biosystems) was used to carry out automated IHC staining of slides. Antigen retrieval was carried out with BondTM citrate solution (AR9961, Leica) or BondTM EDTA solution (AR9640, Leica). Thereafter sections were incubated with the specific antibodies against antigens in BondTM primary antibody diluent (AR9352, Leica Biosystems). Primary antibody exposure was followed by secondary antibody (Leica Biosystems) and staining using the Bond Polymer Refine Detection Kit (DS9800, Leica Biosystems). For quantification of stainings, slides were scanned using a SCN400 slide scanner (Leica Biosystems) at 20× magnification.

For human tissues, liver specimens were fixed overnight in 4% paraformaldehyde and embedded in paraffin. Sections were done at 5 µm in thickness. For immunohistochemical staining, slides were deparaffinised in xylene, rehydrated through a graded alcohol series and rinsed in PBS. Antigen retrieval was performed in 10 mM sodium citrate buffer (pH 6.0) by placement in a microwave oven on high for 10 min, followed by a 20 min cool down at room temperature. After a blocking step with the 5% goat serum and Avidin-Biotin blocking kit (Vector Laboratories, Burlingame, California, USA), the slides were incubated with the primary antibodies overnight at 4°C. Slides were then subjected to 3% hydrogen peroxide for 10 min to quench endogenous peroxidase activity and, subsequently, the biotin conjugated secondary antibody was applied at a 1:500 dilution for 30 min at room temperature. The immunoreactivity was visualised with the Vectastain Elite ABC kit (Vector Laboratories) and Vector NovaRed (Vector Laboratories) as the chromogen. Slides were counterstained with haematoxylin. Slides were evaluated semi-quantitatively by comparing each tumour with its surrounding non-neoplastic counterpart, thus defining ‘high’ and ‘low’ the levels of a given protein in a HCC sample when compared with the corresponding non-tumourous counterpart.

### Derivation of primary liver tumour cell lines

Liver tumours were resected with sterile instruments and washed in sterile PBS prior to digestion. Then tumour tissue was minced and resuspended in a mix of 4 mg/mL collagenase intravenous and dispase (w/v in sterile, serum free Dulbecco’s Modified Eagle Medium (DMEM, Sigma)) at 37°C for 30 min with gentle shaking. The dissociated tumour cells were then washed with complete DMEM (supplemented with 10% (v/v) fetal bovine serum and 1% penicillin/streptomycin) and plated on collagen-coated plates (PurCol, Cell Systems; 0.05 mg/mL). Primary cultures were passaged until visibly free from other contaminating cell types.

### Derivation of primary pancreatic tumour cell lines

Pancreatic tumours were dissected with sterile instruments, washed in sterile Hanks balanced salt solution (HBSS) and were minced with a blade until chunks were about 1–2 mm. Tumour tissues were resuspended in 1 mg/mL collagenase V (Sigma) (w/v in sterile, serum free HBSS with Ca^2+^/Mg^2+^) and incubated at 37°C for 30 min with gentle shaking. The dissociated tumour cells were then washed with PBS, resuspended in 0.25% trypsin and incubated at 37°C for 5 min to break up some extracellular matrix. Trypsin was then neutralised with complete DMEM. Before plated on collagen-coated plates, cells were further washed twice with complete medium. Primary cultures were passaged until the GFP positive population >90%.

### Cell culture

All cell lines were maintained in complete DMEM at 37°C with 5% carbon dioxide. Liver and pancreatic cancer cells were split twice per week at a ratio of 1:30–40 and 1:5 correspondingly using collagen-coated plates. Myc;shKdm6a and KrasG12D;shKdm6a cells were cultivated in complete DMEM, with Dox (VWR; 1 µg/mL) when applicable.

### Virus production

For lentivirus production, HEK293T cells were plated 1 day before transfection into 10 cm plates and transfected when nearly full confluence was reached using a plasmid mix of 2.5 µg pMD.2G, 8 µg psPAX2 (Addgene plasmid #12 259 and # 12260, both were a gift from Didier Trono) and 10 µg pLenti CRISPR v2 harbouring respective guides in 1000 µl serum-free DMEM and 60 µl polyethylenimine (PEI, 1 µg/µl). The plasmid mix was then vortexed for 5 s, incubated at room temperature for 30 min incubation and added drop-wise to cells. Twenty-four hours following the transfection, medium was exchanged and lentiviral supernatant was harvested 48 hours post-transfection using 0.45 µm Cellulose Acetate Membrane filters (VWR) and stored at −80°C until use.

For retrovirus production, HEK-gp-cells were also seeded out as for lentivirus production and transfected using a plasmid mix of 2.5 µg pMD.2G and 20 µg retroviral plasmid such as MLPe vector, pSIN or pMSCV-rtTA3-PGK-Puro in 1000 µl serum-free DMEM and 60 µl PEI (1 µg/µl). The viruses were harvested as lentivirus.

### Transduction

Target cells were plated on 10 cm plate and 1 day following the plating, cells were transduced with viral supernatants in the presence of polybrene (4 µg/mL). Two days post-transduction cells were selected with puromycin (2 µg/mL), blasticidin (10 µg/mL) or G418 (neomycin; 1 mg/mL) dependent on the plasmid.

### Immunoblotting

Cells were harvested and lysed in cell lysis buffer (Cell Signaling Technology) supplemented with both protease (Complete Mini; Roche) and phosphatase inhibitors. To ensure lysis, cells were sonicated for 5 min in ice and subsequently centrifuged at 4°C at 13 000 rpm to collect protein lysates. Furthermore, protein lysates were equalised using BCA protein assay (Thermo Scientific), equal amount of protein were mixed with Laemmli buffer (100 mM Tris-HCl pH 6.8, 5% glycerol, 2% sodium dodecyl sulfate (SDS), 5% 2-mercaptoethanol) and boiled at 95°C for 5 min. Proteins were separated by SDS-PAGE, transferred onto polyvinylidene fluoride (PVDF) membrane, and detected by immunoblotting using the appropriate antibodies. Image detection was performed with AlphaView software (ProteinSimple) using the Clarity Western ECL substrate Solution (Bio-Rad). The list of antibodies and their sources can be found in online supplemental table 6.

### Mutation detection by T7 assay

CRISPR/Cas9-mediated mutations were detected using the T7 Endonuclease I (New England Biolabs). Briefly, genomic DNAs was isolated using Gentra Puregene Tissue Kit (Qiagen) in accordance to manufacturer’s protocol. An approximately 700 bp region surrounding the CRISPR/Cas9-targeted site was amplified using the Q5 Hot Start DNA Polymerase (New England Biolabs), column-purified (Qiagen) and subjected to a series of melting and annealing cycles with the annealing temperature gradually lowered in each successive cycle. T7 Endonuclease I was then added to selectively digest heteroduplex DNA. Digest products were visualised on a 2%–3% agarose gel.

Alternatively, Sanger nucleotide sequencing analysis was performed on PCR products using a T7 primers to detect mutations.

### Clonogenic assay

To assess cell survival and proliferation, 500 cells were plated in 6-well plates as triplicate. Both tetracycline inducible-shKdm6a or Kdm6a-cDNA expressing cells were grown in the presence or absence of doxycycline, and cells were fixed with methanol and stained with 0.05% crystal violet after 10 days.

For quantification, depending on confluency of the plates, two methods were used. Either, the amount of crystal violet taken up by the cells were dissolved in solubilisation buffer (50% methanol, 5% acetic acid and 0.1% SDS) and quantified in a spectrophotometer by reading the absorbance at 570 nm or with an ImageJ Plugin.^54^

### Competition assay

To evaluate the effect of Kdm6a overexpression in cell proliferation/viability, Myc;sgKdm6a cells expressing rtTA3 (GFP negative) were mixed with either GFP-positive Kdm6a overexpressing or control cells with 30:70 ratio. The GFP^+^/GFP^−^ ratio were evaluated with Guava easyCyte benchtop flow cytometer (Merck Millipore) over time in the presence or absence of Dox.

### Apoptosis assay

Apoptosis was assessed in liver cancer cell lines via CellEvent Caspase-3/7 Green Detection Reagent (Invitrogen) according to manufacturer’s protocol. Briefly, 10 000 cells were grown with and without Dox for 6 days in 6-well plate, trypsinised and resuspended in complete DMEM. About 100 000 cells were incubated with 2 µM final concentration of CellEvent Caspase-3/7 Green Detection Reagent for 45 min at 37°C and subsequently analysed with Guava flow cytometer.

### Drug treatment and cell viability assay

For assessment of cell sensitivity towards Torin-1 (Cayman Chemical), cells were plated in 96-well plate 1 day prior to treatment and cell viabilities were assessed with CellTiter-Blue (Promega) 72 hours following the drug treatment. The reagent was added into each well of 96-well plate with 1:10 dilution, incubated for 4 hours at 37°C and fluorescent signal was recorded (560_Ex_/590_Em_) using FLUOstar Omega (BMG Labtech).

### Quantitative reverse transcription PCR

Total RNA was isolated using RNeasy Mini Kit (Qiagen) and RNase-Free DNase Set (Qiagen) in accordance to manufacturer’s protocol. Purified RNA 1 µg was reverse transcribed using TaqMan Reverse Transcription Reagents (Thermo Fisher Scientific) and diluted 1:20 before subjected to qPCR. For the qPCR reaction, cDNA was mixed with Power SYBR Green Master Mix (Thermo Fisher Scientific) and target-specific primers and performed in triplicate. Transcript levels were normalised to the levels of *tubulin* mRNA expression and calculated using the deltaCt (ΔCt) method. qPCR was carried out using QuantStudio 3 Real-Time PCR system (Applied Biosystems).

For human patient quantification, Gene Expression Assays for human UTX/KDM6A (ID# Hs00253500_m1), Deptor (Hs00961900_m1) and β-Actin (ID # 4333762T) genes were purchased from Applied Biosystems (Foster City, California, USA). Quantitative values for each gene were calculated by using the PE Biosystems Analysis software and expressed as number target (NT). NT=2−ΔCt, wherein ΔCt value of each sample was calculated by subtracting the average Ct value of the target gene from the average Ct value of the *β-Actin* gene.

### RNA sequencing and differential expression analysis

For RNA sequencing, total RNA from three independent tumour-derived cell lines (Myc;sgMll3 and Myc;sgTp53) was isolated using RNeasy Mini Kit (Qiagen), QIAshredder Columns and RNase-Free DNase Set (Qiagen). RNA-seq library construction and sequencing were performed according to protocols used by the integrated genomics operation Core at Memorial Sloan Kettering Cancer Center (MSKCC). From each replicate sample 5–10 million reads were acquired. After removing adaptor sequences with Trimmomatic,^55^ RNA-seq reads were aligned to GRCh37.75(hg19) with STAR.^56^ Genome-wide transcript counting was performed by tool for the analysis of high-thoughput sequencing data (HTSeq) to generate a fragments per kilobase per million mapped reads (FPKM) matrix.^57^ Differentially expressed genes were identified by DESeq2 (V.1.8.2, package in R) and plotted in the volcano plot. The complete data set is available at NCBI Gene Expression Omnibus GSE155630.

### CUT&RUN

CUT&RUN was performed as previously described by Skene *et al*.^58^ Briefly 250 000 Myc;TREshKdm6a cells grown in the presence or absence of Dox were harvested and immobilised on activated concanavalin A—coated beads at room temperature for 10 min, permeabilised with 0.025% digitonin and incubated with antibody with rotation overnight at 4°C. All the antibodies used in this study were diluted 1:100 for CUT&RUN experiment and are listed in online supplemental table 3. Following the incubation, cells were incubated with the pA-MNase to a final concentration of 700 ng/mL at 4°C for 1 hour, washed and digested on Ca^2+^ addition at 0°C for 30 min. To release the DNA-protein complex, cells were further incubated at 37°C for 10 min and the supernatant was collected. The DNA fragments in the supernatant were then extracted using the spin column (Qiagen) and libraries were prepared using NEBNext Ultra II DNA Library Kit for Illumina (New England Biolabs) following the manufacturer’s protocol. The libraries were sequenced using HiSeq2500 (Illumina) in 25 bp paired-end rapid mode (library concentration of flowcell: 12pM, 1% PhiX spike-in) and HiSeq Rapid SBS Kit v2 (50 cycles; FC-402–402) was used for the HiSeq PE 25 R sequencing type. The complete data set is available at European Nucleotide Archive with accession ID PRJEB39876.

### CUT&RUN processing and analysis

Adapter and quality trimming of raw sequencing reads was performed using Trim Galore V.0.4.4^59^ in conjunction with Cutadapt V.1.14^60^ and the non-default parameters ‘--length_1 35’, and ‘--length_2 35’, ‘--paired’, and ‘--illumina’. Bowtie2 with the ‘--very-sensitive’” flag^61^ was deployed to separately align trimmed reads to both the Genome Reference Consortium Mouse Build V.38 and the Saccharomyces cerevisiae R64 reference genome. Removal of PCR duplicates was performed by means of Picard MarkDuplicates V.2.17.4.^62^ Unpaired alignments as well as mappings with a quality below 20 on the Phred scale were filtered out using SAMtools view V.1.5.^63^ Filtered alignments to the R64 genome were counted. The minimal yeast alignment count observed among all libraries of a specific antibody target was determined. Library-specific scaling factors were calculated by dividing minimal yeast alignment counts by the library-specific count. Coverage tracks were generated by deploying the bamCoverage functionality of deepTools V.3.1.1^64^ with the non-default parameters ‘--effectiveGenomeSize 2652783500’ and ‘--ignoreForNormalization chrM chrY chrX’. Additionally, the scaling factors were included via the ‘--scaleRatio’ option. For peak calling, MACS2 callpeak V.2.1.0.20140616^64^ was used with an false discovery rate (FDR) cut-off of 0.05 and the parameters ‘--nomodel’, ‘--format BAMPE’, ‘--gsize 2652783500’, ‘--keep-dup all’ and optionally the ‘--broad’ flag for the histone marks H3K4me1 and H3K27me3. The data processing procedure was implemented as a fully containerised pipeline using the Common Workflow Language V.1.0^65^ and is publicly available.^66^

UTX peaks were associated with the closest TSS using the mouse gene model annotation information from R/Bioconductor packages TxDb.Mmusculus.UCSC.mm10.knownGene (V.3.10.0) and BSgenome.Mmusculus.UCSC.mm10 (V.1.4.0). Association with gene model features was performed using package ChIPseeker (V.1.24.0).^67^ Coverage at genomic features was summarised using bwtool suite^68^ and visualised as profile plots and heatmaps using custom R code. For the global analysis of histone marks at TSSs, the read coverage at a 2 kb radius around TSSs obtained from Gencode Mouse Release 23 was summarised using the Genomation package (V.1.12.0).^69^ Empirical cumulative distribution functions for coverage at TSSs being close (<3 kb) or not close to Kdm6a peaks were estimated and visualised using the stats_ecdf functionality of ggplot2.^70^

### Human patient samples

Seventy-six HCCs and corresponding surrounding non-tumour liver tissues were used for the study. Liver tissues were collected at the Universities of Greifswald (Greifswald, Germany) and Regensburg (Regensburg, Germany).

Pancreatic cancer samples were collected from patients undergoing surgery at the University Hospital Mainz (Mainz, Germany). Samples from 84 patients were included in the study from which both high-grade PanINs and PDAC samples were available.

### Human HCC tissue microarray

The HCC tissue microarray used in this study contained 720 representative tissue cores (diameter: 1 mm) distributed on seven slides. In total, 40 histologically normal livers, 174 cirrhosis, 14 dysplastic nodules and 476 HCCs were spotted (87× G1, 311× G2, 76× G3, 2× G4 HCCs). For the evaluation of individual immunohistochemical stains, the KDM6A intensity were evaluated. Staining intensity was scored from 0 to 3; 0=unstained, 1=weakly, 2=moderately and 3=strongly positive.

## Data Availability

Data are available in a public, open access repository. Data are available upon reasonable request. Memorial Sloan Kettering Cancer Center,
