# Supplementary material for: Histone H3K27 demethylase KDM6A is an epigenetic gatekeeper of mTORC1 signalling in cancer
Source: Gut. 2021 Sep 11;71(8):1613–28. doi: 10.1136/gutjnl-2021-325405 (PMC9279849; doi:10.1136/gutjnl-2021-325405)
Supplement: Supplementary data [file gutjnl-2021-325405supp001.pdf]

Supplementary Materials

The histone H3K27 demethylase KDM6A is an epigenetic gatekeeper of mTORC1 signalling in cancer

Steffie Revia[1], Agnieszka Seretny[1], Lena Wendler[1], Ana Banito[2], Christoph Eckert[1], Kersten Breuer[3], Anand Mayakonda[3], Pavlo Lutsik[3], Matthias Evert[4], Silvia Ribback[5], Suchira Gallage[6], Ismaiel Chikh Bakri[7], Kai Breuhahn[7], Peter Schirmacher[7], Stefan Heinrich[8], Matthias M. Gaida[9,10,11], Matthias Heikenwälder[6], Diego F. Calvisi[4], Christoph Plass[3], Scott W. Lowe[12,13], Darjus F. Tschaharganeh[1, *]

This file includes:

Supplementary Figures 1-8

Supplementary Tables 1-6

Contact information:

Darjus Tschaharganeh, MD

Email: d.tschaharganeh@dkfz.de

Phone: +49 6221-5634555

German Cancer Research Center (DKFZ)

Im Neuenheimer Feld 224

69120 Heidelberg, Germany

Supplementary Figure 1

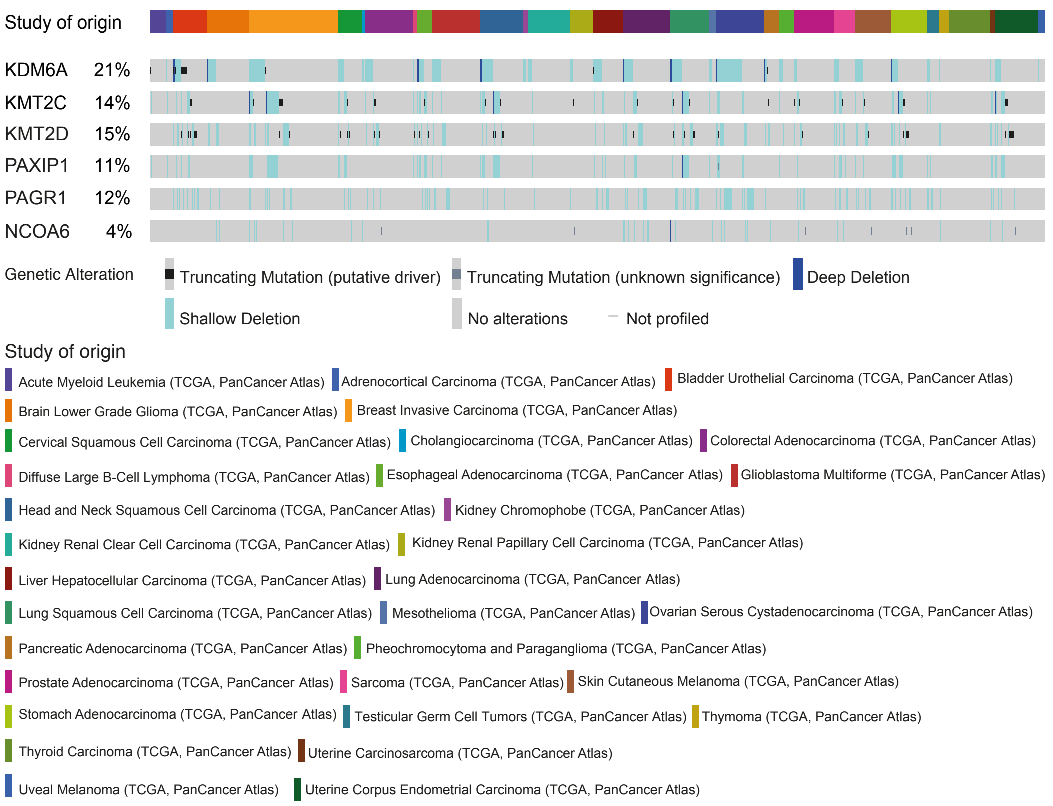

Suppl. Fig 1 deletions and mutations of MLL3/MLL4 COMPASS-like complex components in publicly available TCGA data sets across various cancer types as illustrated by OncoPrints (cBioportal).

Supplementary Figure 2

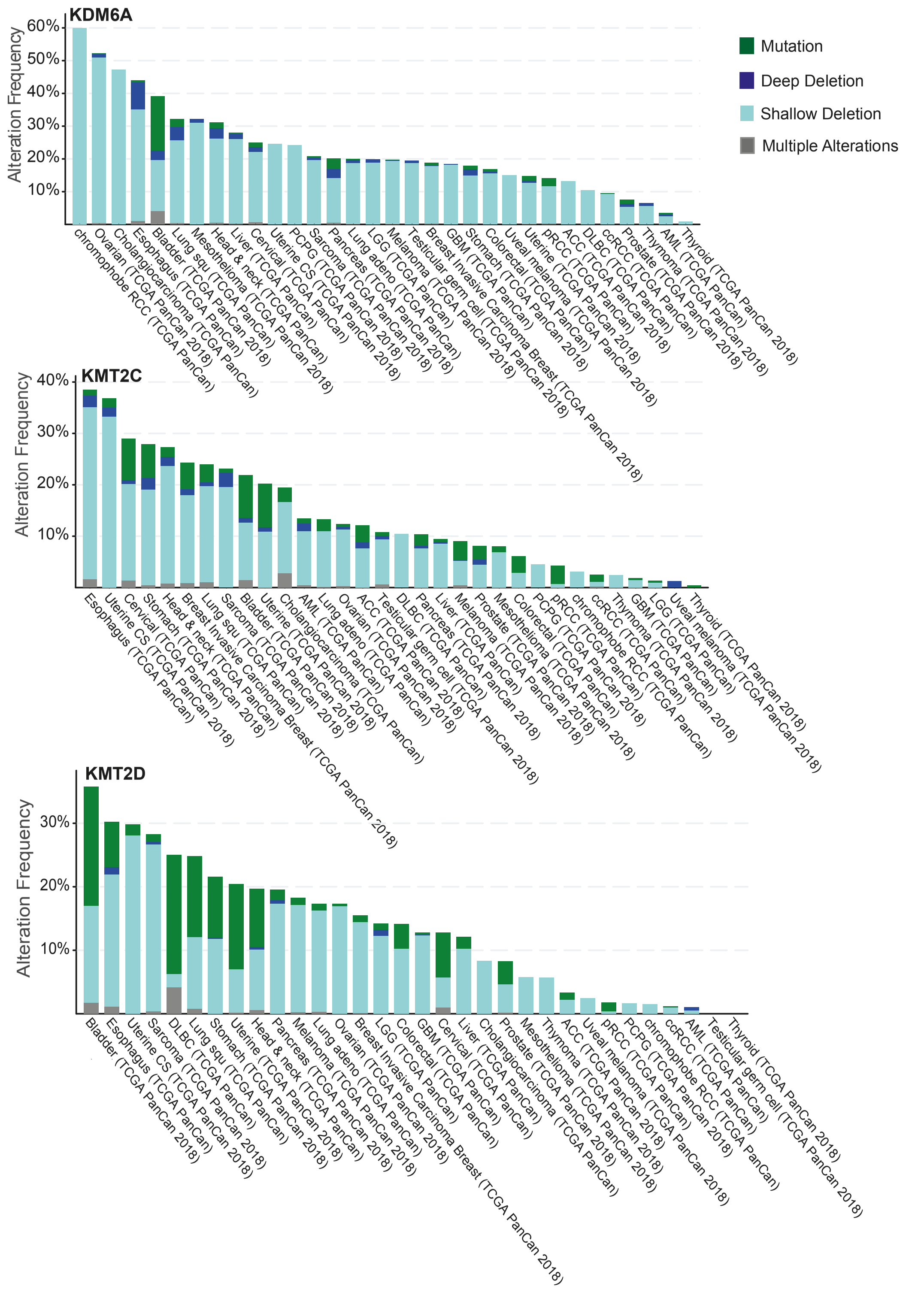

Suppl. Fig 2 Frequencies of Kdm6a, Kmt2c and Kmt2d alteration in different cancers as illustrated by cBioportal.

Supplemental material BMJ Publishing Group Limited (BMJ) disclaims all liability and responsibility arising from any reliance placed on this supplemental material which has been supplied by the author(s) Gut

Supplementary Figure 3

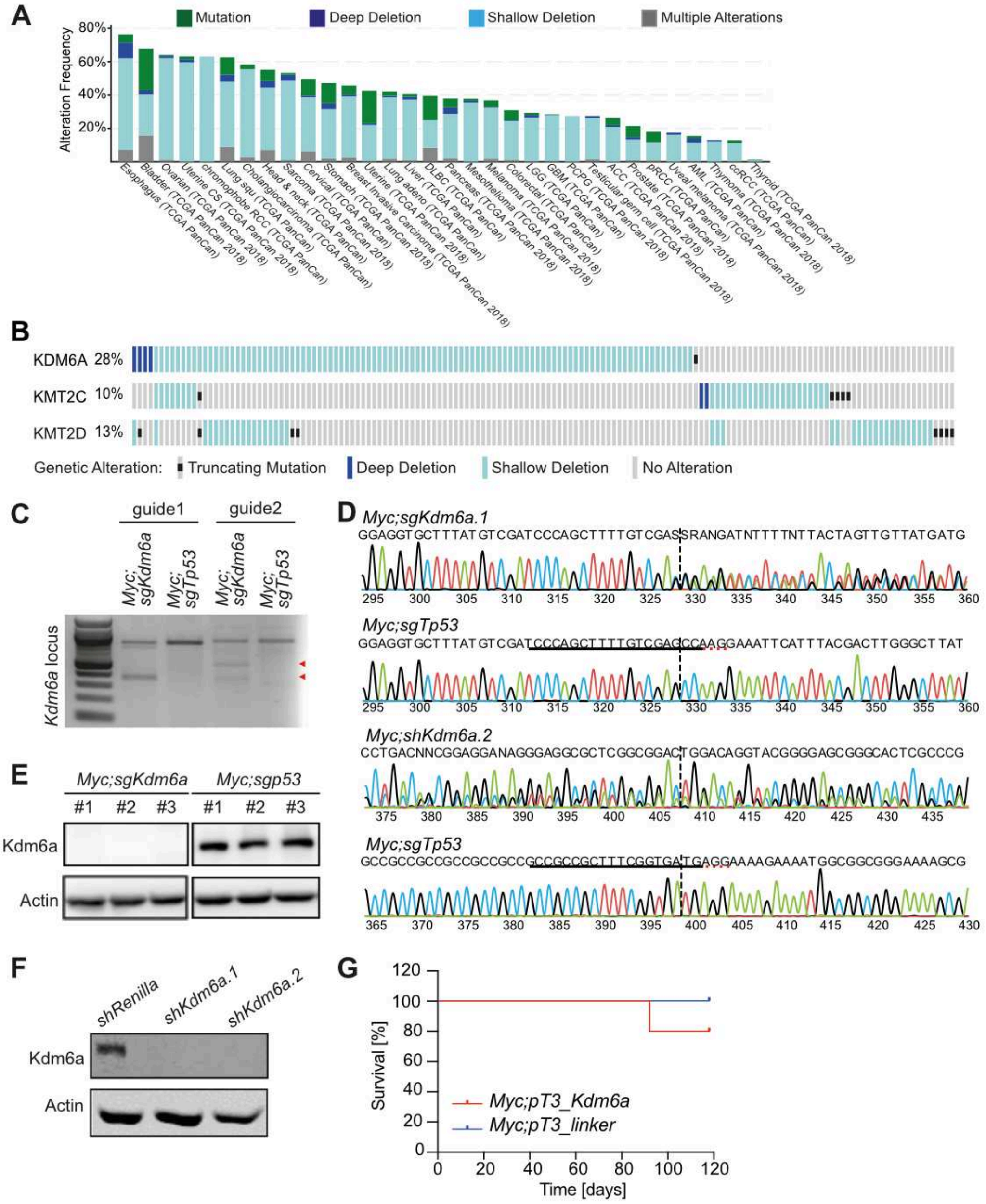

Suppl. Fig 3 CRISPR/Cas9-mediated editing and RNA interference-mediated suppression of Kdm6a. (A) Alteration frequencies of MLL3/MLL4 COMPASS-like complex across different cancer types in publicly available TCGA data sets. **(B)** OncoPrints displaying different deletions and mutations of MLL3/MLL4 COMPASS-like complex in publicly available TCGA data sets of sequenced HCCs. **(C)** T7 endonuclease assay of Kdm6a and Tp53 CRISPR cleavage sites. Cleaved bands are indicated by arrowheads. **(D)** Sanger sequencing of the CRISPR targeted *Kdm6a* locus. Dashed lines indicate the cutting site. **(E)** Immunoblot analyses for Kdm6a in tumor derived cell lines from Myc;sgTp53 and Myc;sgKdm6a; representative result of n = 3. **(F)** Immunoblot analyses of *Kdm6a* knockdown performed with two different shRNAs; representative result of n = 3. **(G)** Survival of Kdm6a overexpression mice with c-myc background (Myc;pT3_Kdm6a, red lines; n = 5) and linker (Myc;pT3_linker, blue line; n = 5) as control.

Supplementary Figure 4

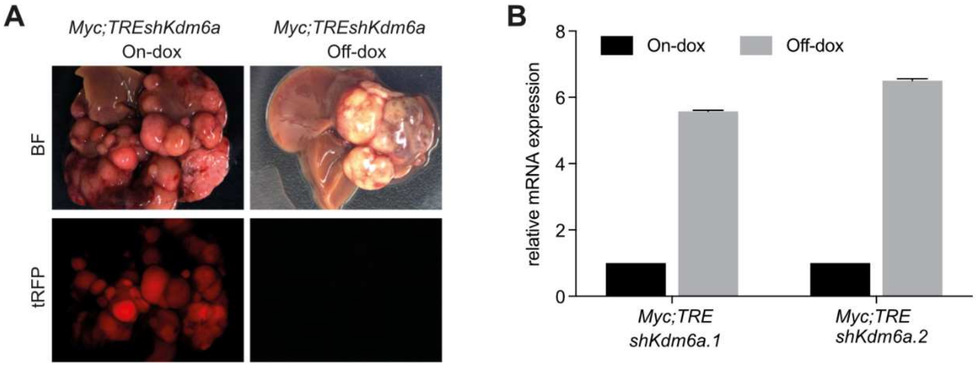

Suppl. Fig 4 Endogenous Kdm6a re-expression restrains tumor progression *in vivo*. (A) Tumor nodules observed in Myc;TREshKdm6a mice express shRNA-linked tRFP; Representative picture of n=6 for each group. **(B)** Quantitative PCR for Kdm6a expression in Myc;TREshKdm6a cell lines in the presence or absence of doxycycline. Representative results of n = 3 independent experiments.

Supplementary Figure 5

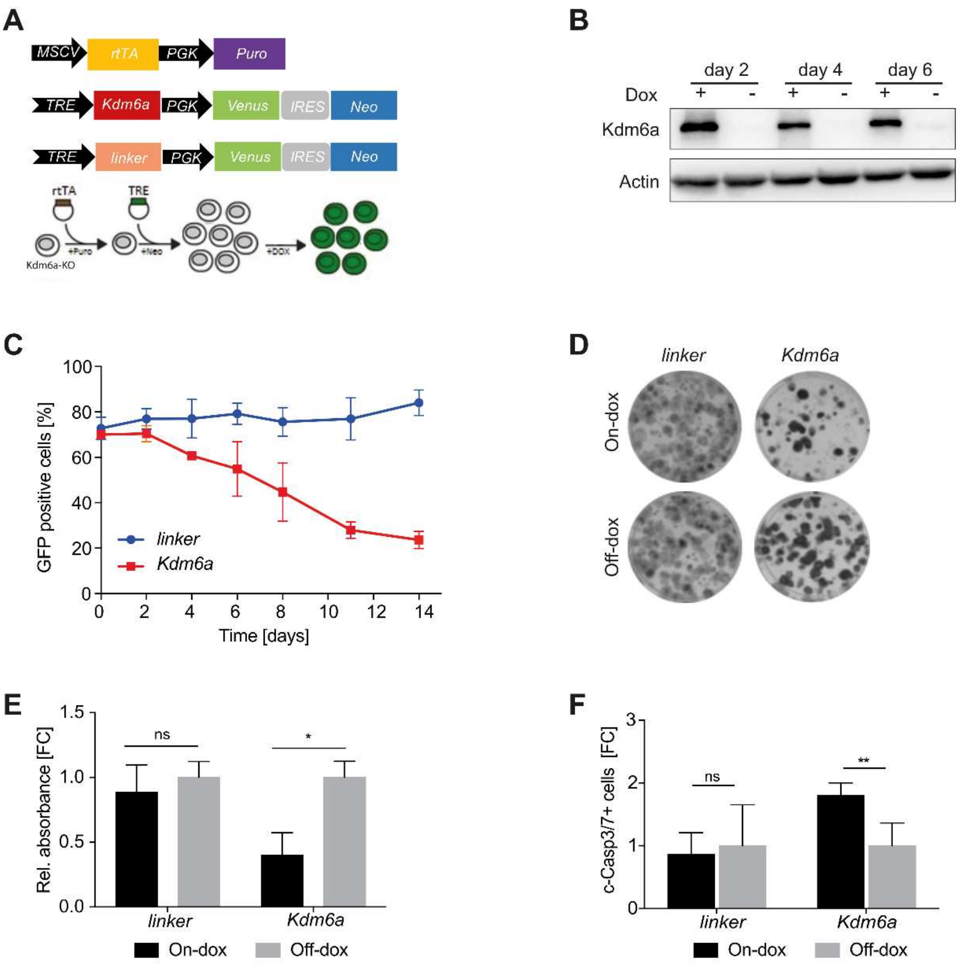

Suppl. Fig 5 Kdm6a overexpression causes apoptosis in Kdm6a deficient cells. (A) Top, vector constructs expressing rtTA and doxycycline-inducible vectors to overexpress either Kdm6a or linker (control) which is linked to GFP expression. Bottom, schematic workflow to generate rescue cell lines in Myc;sgKdm6a cell lines. **(B)** Immunoblot analyses of Kdm6a overexpression in Myc;sgKdm6a cell line with or without doxycycline. Actin was used as loading control. Representative result of n = 3. **(C)** Time course competition assay of Myc;sgKdm6a with either Kdm6a overexpression (red line) or linker as control (blue line). Error bars represent mean ± SD; n = 3. **(D)** Colony formation assay of Myc;sgKdm6a cell lines in the presence (Off-dox) or absence (On-dox) of Kdm6a for 10 days. Representative results of n = 3. **(E)** Colorimetric quantification of colony formation assay. Values are mean ± SD; n = 3. Unpaired t-test, *p-value = 0.0143. **(F)** Active caspase-3/7 labeling in the indicated cell lines grown for 6 days with and without doxycycline. Error bars represent mean ± SD; n = 3. Unpaired t-test, **p-value < 0.0094. FC = fold change.

Supplementary Figure 6

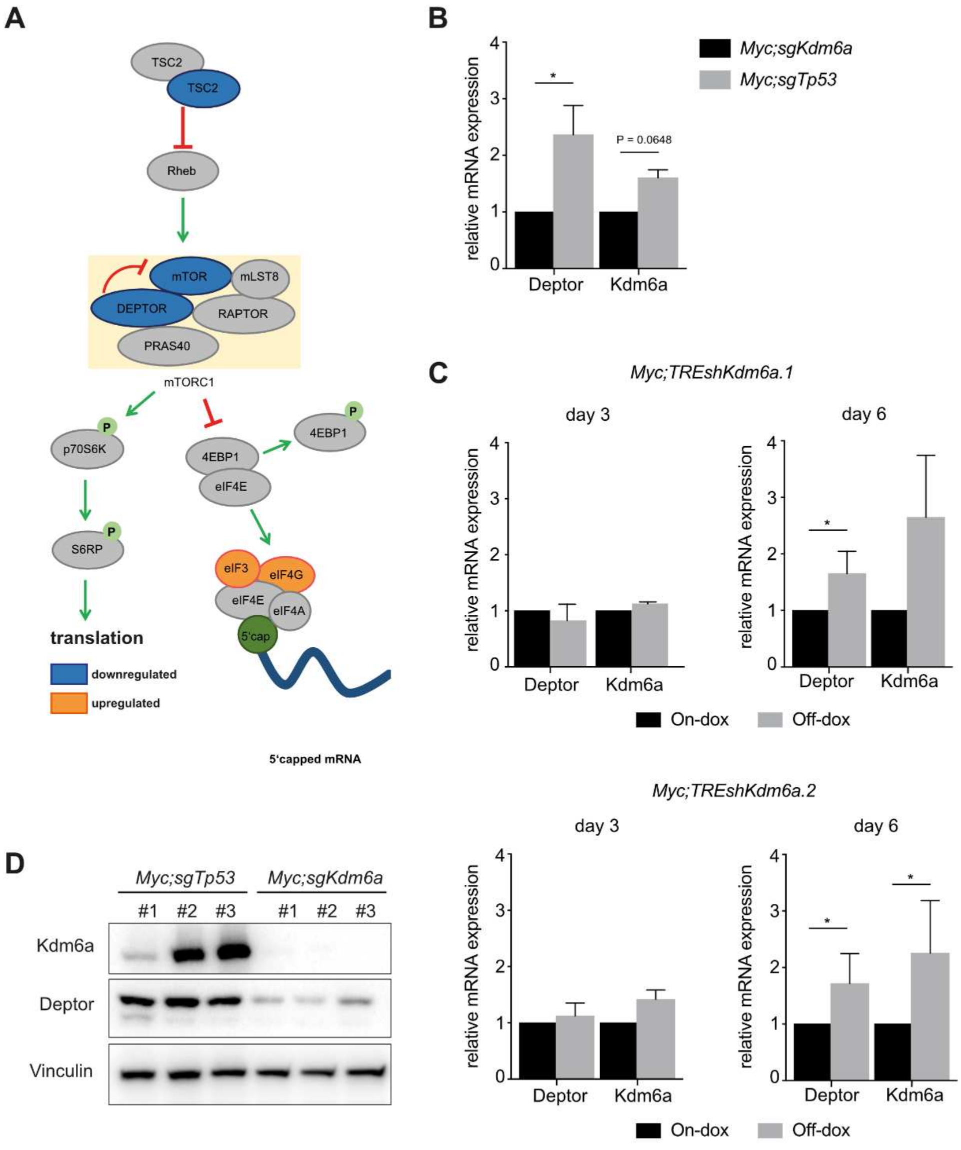

Suppl. Fig 6 Kdm6a dictates the transcriptional levels of Deptor. (A) Schematic display of mTORC1 signaling. **(B)** mRNA expression of *Deptor* and *Kdm6a* in Myc;sgKdm6a and Myc;sgTp53 cell lines, n = 2. Error bars represent mean ± SD. Unpaired t-test, *p-value = 0.0265. **(C)** mRNA expression of *Deptor* and *Kdm6a* in both Myc;TREshKdm6a cell lines with and without doxycycline on day 3 and day 6, n = 3. Error bars represent mean ± SD. Paired t-test, *p-value > 0.044. **(D)** Immunoblot of Deptor in three independent Myc;sgKdm6a and Myc;sgTp53 cell lines. Representative result of n = 3. E

Supplementary Figure 7

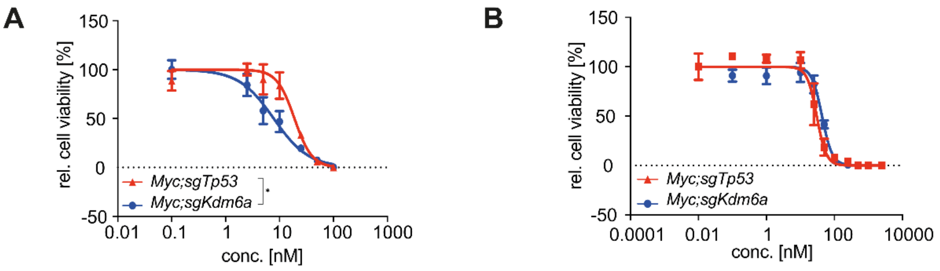

Suppl. Fig 7 Kmd6a-deficient cells are more vulnerable to mTOR inhibition. Dose response curve of **(A)** Torin-1 and **(B)** Dactolisib in Myc;sgTp53 and Myc;sgKdm6a cells as analyzed by CellTiter-Blue in the presence (red line) or absence (blue line) of Kdm6a. Dose response curves are representative results of n = 3 independent experiments. Error bars represent mean ± SD. Differences between logIC50 values were determined with unpaired t-test, *p-value = 0.001

Supplementary Figure 8

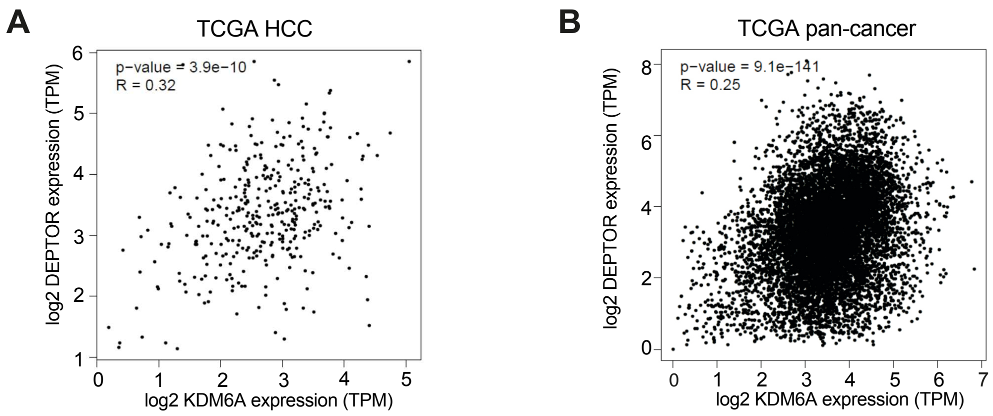

Suppl. Fig 8 Correlation between KDM6A and DEPTOR from publicly available HCC (A) and pan-cancer (B) data.

Supplementary Table 1. Clinicial and pathology data for HCC patients (cohort 1)

Sex	Survival (months)	Etiology	Tumor size	KDM6A	Deptor	KDM6A	Deptor	p-S6RP
				mRNA		IHC		
M	72	HCV	> 3cm	0.202	0.412	High	High	High
M	60	HCV	> 3cm	0.11	0.135	Low	Low	High
M	45	HBV	> 3cm	0.129	0.089	Low	Low	High
F	88	HBV	< 3cm	0.345	0.426	High	High	Low
M	95	Ethanol	< 3 cm	0.212	0.328	High	High	Low
M	86	HBV	> 3cm	0.278	0.212	High	High	Low
F	52.9	HBV	> 3cm	0.086	0.039	Low	Low	Low
M	43.1	HCV	> 3cm	0.245	0.402	High	High	High
F	52.6	HBV	> 3cm	0.314	0.416	High	High	Low
F	42	Ethanol	> 3cm	0.318	0.428	High	High	Low
M	48.2	HBV	< 3 cm	0.284	0.402	High	High	Low
M	48.6	HCV	> 3cm	0.256	0.464	High	High	Low
M	39	HCV	> 3cm	0.31	0.415	High	High	Low
M	38.3	HBV	< 3 cm	0.415	0.422	High	High	High
M	80.9	Ethanol	> 3cm	0.075	0.118	Low	Low	Low
M	88.3	Ethanol	> 3cm	0.054	0.092	Low	Low	High
M	50.8	HBV	< 3 cm	0.124	0.272	Low	High	Low
M	49.7	HCV	< 3 cm	0.126	0.076	Low	Low	High
M	43.9	HBV	> 3cm	0.338	0.248	High	High	Low
F	62	Wilson's disease	> 3cm	0.210	0.439	High	High	Low
M	38.2	HBV	> 3cm	0.086	0.105	Low	Low	High
M	36	HCV	< 3 cm	0.324	0.234	High	High	High
M	42	HCV	> 3cm	0.291	0.312	High	High	Low
M	48	HBV	> 3cm	0.312	0.358	High	High	High
F	62	HBV	> 3cm	0.228	0.235	High	High	Low
M	59	HBV	< 3 cm	0.354	0.262	High	High	Low
M	65	HCV	> 3cm	0.128	0.348	High	High	Low
F	80	HBV	> 3cm	0.052	0.078	Low	Low	High
M	44	HCV	< 3 cm	0.314	0.106	High	Low	Low
F	56.4	NA	> 3cm	0.035	0.114	Low	Low	High
F	86	HBV	> 3cm	0.102	0.05	Low	Low	High
F	78	HCV	< 3 cm	0.019	0.122	Low	Low	High
M	72.4	HBV	> 3cm	0.506	0.624	High	High	Low
M	42.2	HCV	> 3cm	0.122	0.448	Low	High	Low
F	38	HBV	< 3 cm	0.434	0.445	High	High	High
M	45	HCV	< 3 cm	0.074	0.125	Low	Low	High
M	72	NA	> 3cm	0.04	0.022	Low	Low	High
M	58	NA	> 3cm	0.286	0.348	High	High	High
M	26	HCV	> 3cm	0.512	0.338	High	High	Low
M	32.5	HBV	< 3 cm	0.108	0.320	Low	High	Low

F	6	HBV	< 3 cm	0.578	0.386	High	High	Low
F	25	HBV	> 3cm	0.422	0.48	High	High	Low
M	34	HCV	< 3 cm	0.312	0.78	High	High	Low
M	10.4	Ethanol	> 3cm	0.212	0.088	High	Low	High
M	2	Ethanol	> 3cm	0.386	0.84	High	High	Low
M	9.5	HCV	> 3cm	0.142	0.115	Low	Low	High
M	5	HBV	> 3cm	0.102	0.55	Low	High	Low
F	15.2	HBV	< 3 cm	0.48	0.108	High	Low	High
M	16.2	Ethanol	> 3cm	0.376	0.65	High	High	Low
M	11.8	HCV	> 3cm	0.312	0.96	High	High	Low
F	1.2	HCV	> 3cm	0.561	0.58	High	High	High
M	16	HCV	> 3cm	0.442	0.62	High	High	Low
M	1.4	HBV	> 3cm	0.356	0.54	High	High	Low
M	5	HBV	> 3cm	0.536	0.76	High	High	Low
M	18.9	HBV	> 3cm	0.408	0.58	High	High	Low
F	28	HCV	> 3cm	0.274	0.66	High	High	Low
F	11.8	Ethanol	> 3cm	0.405	0.058	High	Low	Low
M	10.4	HBV	> 3cm	0.612	0.76	High	High	Low
M	18	Ethanol	> 3cm	0.568	0.44	High	High	High
M	23.2	HBV	< 3 cm	0.345	0.55	High	High	High
M	28	HCV	< 3 cm	0.408	0.212	High	High	Low
M	20.4	HCV	< 3 cm	0.585	0.096	High	Low	Low
F	12.2	HBV	> 3cm	0.292	0.416	High	High	High
F	18	HBV	< 3 cm	0.515	0.626	High	High	High
M	14	Ethanol	> 3cm	0.089	0.112	Low	Low	High
M	22	HCV	> 3cm	0.644	0.289	High	High	Low
M	24.2	HCV	> 3cm	0.125	0.068	Low	Low	High
F	18	Ethanol	< 3 cm	0.185	0.215	High	High	High
M	30	HBV	> 3cm	0.296	0.472	High	High	Low
F	25.4	Ethanol	> 3cm	0.385	0.285	High	High	High
M	22.2	HBV	< 3 cm	0.276	0.516	High	High	Low
M	16	HCV	> 3cm	0.488	0.478	High	High	High
M	10	HCV	> 3cm	0.064	0.102	Low	Low	High
M	8.2	Ethanol	< 3 cm	0.046	0.091	Low	Low	High
M	26.2	NA	< 3 cm	0.285	0.412	High	High	Low
F	18.2	NA	> 3cm	0.346	0.614	High	High	Low

Supplementary Table 2. Clinicial and pathology data for HCC patients (cohort 2)

Internal identifier	Diagnosis	pT	pN	M	Grade	Kdm6a Score
E/2004/028304	Dysplastic Nodule	0	0	0	0	0
E/2006/007886	HCC	1	x	x	G2	0
E/2007/014308	HCC	3b	x	x	G2	0
E/2008/029178	HCC	1	0	x	G2	0
E/2007/012137	HCC	1	x	x	G2	0
E/2007/034388	HCC	2	x	x	G2	0
E/2010/017603	HCC	2	x		G2	0
E/2007/041191	HCC	2	0	x	G2	0
E/2006/000447	HCC	3	0	x	G2	0
E/2004/017009	HCC	3	x	x	G2	0
E/2004/018421	HCC	2	x	x	G2	0
E/2009/030482	HCC	3	x	x	G2	0
E/1998/042551	HCC	3	x	x	G2	0
E/2011/021776	HCC	1	x		G2	0
E/2004/022839	HCC	2	0	x	G2	0
E/2004/001725	HCC	1	x	x	G2	0
E/2011/000105	HCC	2	x		G4	0
E/2004/028025	HCC	2	0	x	G2	0
E/2009/035176	HCC	3	x	x	G2	0
E/2007/030051	HCC	3	x	x	G2	0
E/1996/028145	HCC	3	x	x	G2	0
E/1998/015362	HCC	4	x	x	G2	0
E/1995/050173	HCC	2				0
E/2008/009763	HCC	x	0	x	G2	0
E/2008/006069	HCC	3	x	x	G3	0
E/2009/025984	HCC	2	x	x	G2	0
E/2008/009941	HCC	2	0	x	G2	0
E/2009/011339	HCC	2	x	x	G2	0
E/2004/004436	HCC	2	x		G1	0
E/2009/028054	HCC	2	x	1	G2	0
E/2009/029835	HCC	1	x	x	G2	0
E/2009/018621	HCC	3	x	x	G2	0
E/2007/026344	HCC	3	0	x	G2	0
E/2006/011051	HCC	2	x	x	G3	0
E/2006/019880	HCC	1	x	x	G2	0
E/2008/002589	HCC	3	0	x	G2	0
E/2010/024944	HCC	3a	x		G1	0
E/2007/011423	HCC	1	x	x	G2	0
E/2007/021831	HCC	3	x	x	G3	0
E/2009/000198	HCC	2	0	x	G1	0
E/2009/035176	HCC	3	x	x	G2	0

E/2010/014817	HCC	2	x	x	G3	0
E/2010/012277	HCC	2	0	x	G2	0
E/2010/014498	HCC	1	0		G2	0
E/2004/022161	HCC	3	0	x	G3	0
E/2010/011229	HCC	2	x	x	G3	0
E/2010/004233	HCC	3a	x	x	G2	0
E/2008/020958	HCC	2	x	x	G3	0
E/2006/000590	HCC	3m	0	x	G2	0
E/2009/028419	HCC	2	0	x	G2	0
E/2009/025448	HCC	2	x	x	G2	0
E/2010/001867	HCC	2	x	x	G2	0
E/2009/024478	HCC	3	x	x	G2	0
E/2009/020906	HCC	2	x	x	G3	0
E/2009/009219	HCC	2	x	x	G2	0
E/2010/025847	HCC	2	0		G2	0
E/2010/025898	HCC	1	x		G2	0
E/2008/015562	HCC	2	0	x	G2	0
E/1997/012198	HCC	2	x	x	G2	0
E/1996/008217	HCC	1	0	x	G2	0
E/2007/029008	HCC	2	x	x	G2	0
E/2007/030803	HCC	2	x	x	G2	0
E/2007/013409	HCC	1	x	x	G2	0
E/2008/018813	HCC	2	0	x	G2	0
E/2009/040780	HCC	3	x	x	G2	0
E/2009/040171	HCC	2	x	x	G2	0
E/2010/031126	HCC	2	0		G3	0
E/2010/035713	HCC	2	x	x	G2	0
E/2010/043247	HCC	2	x	x	G3	0
E/2010/045216	HCC	1	x	x	G2	0
E/2007/044946	HCC	2	x	x	G2	0
E/2007/032756	HCC	3	x	x	G2	0
E/2011/013695	HCC	1	x	x	G1	0
E/2011/012581	HCC	2	x	x	G2	0
E/2011/005176	HCC	2	0		G2	0
E/2005/041203	HCC	1	x	x	G2	0
E/2005/041203	Dysplastic Nodule	0	0	0	0	0
E/2007/030453	HCC	2	x	x	G2	0
E/2007/020487	HCC	2	x	x	G3	0
E/2007/010212	HCC	1	x	x	G2	0
E/2009/012459	HCC	2	x	x	G2	0
E/2009/042916	HCC	2	x	x	G1	0
E/1998/003592	HCC	2	0	x	G2	0
E/1996/024632	HCC	2	x	x	G2	0

E/1997/005823	HCC	1			G1	0
E/1998/018449	HCC	4	x	x	G2	0
E/2005/017677	HCC	2	x	1	G3	0
E/2005/033465	HCC	1	x	x	G1	0
E/2009/025693	HCC	2	x	x	G2	0
E/2005/008055	HCC	3	x	x	G3-4	0
E/2004/029790	HCC	1	0	x	G2	0
E/2003/017887	HCC	3	x	x	G2	0
E/2003/018009	HCC	3	x	x	G2	0
E/2011/020620	HCC	2	x	x	G2	0
E/2001/040755	HCC	2	x	x	G1	0
E/1999/020380	HCC	2	x	x	G2	0
E/2005/003715	HCC	2	0	x	G2	0
E/2003/012759	HCC	1	x	x	G4	0
E/1997/001948	HCC	2	x	x	G2	0
E/1999/007528	HCC	1	x	x	G2	0
E/1996/031312	HCC	1	0	x	G2	0
E/1997/000552	HCC	4	0	x	G2	0
E/2003/033612	HCC	3	x		G2	0
E/2002/044818	HCC	1	x	x	G2	0
E/1996/011606	HCC	4	0	x	G2	0
E/2002/003448	HCC	4	x	x	G2	0
E/2005/034265	HCC	2	x	x	G1-2	0
E/2005/012216	HCC	1	0	x	G2	0
E/2005/037923	HCC	3	x	x	G1-2	0
E/2005/025841	HCC					0
E/1999/022828	HCC	3	x	x	G2-3	0
E/1998/024408	HCC	4	0	x	G2-3	0
E/1998/002519	HCC	4	x	x	G3-4	0
E/1998/042449	HCC	2	0	x	G2	0
E/1998/003592	HCC	2	0	x	G2	0
E/1998/018449	HCC	4	x	x	G2	0
E/1999/002809	HCC	1			G2	0
E/2009/028054	HCC	2	x	1	G2	0
E/2004/024468	HCC	1	0	x	G2	0
E/2004/025562	HCC	2	x	x	G2	0
E/1996/011606	HCC	4	0	x	G2	0
E/1991/048989	HCC					0
E/2004/018421	HCC	2	x	x	G2	0
E/1990/017144	HCC					0
E/2006/012756	HCC	1	x	x	G2	0
E/1991/011475	HCC					0
E/2001/038508	HCC	2			G2	0

E/1992/044329	HCC					0
E/2003/021642	HCC	1	x		G1	0
E/1991/041134	HCC					0
E/2001/014497	HCC				G3	0
E/2005/003199	HCC	2	x	x	G2	0
E/1999/036052	HCC	2	x	x	G2	0
E/2001/038508	HCC	2			G2	0
E/2001/024904	HCC	3	x	x	G2	0
E/2000/012801	HCC	4	x	x	G2	0
E/1992/044329	HCC					0
E/1989/047976	HCC					0
E/1992/049560	HCC					0
E/1993/062954	HCC					0
E/1993/034711	HCC					0
E/1992/033976	HCC					0
E/1986/048762	HCC					0
E/1989/021987	HCC					0
E/1998/024408	HCC	4	0	x	G2-3	0
E/1991/031949	HCC					0
E/1992/005817	HCC					0
E/2000/031217	HCC	4	x	x		0
K/1984/033946	HCC					0
K/1999/016913	HCC					0
K/1999/016913	HCC					0
K/1994/017900	HCC				G1	0
K/1994/011127	HCC					0
K/1983/028808	HCC				G2	0
K/1989/012158	HCC					0
K/1991/021219	HCC				G2	0
K/1991/021219	HCC				G2	0
K/2003/017553	HCC				G2	0
K/1998/021957	HCC				G2	0
K/????/0BL456	HCC					0
K/2003/001161	HCC					0
K/2001/016619	HCC				G3	0
K/2003/008445	HCC				G1	0
K/2001/024085	HCC				G1	0
K/2001/024085	HCC				G1	0
K/2000/024181	HCC				G2	0
K/2002/026419	HCC				G3	0
K/1999/017688	HCC				G2	0
K/2001/014588	HCC				G1	0
K/2003/004020	HCC					0

K/2001/008049	HCC					0
K/2003/008645	HCC					0
K/2003/008645	HCC					0
K/2003/017553	HCC					0
K/????/014195	HCC					0
E/2009/035364	HCC	3	x	x	G2	0
E/2009/009219	Cirrhosis	0	0	0	0	0
E/1997/026558	HCC	2	x	x	G3	0
E/2008/042858	HCC	1	x	x	G3	0
E/2004/003991	Liver	0	0	0	0	0
E/2005/009944	Cirrhosis	0	0	0	0	0
E/2004/029790	Liver	0	0	0	0	0
E/2004/002479	Dysplastic Nodule	0	0	0	0	0
E/2001/004766	Cirrhosis	0	0	0	0	0
E/2009/037136	Cirrhosis	0	0	0	0	0
E/2010/014817	Cirrhosis	0	0	0	0	0
E/2010/004233	Cirrhosis	0	0	0	0	0
E/2010/017174	HCC	1	x		G2	0
E/2011/005176	Cirrhosis	0	0	0	0	0
E/1999/008866	Liver	0	0	0	0	0
E/2009/000196	Cirrhosis	0	0	0	0	0
E/2004/028304	Cirrhosis	0	0	0	0	0
E/2003/034246	Cirrhosis	0	0	0	0	0
E/2006/034961	Liver	0	0	0	0	0
E/2009/042679	Cirrhosis	0	0	0	0	0
E/2004/006851	Cirrhosis	0	0	0	0	0
E/2006/010461	Dysplastic Nodule	0	0	0	0	1
E/2004/028304	Dysplastic Nodule	0	0	0	0	1
E/1990/026199-200	Dysplastic Nodule	0	0	0	0	1
E/1991/011476	Dysplastic Nodule	0	0	0	0	1
K/2003/004020	Dysplastic Nodule	0	0	0	0	1
K/2003/004020	Dysplastic Nodule	0	0	0	0	1
K/2003/004020	Dysplastic Nodule	0	0	0	0	1
K/2003/004020	Dysplastic Nodule	0	0	0	0	1
E/2010/023237	HCC	1	0 (0/2)	x	G2	1
E/2008/027517	HCC					1
E/2007/022919	HCC	1	x	x	G2	1
E/2007/008140	HCC	2	x	x	G4	1
E/2002/043876	HCC	2	x		G1	1
E/2007/026955	HCC	2	0	x	G3	1
E/2003/005487	HCC	2	x	x	G2	1
E/2007/035264	HCC	3	x	x	G2	1
E/2004/017664	HCC	3	0	x	G2	1

E/2005/040894	HCC	2	0		G1	1
E/2003/000607	HCC	1	x	x	G2	1
E/2006/007119	HCC	1	x	0	G3	1
E/2005/041510	HCC	1	x	x	G1	1
E/2004/003991	HCC	2	0		G1	1
E/1995/050790	HCC	2	x	x	G1	1
E/2011/014660	HCC	1	x		G1	1
E/1997/000685	HCC	2	x	x	G2	1
E/2004/002479	HCC	1	0	x	G2	1
E/2001/032824	HCC	3	x		G2	1
E/2002/027472	HCC	3	x	x	G2	1
E/2005/040112	HCC	1	x	x	G3	1
E/2011/000782	HCC	1	0		G1	1
E/2011/000481	HCC	2	x		G3	1
E/2011/000209	HCC	1			G1	1
E/2001/024406	HCC	4	x	x	G2	1
E/2001/041821	HCC					1
E/2004/003991	Cirrhosis	0	0	0	0	1
E/2005/030013	HCC	4	x	x	G3	1
E/2009/044473	HCC	1	0	x	G1	1
E/2009/033157	HCC	1	x	x	G1	1
E/1997/017638	HCC	2	x	x	G1	1
E/1998/021651	HCC	2			G1	1
E/2009/030380	HCC	3	0	x	G2	1
E/2008/004571	HCC	2	x	x	G3	1
E/2008/007151	HCC	1	x	x	G1	1
E/2009/018573	HCC	2	x	x	G2	1
E/2004/007196	HCC	2	x	x	G2	1
E/2009/014727	HCC	2	x	x	G3	1
E/2007/026167	HCC	3	0	x	G2	1
E/2006/010995	HCC	2	x	x	G3	1
E/2006/015021	HCC	1	x	x	G1	1
E/2008/003977	HCC	3	x	x	G3	1
E/1997/005823	HCC	1	x	x	G1	1
E/2007/039285	HCC	2	x	x	G2	1
E/2007/041922	HCC	2	0	x	G3	1
E/2010/001721	HCC	1	x	x	G2	1
E/2009/037136	HCC	2	x	x	G1	1
E/2008/045418	HCC	2	x	x	G2	1
E/2010/014879	HCC	1	x	x	G2	1
E/2004/022542	HCC	2	x	x	G2	1
E/2004/021502	HCC	1	0	x		1
E/2010/004099	HCC	2	x	x	G2	1

E/2010/014033	HCC	1	x	x	G2	1
E/2006/007405	HCC	2	x	x	G2	1
E/2008/043988	HCC	2	x	x	G2	1
E/2009/033316	HCC	1	x	x	G2	1
E/2009/024478	HCC	3	x	x	G2	1
E/1996/006473	HCC	4			G2	1
E/2010/002483	HCC	1	0		G2	1
E/2009/040174	HCC	2	x	x	G2	1
E/2009/036423	HCC	2			G2	1
E/2009/025981	HCC	2	x	x	G2	1
E/2009/010532	HCC	1	x	x	G2	1
E/2007/043261	HCC	2	x	x	G2	1
E/2007/007337	HCC	2m	x	x	G2	1
E/2009/002634	HCC	4	x	x	G3	1
E/2008/038544	HCC	4	x		G3	1
E/2006/032130	HCC	2	0	x	G2	1
E/2010/032931	HCC	2	x	x	G4	1
E/2010/041694	HCC	3a	0	x	G2	1
E/2010/045776	HCC	2	x	x	G3	1
E/2010/043646	HCC	2	x	x	G2	1
E/2011/011212	HCC	3b	x	x	G3	1
E/2011/012974	HCC	1	x		G1	1
E/2011/006575	HCC	2	0	x	G1-G3	1
E/2011/003385	HCC	1	x	x	G2	1
E/2009/000196	HCC	1	x	x	G4	1
E/2005/038343	HCC	1	x	x	G1	1
E/2007/020487	HCC	2	x	x	G3	1
E/2004/020608	HCC	2m	x	x	G2	1
E/2001/010465	HCC	2	x	x	G4	1
E/1995/048004	HCC	2	x	x	G2-3	1
E/2005/021847	HCC	x	x	x	G2	1
E/2004/028304	HCC	2	0	x	G2	1
E/2009/020754	HCC	2	x	x	G2	1
E/2008/029361	HCC	2	0	x	G2	1
E/2004/032822	HCC	2	0	x	G1	1
E/2002/026552	HCC	1	0	0	G2	1
E/2003/009358	HCC	1	x	x	G1	1
E/2011/021154	HCC	1	x		G2	1
E/2011/019779	HCC				G2	1
E/2011/018062	HCC	1	x	x	G2	1
E/2011/019063	HCC	2	x	x	G2	1
E/2010/031062	HCC	1	x	x	G2	1
E/2001/004766	HCC	3	x	x		1

E/2002/003822	HCC					1
E/2004/037769	HCC	1	0	x	G3	1
E/1997/009588	HCC	2	x	x	G1	1
E/2004/028139	HCC	2	x	x	G2	1
E/2004/002479	HCC	1	0	x	G1	1
E/2000/008312	HCC	2	x	x	G1	1
E/1997/028938	HCC	3				1
E/2005/001443	HCC	1	x	x	G2	1
E/2005/015088	HCC	2	x	x	G2	1
E/2003/000858	HCC	1	x		G1	1
E/2002/022770	HCC	2				1
E/2003/034246	HCC	2	0	x	G2	1
E/2002/022417	HCC	2	x	x	G2	1
E/2009/030716	HCC	2	x	x	G3	1
E/2007/026775	HCC	1	x	x	G2	1
E/2001/005936	HCC	1	x	x		1
E/2010/024944	HCC	3a	0		G1	1
E/1997/026558	HCC	2	x	x	G3	1
E/2005/037157	HCC	1			G3	1
E/2005/039994	HCC	2	x	x	G2	1
E/2005/040894	HCC	2m	0		G1-2	1
E/1998/042551	HCC	3	x	x	G2	1
E/1997/034035	HCC	3	x	x	G1	1
E/1997/004270	HCC	2	0	x	G2	1
E/1996/005766	HCC	4	0	x	G2	1
E/1997/020433	HCC	3	x	x	G2	1
E/1999/006733	HCC	2	x	x	G2	1
E/1997/012198	HCC	2	x	x	G2	1
E/2005/024190	HCC	2	x	x	G1	1
E/2000/036797	HCC	3	x	x	G3	1
E/2006/013613	HCC	2m	x	x	G2	1
E/2004/000056	HCC	2			G3	1
E/1997/001792	HCC	2	x	x	G2-3	1
E/1999/026565	HCC	2	x	x	G2-3	1
E/1996/006771	HCC	2	x	x	G2	1
E/2006/013613	HCC	2m	x	x	G2	1
E/2006/013613	HCC	2m	x	x	G2	1
E/2006/013613	HCC	2m	x	x	G2	1
E/2009/045549	HCC	2	0	x	G2	1
E/1996/028145	HCC	3	x	x	G2	1
E/2001/004766	HCC	3	x	x		1
E/2004/006851	HCC	1			G2	1
E/2005/022026	HCC	2			G2	1

E/2004/037769	HCC	1	0	x	G3	1
E/1991/039754	HCC					1
E/1991/009304	HCC					1
E/1992/005818	HCC					1
E/1991/039753-4	HCC					1
E/1991/039753-5	HCC					1
E/2004/031688	HCC	1	0	x		1
E/2003/001779	HCC	4	x	x	G2	1
E/1993/034711	HCC					1
E/1988/041821	HCC					1
E/1994/021334	HCC					1
E/1995/027279	HCC					1
E/1987/026364	HCC					1
E/1991/037828	HCC					1
E/1994/047444	HCC					1
E/2010/025157	HCC	1	x	x	G2	1
E/2002/036027	HCC	3	0	x	G1	1
E/2002/042302	HCC	4	0	x	G1	1
E/2002/018622	HCC	4	x	x	G2	1
E/1998/015362	HCC	4	x	x	G2	1
E/2005/002871	HCC	2	x	x	G2	1
E/2005/002871	HCC	2	x	x	G2	1
E/2005/001757	HCC	1	x	x	G2	1
E/2003/014116	HCC	3	x	x		1
E/1998/037054	HCC					1
E/2001/002474	HCC	4	x	x	G1	1
E/2001/020437	HCC	2	x	x		1
E/2001/017599	HCC	1	x	x	G1	1
E/2001/016345	HCC	2	x	x		1
E/1992/006774	HCC					1
E/1992/010240	HCC					1
E/1992/019414	HCC					1
E/1989/021229	HCC					1
E/1989/048366	HCC					1
E/1994/043436	HCC					1
E/1994/021334	HCC					1
E/1994/059493	HCC					1
E/1994/059493	Cirrhosis	0	0	0	0	1
E/1992/058644	HCC					1
E/1989/017053	HCC					1
E/1992/027251	HCC					1
E/1995/027299	HCC					1
E/1989/041327	HCC					1

E/1991/020186	HCC					1
E/1992/021074	HCC					1
E/1992/050504	HCC					1
E/1991/009304	HCC					1
E/1991/033152	HCC					1
E/1995/025186	HCC					1
E/1991/041134	HCC					1
E/1990/051371	HCC					1
E/2005/012583	HCC	2	0	x	G2	1
K/1984/033973	HCC					1
K/1996/071738	HCC				G1	1
K/1985/035678	HCC				G2	1
K/1998/006621	HCC				G2	1
K/1987/012432	HCC				G2	1
K/1989/012158	HCC					1
K/1989/012158	HCC					1
K/1991/071146	HCC				G2	1
K/1987/004624	HCC					1
K/1995/008822	HCC				G3	1
K/2002/026419	HCC				G3	1
K/1998/019340	HCC				G2	1
K/1998/006621	HCC				G2	1
K/2002/024313	HCC				G1	1
K/1998/021957	HCC				G1	1
K/2002/026419	HCC				G2	1
K/2003/028245	HCC					1
K/1998/025561	HCC					1
K/2003/024747	HCC					1
K/2002/030710	HCC				G3	1
K/2002/030710	HCC				G4	1
K/2000/007461	HCC				G1-2	1
K/2003/008654	HCC				G1	1
K/2003/008445	HCC				G3	1
K/2003/009610	HCC				G1	1
K/2002/017690	HCC				G1	1
K/2002/017690	HCC				G1	1
K/2002/017690	HCC				G1	1
K/2002/024313	HCC				G2	1
K/2003/009610	HCC				G2	1
K/2002/031041	HCC					1
K/2002/030947	HCC					1
K/2002/030947	HCC					1
E/2005/040894	Cirrhosis	0	0	0	0	1

K/2000/007461	HCC				G1-2	1
K/2001/004502	HCC				G1-2	1
K/2000/019523	HCC					1
K/2002/018880	HCC					1
K/2002/018880	HCC					1
K/2000/019523	HCC					1
K/2003/001161	HCC					1
K/2003/001161	HCC					1
K/2003/004020	HCC					1
K/2001/008049	HCC					1
K/2003/008445	HCC					1
K/2003/009610	HCC					1
K/2003/009610	HCC					1
K/2003/017553	HCC					1
E/2008/003977	HCC	3	x	x	G3	1
E/2008/022550	HCC	2	0	x	G2	1
E/2004/025846	Dysplastic Nodule	0	0	0	0	1
E/1998/015362	Dysplastic Nodule	0	0	0	0	1
E/2002/032143	HCC	2	x	x	G1	1
E/1991/052469	Cirrhosis	0	0	0	0	1
E/2007/008140	Cirrhosis	0	0	0	0	1
E/2007/012137	Cirrhosis	0	0	0	0	1
E/2007/034388	Cirrhosis	0	0	0	0	1
E/2004/030407	Liver	0	0	0	0	1
E/2010/017603	Liver	0	0	0	0	1
E/2007/041191	Cirrhosis	0	0	0	0	1
E/2007/035264	Cirrhosis	0	0	0	0	1
E/2004/017664	Cirrhosis	0	0	0	0	1
E/2006/007119	Cirrhosis	0	0	0	0	1
E/2004/025846	HCC	1	x	x	G2	1
E/2004/025846	Cirrhosis	0	0	0	0	1
E/1996/011606	Cirrhosis	0	0	0	0	1
E/1998/018449	Liver	0	0	0	0	1
E/2005/001446	Liver	0	0	0	0	1
E/2004/017009	Cirrhosis	0	0	0	0	1
E/2004/032822	Liver	0	0	0	0	1
E/2004/028139	Cirrhosis	0	0	0	0	1
E/1996/028145	Cirrhosis	0	0	0	0	1
E/2011/014660	Liver	0	0	0	0	1
E/2011/016480	Liver	0	0	0	0	1
E/2011/017295	Cirrhosis	0	0	0	0	1
E/2007/026167	Liver	0	0	0	0	1
E/2004/035017	Cirrhosis	0	0	0	0	1

E/2001/032824	Cirrhosis	0	0	0	0	1
E/2004/020608	HCC	2	x	x	G2	1
E/2005/040112	Cirrhosis	0	0	0	0	1
E/2011/000782	Liver	0	0	0	0	1
E/2008/013199	Cirrhosis	0	0	0	0	1
E/2009/044075	Cirrhosis	0	0	0	0	1
E/1996/005766	Cirrhosis	0	0	0	0	1
E/1996/005033	Cirrhosis	0	0	0	0	1
E/2005/008055	Cirrhosis	0	0	0	0	1
E/2011/000105	Liver	0	0	0	0	1
E/2002/028552	Liver	0	0	0	0	1
E/2009/035176	Cirrhosis	0	0	0	0	1
E/2009/033157	Cirrhosis	0	0	0	0	1
E/1998/015362	Cirrhosis	0	0	0	0	1
E/2004/028025	Cirrhosis	0	0	0	0	1
E/2009/030380	Cirrhosis	0	0	0	0	1
E/2008/009763	Cirrhosis	0	0	0	0	1
E/2009/025984	Cirrhosis	0	0	0	0	1
E/2008/009941	Cirrhosis	0	0	0	0	1
E/2009/011339	Cirrhosis	0	0	0	0	1
E/2004/004436	Cirrhosis	0	0	0	0	1
E/2004/007196	Cirrhosis	0	0	0	0	1
E/2009/011339	Cirrhosis	0	0	0	0	1
E/2009/018621	Liver	0	0	0	0	1
E/2007/026344	Cirrhosis	0	0	0	0	1
E/2006/010995	Cirrhosis	0	0	0	0	1
E/2008/003977	Cirrhosis	0	0	0	0	1
E/2010/024944	Cirrhosis	0	0	0	0	1
E/2007/039285	Cirrhosis	0	0	0	0	1
E/2009/002258	Liver	0	0	0	0	1
E/2007/021831	Cirrhosis	0	0	0	0	1
E/2009/000198	Cirrhosis	0	0	0	0	1
E/2009/035176	Cirrhosis	0	0	0	0	1
E/1992/015858	Cirrhosis	0	0	0	0	1
E/2010/014879	Cirrhosis	0	0	0	0	1
E/2010/012277	Cirrhosis	0	0	0	0	1
E/2004/021502	Cirrhosis	0	0	0	0	1
E/2010/011229	Cirrhosis	0	0	0	0	1
E/2010/004099	Cirrhosis	0	0	0	0	1
E/2006/004144	Cirrhosis	0	0	0	0	1
E/2009/033316	Cirrhosis	0	0	0	0	1
E/2009/024478	Cirrhosis	0	0	0	0	1
E/2010/017174	Cirrhosis	0	0	0	0	1

E/2005/002492	Liver	0	0	0	0	1
E/2010/002483	Cirrhosis	0	0	0	0	1
E/2010/001867	Cirrhosis	0	0	0	0	1
E/2009/040174	Cirrhosis	0	0	0	0	1
E/2009/024478	Cirrhosis	0	0	0	0	1
E/2009/020375	Cirrhosis	0	0	0	0	1
E/2009/017473	HCC	2	x	x	G2	1
E/2009/020906	Cirrhosis	0	0	0	0	1
E/2009/036423	Cirrhosis	0	0	0	0	1
E/2010/025847	Liver	0	0	0	0	1
E/2009/025981	Cirrhosis	0	0	0	0	1
E/2004/019059	Liver	0	0	0	0	1
E/2004/017788	Liver	0	0	0	0	1
E/2004/010370	Liver	0	0	0	0	1
E/2004/018778	Liver	0	0	0	0	1
E/2004/019092	Liver	0	0	0	0	1
E/1996/008217	Cirrhosis	0	0	0	0	1
E/2008/042399	Cirrhosis	0	0	0	0	1
E/2006/032130	Cirrhosis	0	0	0	0	1
E/2009/035364	Cirrhosis	0	0	0	0	1
E/2009/040171	Cirrhosis	0	0	0	0	1
E/2010/031126	Liver	0	0	0	0	1
E/2010/032451	Cirrhosis	0	0	0	0	1
E/2010/033638	Cirrhosis	0	0	0	0	1
E/2010/036261	HCC	2b	x		G3	1
E/2010/036261	Liver	0	0	0	0	1
E/2010/038187	HCC	2	x		G2	1
E/2010/038187	Cirrhosis	0	0	0	0	1
E/2010/043247	Cirrhosis	0	0	0	0	1
E/2010/043247	Cirrhosis	0	0	0	0	1
E/2006/025887	HCC	2m	x	x	G3	1
E/2007/032756	Cirrhosis	0	0	0	0	1
E/2011/013695	Cirrhosis	0	0	0	0	1
E/2011/012989	Cirrhosis	0	0	0	0	1
E/2011/011212	Cirrhosis	0	0	0	0	1
E/2011/012974	Liver	0	0	0	0	1
E/2011/012978	Liver	0	0	0	0	1
E/2011/021154	Liver	0	0	0	0	1
E/2002/020190	Liver	0	0	0	0	1
E/2000/030911	Liver	0	0	0	0	1
E/2005/009676	Liver	0	0	0	0	1
E/2009/031122	HCC	2	x	x	G2	1
E/2005/041203	Cirrhosis	0	0	0	0	1

E/2007/030453	Cirrhosis	0	0	0	0	1
E/2007/020487	Cirrhosis	0	0	0	0	1
E/2004/017489	Cirrhosis	0	0	0	0	1
E/2004/017489	Dysplastic Nodule	0	0	0	0	1
E/1997/005823	Cirrhosis	0	0	0	0	1
E/1995/048004	Cirrhosis	0	0	0	0	1
E/2005/017677	Cirrhosis	0	0	0	0	1
E/2004/028304	Cirrhosis	0	0	0	0	1
E/2008/023254	Cirrhosis	0	0	0	0	1
E/2006/010461	Cirrhosis	0	0	0	0	1
E/2009/025693	Cirrhosis	0	0	0	0	1
E/2009/025693	Cirrhosis	0	0	0	0	1
E/2002/026552	Cirrhosis	0	0	0	0	1
E/2003/009358	Cirrhosis	0	0	0	0	1
E/2011/018062	Cirrhosis	0	0	0	0	1
E/2011/019063	Liver	0	0	0	0	1
E/2011/018511	Cirrhosis	0	0	0	0	1
E/2001/004766	Cirrhosis	0	0	0	0	1
E/2001/040755	Cirrhosis	0	0	0	0	1
E/1997/009588	Cirrhosis	0	0	0	0	1
E/2004/028139	Cirrhosis	0	0	0	0	1
E/2002/000820	Liver	0	0	0	0	1
E/2003/038855	Liver	0	0	0	0	1
E/2005/010014	Liver	0	0	0	0	1
E/2004/028304	Cirrhosis	0	0	0	0	1
E/2001/031372	Liver	0	0	0	0	1
E/1996/031312	Cirrhosis	0	0	0	0	1
E/2005/015088	Cirrhosis	0	0	0	0	1
E/1999/028893-1	Cirrhosis	0	0	0	0	1
E/1999/028893-3	Cirrhosis	0	0	0	0	1
E/1999/028893-4	Cirrhosis	0	0	0	0	1
E/1999/028893-5	Cirrhosis	0	0	0	0	1
E/2001/005936	Cirrhosis	0	0	0	0	1
E/1998/009456	Cirrhosis	0	0	0	0	1
E/2007/026775	Cirrhosis	0	0	0	0	1
E/2008/029361	Cirrhosis	0	0	0	0	1
E/2010/024944	Cirrhosis	0	0	0	0	1
E/2005/034265	Cirrhosis	0	0	0	0	1
E/2005/012216	Cirrhosis	0	0	0	0	1
E/2005/037923	Liver	0	0	0	0	1
E/2005/039994	Cirrhosis	0	0	0	0	1
E/2005/025841	Cirrhosis	0	0	0	0	1
E/1997/004270	Cirrhosis	0	0	0	0	1

E/1998/002519	Cirrhosis	0	0	0	0	1
E/1998/042449	Cirrhosis	0	0	0	0	1
E/1999/002809	Cirrhosis	0	0	0	0	1
E/1998/003592	Cirrhosis	0	0	0	0	1
E/2005/024190	Cirrhosis	0	0	0	0	1
E/2005/012583	HCC	2	0	x	G2	1
E/2005/012583	Cirrhosis	0	0	0	0	1
E/2005/012583	Cirrhosis	0	0	0	0	1
E/1994/021334	Cirrhosis	0	0	0	0	1
E/1988/041821	Cirrhosis	0	0	0	0	1
E/1988/045489	Cirrhosis	0	0	0	0	1
E/1989/048366	Cirrhosis	0	0	0	0	1
E/1992/049560	Cirrhosis	0	0	0	0	1
E/1994/021334	Cirrhosis	0	0	0	0	1
E/1992/058644	Cirrhosis	0	0	0	0	1
E/1989/017053	Cirrhosis	0	0	0	0	1
E/1989/041327	Cirrhosis	0	0	0	0	1
E/1989/021987	Cirrhosis	0	0	0	0	1
E/1991/052577	Cirrhosis	0	0	0	0	1
E/1991/052469	Cirrhosis	0	0	0	0	1
E/1992/021074	Cirrhosis	0	0	0	0	1
E/1992/050504	Cirrhosis	0	0	0	0	1
E/1991/011476	Cirrhosis	0	0	0	0	1
E/1991/011475	Cirrhosis	0	0	0	0	1
E/1995/025186	Cirrhosis	0	0	0	0	1
E/1991/041134	Cirrhosis	0	0	0	0	1
K/2003/008654	HCC				G4	1
E/2010/023237	Cirrhosis	0	0 (0/00)	0	0	1
E/2009/020375	HCC	2	x	x	G1	1
E/2011/015155	Cirrhosis	0	0	0	0	1
E/2007/004509	HCC	2	x	x	G3	2
E/1996/011606	HCC	4	0	x	G2	2
E/2004/007677	HCC	2			G1	2
E/2005/009944	HCC	3	x	x	G2	2
E/2009/044075	HCC	2	x		G2	2
E/1997/014451	HCC	4	x	x	G2	2
E/2009/028425	HCC	2	1	x	G3	2
E/2007/025835	HCC	2	x	x	G3	2
E/1995/048712	HCC	2	0	x	G2	2
E/2009/002258	HCC	3	x	x	G3	2
E/2006/000447	HCC	3	0	x	G2	2
E/2010/011453	HCC	2	0		G3	2
E/2006/004144	HCC	1	x	x	G3	2

E/2009/008389	HCC	3	x	x	G2	2
E/2010/032451	HCC	2	x	x	G3	2
E/2010/037089	HCC	2a	0		G4	2
E/2011/012989	HCC	2	0	x	G2	2
E/1996/005033	HCC	2	0	x	G2	2
E/1991/052469	HCC					2
E/1994/014307	HCC					2
E/1991/052469	HCC					2
E/2003/024605	HCC	1	x	x	G3	2
E/2002/018495	HCC	1			G2	2
E/1992/015858	HCC					2
E/1992/061426	HCC					2
E/1989/021987	HCC					2
E/1991/052469	HCC					2
E/1993/020702	HCC					2
E/1991/011475	HCC					2
E/1991/011475	HCC					2
K/2003/006266	HCC					2
K/2003/008445	HCC					2
E/2004/018421	Cirrhosis	0	0	0	0	2
E/2003/005487	Cirrhosis	0	0	0	0	2
E/2003/000607	Cirrhosis	0	0	0	0	2
E/1998/003592	Liver	0	0	0	0	2
E/2005/040894	Cirrhosis	0	0	0	0	2
E/2005/037579	HCC	1	0	x	G2	3
E/1997/014451	HCC	4	x	x	G2	3

Supplementary Table 3. List of Kdm6a bound genes

gene_name	transcr_id	l2fc_on_vs_off	distance_to_tss	seqnames	start	end
Kdm6a	ENSMUST00000225336.1	-7,942166999	0	chrX	18245964	18246418
E130202H07Rik	ENSMUST00000228107.1	-5,797167797	5585	chr14	76520775	76521006
Gm34417	ENSMUST00000228293.1	-5,788317985	6493	chr14	1,02E+08	1,02E+08
Ccl8	ENSMUST00000009329.2	-5,666514102	752	chr11	82115939	82116137
Fank1	ENSMUST00000211077.1	-5,662307413	3921	chr7	1,34E+08	1,34E+08
Gm26972	ENSMUST00000237281.1	-5,612479589	0	chr18	63417065	63417275
Mir7681	ENSMUST00000184834.1	-5,592479859	3834	chr1	53845372	53845589
Cyb5r3	ENSMUST00000162178.7	-5,560254063	7	chr15	83157073	83157392
Kdm5b	ENSMUST00000133725.1	-5,529296406	11848	chr1	1,35E+08	1,35E+08
Bcl2l1	ENSMUST00000173755.1	-5,509397275	4551	chr2	1,53E+08	1,53E+08
Hipk2	ENSMUST00000114855.2	-5,459017517	27448	chr6	38845357	38845571
R3hdm1	ENSMUST00000191016.6	-5,45889499	239	chr1	1,28E+08	1,28E+08
Ech1	ENSMUST00000066264.12	-5,44127647	0	chr7	28824875	28825284
Rassf8	ENSMUST00000111704.7	-5,32031334	995	chr6	1,46E+08	1,46E+08
Tcp11l1	ENSMUST00000132600.1	-5,312544142	620	chr2	1,05E+08	1,05E+08
Gm15545	ENSMUST00000141011.1	-5,296679568	0	chr7	44986544	44986924
Gm15545	ENSMUST00000141011.1	-5,296679568	0	chr7	44986544	44986924
Ndst1	ENSMUST00000237070.1	-5,294799494	9636	chr18	60722680	60722873
Pfdn1	ENSMUST00000237499.1	-5,265760731	11114	chr18	36415321	36415608
Akap6	ENSMUST00000095737.4	-5,251163528	2886	chr12	52702271	52702695
Gm14002	ENSMUST00000123574.1	-5,231554743	4079	chr2	1,25E+08	1,25E+08
Gm14002	ENSMUST00000123574.1	-5,231554743	4079	chr2	1,25E+08	1,25E+08
Gm28694	ENSMUST00000190681.1	-5,223390795	25060	chr1	1,57E+08	1,57E+08
Lipc	ENSMUST00000215814.1	-5,206425424	11257	chr9	70834783	70835168
Gm49968	ENSMUST00000234828.1	-5,200106132	2552	chr18	11915685	11915998
Gm49968	ENSMUST00000234828.1	-5,200106132	2552	chr18	11915685	11915998
Tbc1d31	ENSMUST00000022992.12	-5,188320755	19629	chr15	57892247	57892569
Kctd9	ENSMUST00000078053.12	-5,185483385	0	chr14	67715859	67716104
Mrpl48	ENSMUST00000127253.1	-5,17375047	6785	chr7	1,01E+08	1,01E+08
Gm24407	ENSMUST00000177864.1	-5,167936122	53	chr5	1,15E+08	1,15E+08
Gm25767	ENSMUST00000158572.1	-5,163603226	7213	chr5	36678873	36679094
Gm22031	ENSMUST00000116945.1	-5,158247896	22638	chr15	3580325	3580633
Gm44507	ENSMUST00000206249.1	-5,155977038	3381	chr7	98391474	98391818
BB123696	ENSMUST00000223209.1	-5,147208315	5989	chr13	52735913	52736135
Rab27b	ENSMUST00000117692.7	-5,137674685	8626	chr18	69992284	69992679
I830134H01Rik	ENSMUST00000181592.1	-5,126132437	18468	chr19	38032992	38033305
Gm16278	ENSMUST00000148064.1	-5,095731414	841	chr17	25919932	25920497
Gm16278	ENSMUST00000148064.1	-5,095731414	841	chr17	25919932	25920497
Mroh4	ENSMUST00000176767.1	-5,085865187	6031	chr15	74622171	74622399
3300002A11Rik	ENSMUST00000046994.1	-5,077809375	5474	chr12	99351874	99352247

3300002A11Rik	ENSMUST00000046994.1	-5,077809375	5474	chr12	99351874	99352247
Scamp1	ENSMUST00000022197.14	-5,067185826	7607	chr13	94193482	94193702
Sp2	ENSMUST00000062652.6	-5,058172621	23562	chr11	96977783	96977996
Gm29243	ENSMUST00000188318.1	-5,032709454	8397	chr8	34225258	34225499
Gm50021	ENSMUST00000234097.1	-5,025667315	32938	chr17	74819878	74820059
Gm50021	ENSMUST00000234097.1	-5,025667315	32938	chr17	74819878	74820059
Gm43441	ENSMUST00000201458.1	-4,997884217	72154	chr6	36515283	36515622
Clec11a	ENSMUST00000004587.10	-4,986917958	2365	chr7	44305054	44305512
Pde8b	ENSMUST00000160957.8	-4,953116574	4039	chr13	95038483	95038982
Gm34005	ENSMUST00000215834.1	-4,945911105	3061	chr9	75960936	75961142
Gm50363	ENSMUST00000237942.1	-4,94498443	9230	chr19	16562735	16562929
Gm11623	ENSMUST00000120176.1	-4,93856907	39569	chr11	1,05E+08	1,05E+08
Gm16938	ENSMUST00000205893.1	-4,932294215	5602	chr7	98171135	98171591
Gm33111	ENSMUST00000219954.1	-4,930075773	54776	chr12	32018261	32018509
Gm15599	ENSMUST00000122283.2	-4,924475912	110607	chr17	5221375	5221612
Gm47416	ENSMUST00000217446.1	-4,883628006	6339	chr9	1,03E+08	1,03E+08
Ngf	ENSMUST00000106925.8	-4,882918247	4584	chr3	1,02E+08	1,02E+08
Sf1	ENSMUST00000113488.7	-4,880801645	0	chr19	6363473	6363830
Sf1	ENSMUST00000113488.7	-4,880801645	0	chr19	6363473	6363830
Cda	ENSMUST00000030535.3	-4,8782693	4903	chr4	1,38E+08	1,38E+08
Gmfg	ENSMUST00000108292.8	-4,874665285	814	chr7	28436418	28436632
Hecw2	ENSMUST00000152870.1	-4,870606753	357	chr1	53838386	53838659
Gm15774	ENSMUST00000141810.2	-4,86559528	280	chr7	1,21E+08	1,21E+08
Gm15774	ENSMUST00000141810.2	-4,86559528	280	chr7	1,21E+08	1,21E+08
D930028M14Rik	ENSMUST00000186424.1	-4,839290361	1165	chr7	25153720	25153903
D930028M14Rik	ENSMUST00000186424.1	-4,839290361	1165	chr7	25153720	25153903
Prkar2b	ENSMUST00000036497.15	-4,833135435	7658	chr12	31950299	31950817
Gm42892	ENSMUST00000196227.1	-4,82689232	10409	chr3	1,17E+08	1,17E+08
Gm22923	ENSMUST00000174952.1	-4,821654224	2526	chr16	75766850	75767064
Gm3436	ENSMUST00000179930.2	-4,803314237	4621	chr9	70847768	70847954
Sertad3	ENSMUST00000068641.7	-4,803035679	827	chr7	27474597	27474819
Cdkn2d	ENSMUST00000213407.1	-4,796870932	2301	chr9	21291288	21291634
Tarbp2	ENSMUST00000141266.7	-4,776811406	0	chr15	1,03E+08	1,03E+08
Arhgef28	ENSMUST00000225663.1	-4,765562005	419	chr13	97927971	97928210
Fbxw2	ENSMUST00000145660.7	-4,737598276	744	chr2	34813484	34813830
Mcc	ENSMUST00000164666.5	-4,728547104	43446	chr18	44472728	44473136
F830112A20Rik	ENSMUST00000194249.1	-4,725423956	492	chr1	36923291	36923533
F830112A20Rik	ENSMUST00000194249.1	-4,725423956	492	chr1	36923291	36923533
Sipa1l3	ENSMUST00000183081.1	-4,713286357	22803	chr7	29471545	29471749
Rapgef1	ENSMUST00000147488.1	-4,698170167	9408	chr2	29711892	29712079
Spata13	ENSMUST00000162945.1	-4,693276307	6337	chr14	60726313	60726568
Kalrn	ENSMUST00000132569.1	-4,69176191	47149	chr16	34559900	34560113
Fkbp2	ENSMUST00000237119.1	-4,683031987	1859	chr19	6980251	6980437
Akap13	ENSMUST00000207998.1	-4,681349108	663	chr7	75642368	75642655

Gm13472	ENSMUST00000120948.1	-4,666264582	53584	chr2	48441623	48442089
F930017D23Rik	ENSMUST00000217420.1	-4,665071782	11133	chr10	43604574	43605110
Gm41442	ENSMUST00000232234.1	-4,663752279	434	chr16	31235253	31235687
Gne	ENSMUST00000133709.7	-4,661988493	3006	chr4	44042750	44042948
Mir3109	ENSMUST00000175301.1	-4,660653063	173	chr9	69456554	69456770
Dnah3	ENSMUST00000208910.1	-4,649184468	0	chr7	1,2E+08	1,2E+08
Reep3	ENSMUST00000217841.1	-4,647515805	5095	chr10	67016769	67016951
Reep3	ENSMUST00000217841.1	-4,647515805	5095	chr10	67016769	67016951
Itgb1bp1	ENSMUST00000172962.1	-4,642834268	13066	chr12	21227321	21227758
Gm49735	ENSMUST00000232267.1	-4,641973331	3874	chr16	43697973	43698257
Slc16a10	ENSMUST00000213488.1	-4,636756883	1774	chr10	40073375	40073616
Snx9	ENSMUST00000231803.1	-4,628520821	4866	chr17	5846220	5846401
Synpo	ENSMUST00000137894.1	-4,625873768	299	chr18	60606184	60606435
Kdm2a	ENSMUST00000047898.13	-4,618277064	732	chr19	4315153	4315407
Tiam2	ENSMUST00000226748.1	-4,615456223	3143	chr17	3442918	3443136
Gm13274	ENSMUST00000137624.1	-4,615338776	6606	chr4	88745539	88745955
Hspa2	ENSMUST00000080449.6	-4,611274705	0	chr12	76404137	76404381
Gm47494	ENSMUST00000221204.1	-4,601612725	45938	chr13	11864188	11864411
Pxylp1	ENSMUST00000136987.1	-4,600188667	2407	chr9	96863212	96863398
Gm8210	ENSMUST00000178557.1	-4,594916405	19465	chr1	43169463	43169690
Zfp469	ENSMUST00000187142.2	-4,594916155	72147	chr8	1,22E+08	1,22E+08
Izumo4	ENSMUST00000218184.1	-4,579719465	0	chr10	80701923	80702576
Izumo4	ENSMUST00000218184.1	-4,579719465	0	chr10	80701923	80702576
AI480526	ENSMUST00000162697.1	-4,570450508	0	chr5	1,23E+08	1,23E+08
AI480526	ENSMUST00000162697.1	-4,570450508	0	chr5	1,23E+08	1,23E+08
Smad7	ENSMUST00000172718.1	-4,568740657	9988	chr18	75384904	75385167
Ap5s1	ENSMUST00000153097.2	-4,562747878	1207	chr2	1,31E+08	1,31E+08
Gm35715	ENSMUST00000197421.1	-4,550117535	14947	chr9	1,11E+08	1,11E+08
Abhd11	ENSMUST00000111216.7	-4,544578045	0	chr5	1,35E+08	1,35E+08
Abhd11	ENSMUST00000111216.7	-4,544578045	0	chr5	1,35E+08	1,35E+08
Mir1961	ENSMUST00000158643.1	-4,543966632	24594	chr5	92763636	92763856
Ankrd55	ENSMUST00000223871.1	-4,535616594	7807	chr13	1,12E+08	1,12E+08
n-R5s188	ENSMUST00000122646.1	-4,534393045	38663	chr4	82400447	82400746
Kyat1	ENSMUST00000149522.1	-4,534114492	8658	chr2	30199782	30200100
Gm49319	ENSMUST00000228533.1	-4,532815223	11414	chr14	46516428	46516761
Gm33055	ENSMUST00000234743.1	-4,52635846	5097	chr17	79738973	79739213
Baiap2	ENSMUST00000146960.1	-4,525501407	15896	chr11	1,2E+08	1,2E+08
Praf2	ENSMUST00000033489.7	-4,523035349	0	chrX	7728237	7728468
Praf2	ENSMUST00000033489.7	-4,523035349	0	chrX	7728237	7728468
Nupr1	ENSMUST00000151105.7	-4,520708413	1503	chr7	1,27E+08	1,27E+08
Gm34068	ENSMUST00000193310.1	-4,519737859	29468	chr1	1,84E+08	1,84E+08
Arhgef10l	ENSMUST00000154979.1	-4,507401734	41784	chr4	1,41E+08	1,41E+08
Ssc4d	ENSMUST00000153823.7	-4,506323251	431	chr5	1,36E+08	1,36E+08
C2cd3	ENSMUST00000051777.14	-4,502092685	0	chr7	1E+08	1E+08
C2cd3	ENSMUST00000051777.14	-4,502092685	0	chr7	1E+08	1E+08

Gm49546	ENSMUST00000229200.1	-4,498151221	1119	chr16	31291054	31291410
Srgap1	ENSMUST00000162710.1	-4,492020158	8513	chr10	1,22E+08	1,22E+08
Btd	ENSMUST00000090147.6	-4,491809643	21080	chr14	31662139	31663393
Gm4876	ENSMUST00000166070.1	-4,491466339	43	chr6	17171434	17171687
Eya4	ENSMUST00000218956.1	-4,488717158	19812	chr10	23203306	23203618
Babam2	ENSMUST00000202270.1	-4,485195036	34464	chr5	31750545	31750918
Zbtb7a	ENSMUST00000117956.1	-4,48513751	366	chr10	81137406	81137586
Thrap3	ENSMUST00000163176.1	-4,474552848	1650	chr4	1,26E+08	1,26E+08
Rasa3	ENSMUST00000132439.2	-4,471288412	46771	chr8	13644868	13645178
Ptar1	ENSMUST00000099560.4	-4,468263625	10499	chr19	23698245	23698473
Myct1	ENSMUST00000051809.9	-4,466446101	95685	chr10	5497374	5498089
Gm29099	ENSMUST00000187370.1	-4,464628062	143654	chr1	92180376	92180855
Gm27970	ENSMUST00000184854.1	-4,460759053	45003	chr19	17011376	17011777
Celf4	ENSMUST00000225927.1	-4,458161749	6575	chr18	25510797	25511063
Gm5444	ENSMUST00000065956.4	-4,454439733	0	chr13	4771429	4771873
Mir5623	ENSMUST00000176631.1	-4,45273445	27327	chr19	58022830	58023839
Gm33570	ENSMUST00000207608.1	-4,450704442	0	chr7	66580685	66580988
Ctsb	ENSMUST00000225540.1	-4,449531339	3133	chr14	63132205	63132384
Gm11453	ENSMUST00000117318.1	-4,435593097	8347	chr2	1,63E+08	1,63E+08
Itsn1	ENSMUST00000114001.7	-4,434706148	17	chr16	91728766	91729263
Itsn1	ENSMUST00000114001.7	-4,434706148	17	chr16	91728766	91729263
Mir763	ENSMUST00000103249.1	-4,426116245	24798	chr10	1,2E+08	1,2E+08
Gm37024	ENSMUST00000192224.1	-4,419879962	6238	chr3	30482004	30482187
Ccnk	ENSMUST00000221167.1	-4,41507477	0	chr12	1,08E+08	1,08E+08
Gm12304	ENSMUST00000120312.1	-4,404423291	3131	chr11	68092204	68092448
Sgce	ENSMUST00000204897.1	-4,400624768	3275	chr6	4740419	4740652
Fbxo17	ENSMUST00000167118.1	-4,394854209	4321	chr7	28727870	28728178
Parm1	ENSMUST00000040576.9	-4,394565384	15360	chr5	91532977	91533300
Rfx1	ENSMUST00000211046.1	-4,393546555	83	chr8	84066484	84066750
Slc44a1	ENSMUST00000107647.7	-4,393532337	5658	chr4	53446344	53446685
Dazap1	ENSMUST00000156935.7	-4,393225232	0	chr10	80261131	80261679
Hivep3	ENSMUST00000227491.1	-4,387266163	99585	chr4	1,2E+08	1,2E+08
Mknk2	ENSMUST00000199949.1	-4,385632136	4425	chr10	80676373	80676747
Gm22685	ENSMUST00000082848.1	-4,382144817	91751	chr4	49197346	49197546
Gm5802	ENSMUST00000224324.1	-4,377366767	9043	chr14	62530819	62531449
Aig1	ENSMUST00000162869.1	-4,368990723	3384	chr10	13832681	13833287
Ifi203	ENSMUST00000156895.7	-4,35855929	6848	chr1	1,74E+08	1,74E+08
Ece1	ENSMUST00000129607.1	-4,357732622	17223	chr4	1,38E+08	1,38E+08
Gm10280	ENSMUST00000212238.1	-4,355508384	91505	chr8	1,13E+08	1,13E+08
Pde3b	ENSMUST00000140007.1	-4,354487573	25669	chr7	1,14E+08	1,14E+08
Gtf2h2	ENSMUST00000232447.1	-4,349358617	1122	chr13	1E+08	1E+08
Sp1	ENSMUST00000170884.7	-4,344748567	0	chr15	1,02E+08	1,02E+08
Cntrob	ENSMUST00000148490.1	-4,343585387	1277	chr11	69311322	69311880
Lgals3	ENSMUST00000142734.7	-4,341398634	1564	chr14	47371348	47372248
4933413C19Rik	ENSMUST00000162380.1	-4,335164201	43978	chr19	28624513	28624826

Gm46587	ENSMUST00000234992.1	-4,328213716	4791	chr17	87563970	87564611
Nfatc2	ENSMUST00000171689.7	-4,32590896	107	chr2	1,69E+08	1,69E+08
Jag1	ENSMUST00000028735.7	-4,322988167	13407	chr2	1,37E+08	1,37E+08
Gm7041	ENSMUST00000201227.1	-4,317315184	34789	chr5	1,51E+08	1,51E+08
Ugcg	ENSMUST00000133996.1	-4,311462863	7120	chr4	59196680	59196900
Gm44130	ENSMUST00000205157.1	-4,306486537	9447	chr6	71422476	71422656
Gm23517	ENSMUST00000082830.1	-4,30019862	3495	chr15	85347187	85347567
Cdca3	ENSMUST00000150120.7	-4,298828297	0	chr6	1,25E+08	1,25E+08
Cdca3	ENSMUST00000150120.7	-4,298828297	0	chr6	1,25E+08	1,25E+08
Me3	ENSMUST00000159491.7	-4,29698641	20249	chr7	89716237	89716481
Crlf2	ENSMUST00000198960.1	-4,287709619	2430	chr5	1,1E+08	1,1E+08
Syne2	ENSMUST00000157033.1	-4,28404725	1815	chr12	75820167	75820370
Ubp1	ENSMUST00000009885.13	-4,276802157	0	chr9	1,14E+08	1,14E+08
Itga2b	ENSMUST00000145925.1	-4,275353055	0	chr11	1,02E+08	1,02E+08
Itgb6	ENSMUST00000133887.7	-4,268527231	5286	chr2	60639719	60640117
Dtnbp1	ENSMUST00000223265.1	-4,260173906	6982	chr13	44939409	44939763
Brpf1	ENSMUST00000113122.7	-4,251794382	295	chr6	1,13E+08	1,13E+08
Baiap2l1	ENSMUST00000129287.1	-4,251524116	35508	chr5	1,44E+08	1,44E+08
Baiap2l1	ENSMUST00000129287.1	-4,251524116	35508	chr5	1,44E+08	1,44E+08
Hebp1	ENSMUST00000045855.8	-4,248323773	3980	chr6	1,35E+08	1,35E+08
Runx2	ENSMUST00000238400.1	-4,245490325	3906	chr17	44500394	44500582
Smim14	ENSMUST00000200111.2	-4,235348275	1549	chr5	65454571	65455478
Smim14	ENSMUST00000200111.2	-4,235348275	1549	chr5	65454571	65455478
Mrps35	ENSMUST00000123139.1	-4,23258102	3304	chr6	1,47E+08	1,47E+08
Gm49011	ENSMUST00000227294.1	-4,225087881	39888	chr14	79065396	79065820
Rnu3b1	ENSMUST00000183812.1	-4,223790711	149	chr11	87443388	87443756
Rnu3b1	ENSMUST00000183812.1	-4,223790711	149	chr11	87443388	87443756
Rnu3b1	ENSMUST00000183812.1	-4,223790711	149	chr11	87443388	87443756
Gtpbp1	ENSMUST00000046463.9	-4,22327492	170	chr15	79690466	79690674
Gm16223	ENSMUST00000087332.4	-4,215440256	87841	chr5	42155803	42156149
Gm38505	ENSMUST00000174005.2	-4,214766242	33625	chr3	34331799	34332087
Gm38505	ENSMUST00000174005.2	-4,214766242	33625	chr3	34331799	34332087
Gm15576	ENSMUST00000117806.2	-4,206681847	5768	chr6	1,01E+08	1,01E+08
1700055D18Rik	ENSMUST00000095105.1	-4,20559019	158	chr4	45012469	45012671
Gse1	ENSMUST00000211997.1	-4,195820146	1008	chr8	1,21E+08	1,21E+08
Gse1	ENSMUST00000211997.1	-4,195820146	1008	chr8	1,21E+08	1,21E+08
Lima1	ENSMUST00000171450.1	-4,195444095	11450	chr15	99852680	99853232
Ltbp1	ENSMUST00000234327.1	-4,193203893	4656	chr17	75305318	75305499
Col4a1	ENSMUST00000209000.1	-4,185449253	14726	chr8	11259730	11260413
Gm43676	ENSMUST00000197532.1	-4,182840572	7955	chr5	1,11E+08	1,11E+08
Zcchc9-ps	ENSMUST00000117884.1	-4,182390317	42217	chr2	1,41E+08	1,41E+08
Gsk3b	ENSMUST00000023507.12	-4,179269779	0	chr16	38088556	38089303
Ppfibp1	ENSMUST00000111623.8	-4,17782447	21434	chr6	1,47E+08	1,47E+08
Gm1604a	ENSMUST00000231840.1	-4,176046972	3072	chr17	7985596	7985946
Gm12354	ENSMUST00000124319.7	-4,173001343	376	chr4	19717110	19717635

Gstcd	ENSMUST00000080583.5	-4,170571036	1303	chr3	1,33E+08	1,33E+08
Snrpd2	ENSMUST00000049294.3	-4,169772236	0	chr7	19149216	19149738
Snrpd2	ENSMUST00000049294.3	-4,169772236	0	chr7	19149216	19149738
Susd1	ENSMUST00000107544.1	-4,16936062	412	chr4	59315168	59315370
8430426J06 Rik	ENSMUST00000230042.1	-4,16849697	1930	chr15	81247738	81248097
Gm45353	ENSMUST00000210581.1	-4,164923073	6474	chr8	1,22E+08	1,22E+08
Shoc2	ENSMUST00000169861.8	-4,163384859	0	chr19	53944032	53944749
Shoc2	ENSMUST00000169861.8	-4,163384859	0	chr19	53944032	53944749
Dsc2	ENSMUST00000128464.1	-4,159093918	2674	chr18	20034155	20034705
Gm9913	ENSMUST00000066157.1	-4,157971769	12604	chr2	1,25E+08	1,25E+08
A630001O12Rik	ENSMUST00000212914.1	-4,154300689	10429	chr8	1,27E+08	1,27E+08
A630001O12Rik	ENSMUST00000212914.1	-4,154300689	10429	chr8	1,27E+08	1,27E+08
Bcar3	ENSMUST00000198659.1	-4,154032929	765	chr3	1,23E+08	1,23E+08
Nceh1	ENSMUST00000140872.1	-4,149277719	25732	chr3	27214175	27214707
Oxr1	ENSMUST00000229769.1	-4,149067978	16688	chr15	41727294	41727514
Gm45030	ENSMUST00000207575.2	-4,137171543	44534	chr16	76472279	76472627
Gm23193	ENSMUST00000102427.1	-4,135796029	9177	chr8	1,11E+08	1,11E+08
Gm29591	ENSMUST00000188215.1	-4,129081001	6296	chr6	17354031	17354361
Gm6978	ENSMUST00000238142.1	-4,128896848	22148	chr18	64634140	64634362
Plekhm1	ENSMUST00000184350.1	-4,125229785	15545	chr11	1,03E+08	1,03E+08
Mgst3	ENSMUST00000028005.2	-4,123486824	15557	chr1	1,67E+08	1,67E+08
4933426B08Rik	ENSMUST00000231816.1	-4,120381657	0	chr17	6808055	6808886
4933426B08Rik	ENSMUST00000231816.1	-4,120381657	0	chr17	6808055	6808886
Nt5dc3	ENSMUST00000099396.2	-4,119559987	99	chr10	86778676	86778905
Gm7730	ENSMUST00000212486.1	-4,117522369	13860	chr8	69996337	69996688
Gm5427	ENSMUST00000217680.1	-4,114729064	30456	chr10	98684257	98684657
Vmn1r43	ENSMUST00000089418.4	-4,114596659	1307	chr6	89867819	89868153
Vmn1r43	ENSMUST00000089418.4	-4,114596659	1307	chr6	89867819	89868153
Bcar1	ENSMUST00000212147.1	-4,112449693	7024	chr8	1,12E+08	1,12E+08
Vps13d	ENSMUST00000141208.7	-4,112368598	17836	chr4	1,45E+08	1,45E+08
Tmigd1	ENSMUST00000136899.1	-4,111255805	4237	chr11	76908801	76909066
Nmu	ENSMUST00000031146.2	-4,107193285	12747	chr5	76346244	76346610
Polr2k-ps	ENSMUST00000120170.1	-4,104246076	16244	chr11	95777737	95778053
Hnrnpl	ENSMUST00000174755.7	-4,103605055	0	chr7	28810363	28811240
Hnrnpl	ENSMUST00000174755.7	-4,103605055	0	chr7	28810363	28811240
Lmo7	ENSMUST00000159806.1	-4,097051896	1635	chr14	1,02E+08	1,02E+08
4930417H01Rik	ENSMUST00000123403.1	-4,095876546	11222	chr2	1,23E+08	1,23E+08
Gm13480	ENSMUST00000125095.1	-4,095661246	3763	chr2	49809741	49810139
Gm13480	ENSMUST00000125095.1	-4,095661246	3763	chr2	49809741	49810139
Cdc37	ENSMUST00000019615.10	-4,091584735	1831	chr9	21135055	21135535
Gsap	ENSMUST00000196035.1	-4,088799859	4103	chr5	21246711	21247055
Gm31025	ENSMUST00000222553.1	-4,087353886	7598	chr12	28002608	28002803
Zdhhc18	ENSMUST00000138639.1	-4,083213624	816	chr4	1,34E+08	1,34E+08

Naif1	ENSMUST00000048431.2	-4,082975892	1155	chr2	32451614	32451926
Fez2	ENSMUST00000234900.1	-4,082773688	1376	chr17	78401697	78402292
Emp1	ENSMUST00000032330.15	-4,080071308	0	chr6	1,35E+08	1,35E+08
Mir1957a	ENSMUST00000157927.1	-4,076688766	3651	chr4	1,19E+08	1,19E+08
Calr-ps	ENSMUST00000121776.1	-4,069027788	107515	chr2	1,41E+08	1,41E+08
Sptbn1	ENSMUST00000149117.1	-4,064928261	6068	chr11	30210382	30210590
Ccl26	ENSMUST00000094226.1	-4,063661025	37722	chr5	1,36E+08	1,36E+08
Gm42838	ENSMUST00000196503.1	-4,061344577	13986	chr5	1,25E+08	1,25E+08
Fam76b	ENSMUST00000059579.11	-4,06131356	102	chr9	13827107	13827613
Ifrd2	ENSMUST00000010192.10	-4,056691666	0	chr9	1,08E+08	1,08E+08
Mir100hg	ENSMUST00000233563.1	-4,054043753	5172	chr9	41480234	41480455
Mir100hg	ENSMUST00000233563.1	-4,054043753	5172	chr9	41480234	41480455
Mir100hg	ENSMUST00000233563.1	-4,054043753	5172	chr9	41480234	41480455
Tmem230	ENSMUST00000028816.8	-4,053997414	27026	chr2	1,32E+08	1,32E+08
Ddr2	ENSMUST00000192312.5	-4,053681549	3639	chr1	1,7E+08	1,7E+08
Gm42819	ENSMUST00000199630.1	-4,051872737	2948	chr3	98027486	98027738
Smim6	ENSMUST00000132961.1	-4,050957033	0	chr11	1,16E+08	1,16E+08
Smim6	ENSMUST00000132961.1	-4,050957033	0	chr11	1,16E+08	1,16E+08
Stk24	ENSMUST00000226158.1	-4,048248725	3903	chr14	1,21E+08	1,21E+08
Gm49471	ENSMUST00000229249.1	-4,045026272	1466	chr15	79062868	79063194
Gm14321	ENSMUST00000127441.1	-4,044309509	0	chr2	1,68E+08	1,68E+08
Gm4861	ENSMUST00000198629.4	-4,042061763	2501	chr3	1,38E+08	1,38E+08
Pdlim4	ENSMUST00000151948.1	-4,038301221	925	chr11	54062231	54062797
Cic	ENSMUST00000169266.7	-4,037796656	0	chr7	25267622	25268337
Gm12785	ENSMUST00000122052.1	-4,037093056	24276	chr4	1,01E+08	1,01E+08
4930583P06Rik	ENSMUST00000136658.1	-4,036433888	91820	chr2	1,24E+08	1,24E+08
Heg1	ENSMUST00000132797.1	-4,034266131	7476	chr16	33739079	33739464
Gm10603	ENSMUST00000184875.1	-4,029649215	1487	chr7	1E+08	1E+08
Gm10603	ENSMUST00000184875.1	-4,029649215	1487	chr7	1E+08	1E+08
Rnu5g	ENSMUST00000093721.1	-4,028195915	547	chr9	65200968	65201178
Lipa	ENSMUST00000237617.1	-4,027644914	3967	chr19	34518097	34518366
Tanc1	ENSMUST00000128030.1	-4,023647987	7314	chr2	59637379	59637786
Drc3	ENSMUST00000108723.8	-4,02009847	0	chr11	60352770	60353355
Drc3	ENSMUST00000108723.8	-4,02009847	0	chr11	60352770	60353355
Gm45345	ENSMUST00000211427.1	-4,018054343	5768	chr8	64844004	64844303
4930589P08Rik	ENSMUST00000129097.1	-4,015569164	39408	chr4	1,51E+08	1,51E+08
Sin3a	ENSMUST00000168678.7	-4,011029894	4	chr9	57075774	57076371
Sin3a	ENSMUST00000168678.7	-4,011029894	4	chr9	57075774	57076371
Gm37228	ENSMUST00000193574.1	-4,006658292	3146	chr3	1,34E+08	1,34E+08
Aspscr1	ENSMUST00000168947.1	-4,002082385	1401	chr11	1,21E+08	1,21E+08
Aspscr1	ENSMUST00000168947.1	-4,002082385	1401	chr11	1,21E+08	1,21E+08
2610035D17Rik	ENSMUST00000150712.1	-4,001308672	18450	chr11	1,13E+08	1,13E+08
Gm25558	ENSMUST00000158083.1	-3,993241734	29	chr9	50887204	50887920
Gm19514	ENSMUST00000206856.1	-3,989842304	18295	chr7	1,34E+08	1,34E+08

Mau2	ENSMUST00000212596.1	-3,987304369	1604	chr8	70030154	70030459
Ifngr2	ENSMUST00000130404.1	-3,985157768	3509	chr16	91564834	91565054
Gm37767	ENSMUST00000193239.1	-3,98489511	46495	chr1	1,62E+08	1,62E+08
Rpl36-ps2	ENSMUST00000136893.2	-3,979670623	11969	chr11	76589132	76589349
Ank2	ENSMUST00000182452.7	-3,978835996	13317	chr3	1,27E+08	1,27E+08
Fam129a	ENSMUST00000086267.5	-3,973751016	526	chr1	1,52E+08	1,52E+08
B930036N10Rik	ENSMUST00000165827.1	-3,973116524	0	chr1	1,72E+08	1,72E+08
Snx29	ENSMUST00000134941.1	-3,970352324	8500	chr16	11455810	11456310
Prr14	ENSMUST00000206118.1	-3,969497276	0	chr7	1,27E+08	1,27E+08
Mdc1	ENSMUST00000174124.1	-3,966604172	0	chr17	35841305	35841714
St13	ENSMUST00000172107.7	-3,966572754	6680	chr15	81356257	81356988
Rabggta	ENSMUST00000227061.1	-3,965623772	0	chr14	55715177	55715573
Gm14372	ENSMUST00000125355.1	-3,964368459	27243	chr7	1,44E+08	1,44E+08
Ppp2r3d	ENSMUST00000188509.1	-3,96191194	1262	chr9	1,24E+08	1,24E+08
Mbp	ENSMUST00000143506.7	-3,955972876	17234	chr18	82492606	82492818
5930403N24Rik	ENSMUST00000217338.1	-3,952237542	184	chr10	37141275	37141668
Wdfy2	ENSMUST00000014691.9	-3,952152723	50	chr14	62837272	62837627
Gm23647	ENSMUST00000175042.1	-3,95156624	4145	chr8	1,24E+08	1,24E+08
Dop1b	ENSMUST00000227156.1	-3,949704489	2964	chr16	93733065	93733431
Map3k5	ENSMUST00000138994.1	-3,949592508	5029	chr10	20104556	20104958
Tgm2	ENSMUST00000140923.7	-3,942280368	142	chr2	1,58E+08	1,58E+08
Tgm2	ENSMUST00000140923.7	-3,942280368	142	chr2	1,58E+08	1,58E+08
Gm41271	ENSMUST00000228108.1	-3,940057516	2960	chr15	11699772	11700138
Dnpep	ENSMUST00000189282.2	-3,939999517	964	chr1	75317520	75318011
Arhgap26	ENSMUST00000123820.1	-3,932516995	47588	chr18	39051258	39051722
Arhgap26	ENSMUST00000123820.1	-3,932516995	47588	chr18	39051258	39051722
Tmprss6	ENSMUST00000229124.1	-3,929759455	5085	chr15	78450384	78450565
Wee1	ENSMUST00000033326.9	-3,92666928	211	chr7	1,1E+08	1,1E+08
Wee1	ENSMUST00000033326.9	-3,92666928	211	chr7	1,1E+08	1,1E+08
Enpp3	ENSMUST00000220209.1	-3,923540069	2768	chr10	24823353	24823702
Rdm1	ENSMUST00000133727.7	-3,922729636	0	chr11	1,02E+08	1,02E+08
Rdm1	ENSMUST00000133727.7	-3,922729636	0	chr11	1,02E+08	1,02E+08
Gm26086	ENSMUST00000157902.1	-3,919962146	16706	chr13	43068856	43069132
Arntl	ENSMUST00000209495.1	-3,919623905	2311	chr7	1,13E+08	1,13E+08
Sgk2	ENSMUST00000018012.13	-3,918814386	0	chr2	1,63E+08	1,63E+08
B930025P03Rik	ENSMUST00000207718.1	-3,914225501	4295	chr8	10865690	10866126
Gm22847	ENSMUST00000157707.1	-3,913353489	4497	chr5	34535148	34535591
Gm36954	ENSMUST00000195362.1	-3,912135124	37995	chr11	9666952	9667265
Trpm4	ENSMUST00000209239.1	-3,906967733	6480	chr7	45313105	45313950
Fads6	ENSMUST00000056153.7	-3,906387757	2374	chr11	1,15E+08	1,15E+08
Tmem140	ENSMUST00000201749.1	-3,90438244	998	chr6	34865780	34866021
Coa3	ENSMUST00000017332.3	-3,903084241	0	chr11	1,01E+08	1,01E+08
Gm15843	ENSMUST00000146593.1	-3,899208505	1989	chr1	72457309	72457509
Chd3	ENSMUST00000144701.1	-3,898321514	5920	chr11	69366646	69366838

Gm37168	ENSMUST00000192965.1	-3,895241323	10472	chr1	1,91E+08	1,91E+08
Usp6nl	ENSMUST00000042503.8	-3,895028184	27120	chr2	6379855	6380144
Mir6995	ENSMUST00000183658.1	-3,89165492	41724	chr19	47315245	47315697
Twf1	ENSMUST00000023087.12	-3,889755825	2896	chr15	94574812	94575054
Mss51	ENSMUST00000022353.4	-3,889199484	3950	chr14	20478687	20478916
Gm6145	ENSMUST00000211251.1	-3,886303271	5063	chr14	1,06E+08	1,06E+08
Onecut2	ENSMUST00000175965.9	-3,884595604	37854	chr18	64377876	64378191
Gm43000	ENSMUST00000198482.1	-3,882884774	6896	chr5	16000855	16001225
Ppp1r12a	ENSMUST00000070663.5	-3,881981012	10668	chr10	1,08E+08	1,08E+08
Fars2	ENSMUST00000225048.1	-3,881780018	25473	chr13	36696097	36696452
Gm25363	ENSMUST00000158850.1	-3,878369379	5067	chr5	24825662	24825964
Nphp1	ENSMUST00000028857.13	-3,87771488	17939	chr2	1,28E+08	1,28E+08
Gpat3	ENSMUST00000145612.1	-3,87678715	14334	chr5	1,01E+08	1,01E+08
4732465J04Rik	ENSMUST00000185499.1	-3,875449737	14604	chr10	95763880	95764312
Gm12498	ENSMUST00000138374.1	-3,873494792	424	chr3	1,08E+08	1,08E+08
Gm21971	ENSMUST00000180187.1	-3,873163467	5038	chr12	1,03E+08	1,03E+08
Gm21971	ENSMUST00000180187.1	-3,873163467	5038	chr12	1,03E+08	1,03E+08
Gm21971	ENSMUST00000180187.1	-3,873163467	5038	chr12	1,03E+08	1,03E+08
Arhgap18	ENSMUST00000142284.1	-3,872704628	8448	chr10	26854625	26854893
Gm11373	ENSMUST00000129791.2	-3,867481004	1678	chr13	31327614	31327824
Tead1	ENSMUST00000171373.1	-3,866793052	5548	chr7	1,13E+08	1,13E+08
Gm36635	ENSMUST00000221152.1	-3,865172624	1748	chr12	1,12E+08	1,12E+08
Gstt2	ENSMUST00000218745.1	-3,864471439	148	chr10	75836126	75836435
Plekha5	ENSMUST00000205026.2	-3,862971396	4046	chr6	1,41E+08	1,41E+08
4632404M16Rik	ENSMUST00000197326.1	-3,856942503	1323	chr3	1,02E+08	1,02E+08
4632404M16Rik	ENSMUST00000197326.1	-3,856942503	1323	chr3	1,02E+08	1,02E+08
Mcts2	ENSMUST00000062148.8	-3,85685287	3371	chr2	1,53E+08	1,53E+08
Abhd2	ENSMUST00000037315.12	-3,856313575	39	chr7	79272682	79273159
Pxt1	ENSMUST00000051526.5	-3,855482249	2071	chr17	28931683	28931912
Il1r1	ENSMUST00000195402.1	-3,849537524	8918	chr1	40275577	40275781
Gm14137	ENSMUST00000239130.1	-3,84903841	94	chr2	1,19E+08	1,19E+08
Iqck	ENSMUST00000132148.2	-3,847968649	1397	chr7	1,19E+08	1,19E+08
Rhobtb1	ENSMUST00000164034.7	-3,847468574	22587	chr10	69185672	69185964
Gm23546	ENSMUST00000158211.1	-3,846832208	5390	chr2	33205913	33206414
Ppp2r2b	ENSMUST00000155262.1	-3,845417961	8503	chr18	42766973	42767857
Gm22697	ENSMUST00000175194.1	-3,844667935	6145	chr18	42308037	42308323
Gm47297	ENSMUST00000197891.1	-3,843090614	17341	chr11	1,2E+08	1,2E+08
Lpin2	ENSMUST00000129635.7	-3,840161608	7936	chr17	71191916	71192135
Lpin2	ENSMUST00000129635.7	-3,840161608	7936	chr17	71191916	71192135
Nav2	ENSMUST00000207743.1	-3,838505921	10729	chr7	49332365	49332744
Gm24224	ENSMUST00000158828.1	-3,829527539	15604	chr7	79336625	79336883
Gm34256	ENSMUST00000232216.1	-3,828452018	1907	chr16	31592105	31592379
Gm35333	ENSMUST00000225281.1	-3,827052366	5060	chr13	60427847	60428044
Cfap69	ENSMUST00000148193.1	-3,82035838	529	chr5	5649089	5649278

Gm13412	ENSMUST00000150621.1	-3,819941169	2530	chr2	32527563	32527750
Gm10614	ENSMUST00000181414.1	-3,819652232	173	chr8	1,21E+08	1,21E+08
Gm10614	ENSMUST00000181414.1	-3,819652232	173	chr8	1,21E+08	1,21E+08
Gm20604	ENSMUST00000174651.1	-3,81942873	10204	chr12	1,03E+08	1,03E+08
Bahcc1	ENSMUST00000118987.1	-3,818587993	18728	chr11	1,2E+08	1,2E+08
Pip5k1a	ENSMUST00000107231.1	-3,816955557	4117	chr3	95066348	95066708
4932411K12Rik	ENSMUST00000190493.1	-3,816218	10065	chr13	1,01E+08	1,01E+08
Itpr2	ENSMUST00000111673.1	-3,815009005	11000	chr6	1,46E+08	1,46E+08
Stk10	ENSMUST00000143397.1	-3,808731964	24619	chr11	32563773	32563975
Gm30192	ENSMUST00000236674.1	-3,808667077	54673	chr18	81319383	81319704
Dnal4	ENSMUST00000161646.1	-3,808615817	87	chr15	79774262	79774484
Bsdc1	ENSMUST00000048162.9	-3,804474364	0	chr4	1,29E+08	1,29E+08
Megf11	ENSMUST00000147388.1	-3,797304404	17474	chr9	64564375	64564685
Nectin2	ENSMUST00000075447.13	-3,796572186	548	chr7	19715585	19716095
Gm41386	ENSMUST00000229650.1	-3,792274585	5610	chr15	93710925	93711416
Abl2	ENSMUST00000190749.1	-3,791300013	110	chr1	1,57E+08	1,57E+08
Mylpf	ENSMUST00000206772.1	-3,789551293	0	chr7	1,27E+08	1,27E+08
Gm49410	ENSMUST00000230156.1	-3,786808401	877	chr15	77741640	77742019
Gm39321	ENSMUST00000217076.1	-3,785721901	31806	chr9	42109988	42110186
Gm29675	ENSMUST00000222202.1	-3,783702081	3342	chr13	30787777	30788235
Gm42435	ENSMUST00000196630.1	-3,783699049	2830	chr5	3501457	3501872
Gm6276	ENSMUST00000120159.1	-3,780252339	53934	chr17	75110497	75111272
Blmh	ENSMUST00000145732.2	-3,778745467	7293	chr11	76953797	76954002
2210411M09Rik	ENSMUST00000128545.2	-3,777807894	208	chr1	1,81E+08	1,81E+08
2210411M09Rik	ENSMUST00000128545.2	-3,777807894	208	chr1	1,81E+08	1,81E+08
Shc1	ENSMUST00000107417.8	-3,777112728	0	chr3	89418401	89418746
Ttll6	ENSMUST00000107680.1	-3,776902293	6613	chr11	96140834	96141365
Gm15657	ENSMUST00000148293.1	-3,772243375	6874	chr16	33777306	33777794
Pkm	ENSMUST00000213930.1	-3,770465979	1435	chr9	59659680	59659878
Mfsd13a	ENSMUST00000086969.12	-3,768718552	4130	chr19	46352347	46352749
Dixdc1	ENSMUST00000118707.1	-3,766720001	13273	chr9	50723412	50723890
Myo1e	ENSMUST00000034745.8	-3,765988714	83219	chr9	70290589	70290952
Mir22hg	ENSMUST00000149940.1	-3,760873606	0	chr11	75461170	75461586
Fstl3	ENSMUST00000168798.1	-3,760134072	0	chr10	79780339	79780730
Gm16198	ENSMUST00000118766.1	-3,760036281	9658	chr3	1,52E+08	1,52E+08
Mmp28	ENSMUST00000138780.1	-3,754115736	6708	chr11	83458096	83458361
Cdk19os	ENSMUST00000044774.8	-3,751314302	0	chr10	40348888	40349295
Cdk19os	ENSMUST00000044774.8	-3,751314302	0	chr10	40348888	40349295
Gm44699	ENSMUST00000208229.1	-3,747429358	9971	chr7	28930924	28931122
Gm44699	ENSMUST00000208229.1	-3,747429358	9971	chr7	28930924	28931122
Map3k20	ENSMUST00000135204.1	-3,744460775	3348	chr2	72289144	72289533
Gm14052	ENSMUST00000121434.1	-3,74090176	381	chr2	1,32E+08	1,32E+08
Gm48570	ENSMUST00000224720.1	-3,739620513	2574	chr13	41404867	41405258
Ms4a15	ENSMUST00000144485.1	-3,738320154	2387	chr19	10985648	10986000

Cactin	ENSMUST00000050867.7	-3,734946951	0	chr10	81320501	81321138
Gm36264	ENSMUST00000221989.1	-3,734139935	41410	chr13	9043069	9043378
Vezt	ENSMUST00000148450.7	-3,729583225	4979	chr10	93944146	93944538
Poln	ENSMUST00000202027.1	-3,728109476	30	chr5	34032346	34032715
Gm13175	ENSMUST00000139479.1	-3,727307415	12271	chr2	4445893	4446162
Rarg	ENSMUST00000043172.14	-3,726165683	3882	chr15	1,02E+08	1,02E+08
Etnk1	ENSMUST00000204947.2	-3,720903924	14315	chr6	1,43E+08	1,43E+08
Gm29642	ENSMUST00000186170.2	-3,719972755	0	chr9	25151788	25152149
Gm29642	ENSMUST00000186170.2	-3,719972755	0	chr9	25151788	25152149
B930018H19Rik	ENSMUST00000211381.1	-3,71479097	1833	chr8	34591255	34591706
Gm20428	ENSMUST00000174365.1	-3,713703393	19040	chr1	39256784	39257242
Gm17705	ENSMUST00000172285.2	-3,711557994	11	chr17	35165132	35165355
Cpne5	ENSMUST00000126993.7	-3,708702398	1019	chr17	29156632	29156896
Sult2b1	ENSMUST00000210021.1	-3,707723995	11389	chr7	45770721	45771218
Slc4a4	ENSMUST00000134303.1	-3,706683921	119045	chr5	89147139	89147763
Klf10	ENSMUST00000228416.1	-3,70574093	423	chr15	38298949	38299450
Gm24150	ENSMUST00000082759.1	-3,705040294	1954	chr3	79194121	79194419
Gm45261	ENSMUST00000211023.1	-3,703730022	1512	chr1	80447841	80448084
Glis1	ENSMUST00000125573.1	-3,703222111	11993	chr4	1,08E+08	1,08E+08
Mtmr4	ENSMUST00000146871.7	-3,701194493	0	chr11	87591837	87592248
Ext2	ENSMUST00000152344.1	-3,699682783	13944	chr2	93776507	93777172
Sec1	ENSMUST00000040636.8	-3,697562093	2484	chr7	45680172	45680389
Gm14138	ENSMUST00000122061.1	-3,696790178	9059	chr2	1,19E+08	1,19E+08
Osbpl3	ENSMUST00000136926.2	-3,693991567	20019	chr6	50372841	50373502
B430305J03Rik	ENSMUST00000066298.2	-3,69309336	1578	chr3	61363830	61364207
B430305J03Rik	ENSMUST00000066298.2	-3,69309336	1578	chr3	61363830	61364207
Gm22265	ENSMUST00000082947.1	-3,692865566	288	chr1	1,93E+08	1,93E+08
Slc25a24	ENSMUST00000140786.2	-3,692273196	32851	chr3	1,09E+08	1,09E+08
Gm23444	ENSMUST00000082922.1	-3,691494911	120	chr11	83271620	83273099
Ehf	ENSMUST00000128546.1	-3,689684238	6234	chr2	1,03E+08	1,03E+08
Zbtb20	ENSMUST00000126354.7	-3,684397641	114	chr16	42875332	42875766
Coro2a	ENSMUST00000139179.1	-3,683535413	10937	chr4	46553940	46554147
Ttc41	ENSMUST00000061458.8	-3,682460634	0	chr10	86705668	86706008
Gm21969	ENSMUST00000178644.1	-3,68163009	3810	chr4	1,4E+08	1,4E+08
Gm37769	ENSMUST00000193753.1	-3,681353099	9854	chr1	68720893	68721101
2010204K13Rik	ENSMUST00000150671.1	-3,681259083	16971	chrX	7429079	7429508
Gm12146	ENSMUST00000127604.1	-3,679501109	13365	chr11	43183836	43184058
Zyx	ENSMUST00000070635.12	-3,676824816	0	chr6	42349771	42349958
Zyx	ENSMUST00000070635.12	-3,676824816	0	chr6	42349771	42349958
Gm29358	ENSMUST00000187941.1	-3,676770849	0	chr1	74295475	74295938
Gm29358	ENSMUST00000187941.1	-3,676770849	0	chr1	74295475	74295938
Gm29358	ENSMUST00000187941.1	-3,676770849	0	chr1	74295475	74295938
Dnaaf5	ENSMUST00000026975.10	-3,674475301	89	chr5	1,39E+08	1,39E+08
Dnaaf5	ENSMUST00000026975.10	-3,674475301	89	chr5	1,39E+08	1,39E+08

Mir31	ENSMUST00000083474.1	-3,674408776	17778	chr4	88928337	88928751
Rsbn1	ENSMUST00000151927.3	-3,672278865	104	chr3	1,04E+08	1,04E+08
Gm18001	ENSMUST00000212023.1	-3,671643771	2619	chr8	83602634	83602844
Gm12804	ENSMUST00000121030.1	-3,669793814	74677	chr4	1,11E+08	1,11E+08
Gm47156	ENSMUST00000213334.1	-3,669450385	1571	chr10	93620435	93620960
Gm49427	ENSMUST00000215481.1	-3,666599435	1516	chr9	1,2E+08	1,2E+08
Gm49427	ENSMUST00000215481.1	-3,666599435	1516	chr9	1,2E+08	1,2E+08
Smyd3	ENSMUST00000194237.1	-3,659530883	30517	chr1	1,79E+08	1,79E+08
Cacna2d1	ENSMUST00000200270.1	-3,654599902	37128	chr5	16062844	16063111
2810013P06 Rik	ENSMUST00000186531.1	-3,653400711	20708	chr8	1,23E+08	1,23E+08
2810013P06 Rik	ENSMUST00000186531.1	-3,653400711	20708	chr8	1,23E+08	1,23E+08
2900057B2 0Rik	ENSMUST00000236092.1	-3,653070734	19064	chr18	76110623	76110856
Ppm1b	ENSMUST00000112305.9	-3,652592571	413	chr17	84957156	84957536
Ppm1b	ENSMUST00000112305.9	-3,652592571	413	chr17	84957156	84957536
Galnt2	ENSMUST00000142547.1	-3,651421725	16117	chr8	1,24E+08	1,24E+08
Galnt2	ENSMUST00000142547.1	-3,651421725	16117	chr8	1,24E+08	1,24E+08
2610027F03 Rik	ENSMUST00000185443.1	-3,650645524	33278	chr1	1,21E+08	1,21E+08
5830428M2 4Rik	ENSMUST00000222980.1	-3,649046495	640	chr12	69474256	69474442
Gm49302	ENSMUST00000228127.1	-3,648451642	606	chr14	47801348	47801574
Gm49302	ENSMUST00000228127.1	-3,648451642	606	chr14	47801348	47801574
Rps14	ENSMUST00000236652.1	-3,644698547	9953	chr18	60736675	60737144
Tlr5	ENSMUST00000195614.1	-3,64118073	2511	chr1	1,83E+08	1,83E+08
Asap2	ENSMUST00000050990.9	-3,633464079	48494	chr12	21160450	21160828
4930471E1 9Rik	ENSMUST00000219022.1	-3,632925991	2948	chr10	1,21E+08	1,21E+08
Gm50064	ENSMUST00000234056.1	-3,631043232	13524	chr18	5334553	5334733
Gm42839	ENSMUST00000198978.1	-3,627524173	5108	chr5	1,25E+08	1,25E+08
Gm49766	ENSMUST00000232007.1	-3,62741244	0	chr10	67785457	67785983
Gm49766	ENSMUST00000232007.1	-3,62741244	0	chr10	67785457	67785983
Magix	ENSMUST00000130287.1	-3,625042109	602	chrX	7673768	7673961
Gm15496	ENSMUST00000129578.2	-3,622395658	23795	chr3	31261580	31261791
Rab8a	ENSMUST00000003121.8	-3,622191367	0	chr8	72160949	72161236
Gm45646	ENSMUST00000210983.1	-3,621655295	1134	chr8	26747065	26747468
Gm6063	ENSMUST00000206958.1	-3,620387661	8246	chr6	71632937	71633365
Plec	ENSMUST00000170728.7	-3,617766444	1388	chr15	76195783	76196608
Ddc	ENSMUST00000134401.1	-3,616756211	1198	chr11	11820551	11820919
Eif4g3	ENSMUST00000155142.1	-3,616467673	37721	chr4	1,38E+08	1,38E+08
Gm43221	ENSMUST00000197612.1	-3,61533328	10562	chr3	1,09E+08	1,09E+08
Gm16237	ENSMUST00000156666.1	-3,615158314	29275	chr8	1,25E+08	1,25E+08
Fbxw11	ENSMUST00000143290.1	-3,613731189	17478	chr11	32660384	32660587
Phospho1	ENSMUST00000150134.1	-3,611300696	23590	chr11	95800360	95800908
1700054A0 3Rik	ENSMUST00000235589.1	-3,609309937	6634	chr19	53040284	53040526
Grk6	ENSMUST00000224653.1	-3,609102166	0	chr13	55444959	55445215
Cd44	ENSMUST00000111192.2	-3,602094318	14004	chr2	1,03E+08	1,03E+08

Kcnn1	ENSMUST00000110078.4	-3,598113791	2046	chr8	70839655	70840002
Bmper	ENSMUST00000214050.1	-3,597435643	91144	chr9	23465078	23465494
Casz1	ENSMUST00000140617.1	-3,597424297	20985	chr4	1,49E+08	1,49E+08
Hsf2bp	ENSMUST00000138172.1	-3,596751518	28312	chr17	31982724	31982969
Gm26375	ENSMUST00000101829.2	-3,596462206	21445	chr17	13555511	13556123
Gm26375	ENSMUST00000101829.2	-3,596462206	21445	chr17	13555511	13556123
Lrch4	ENSMUST00000031734.15	-3,594743878	102	chr5	1,38E+08	1,38E+08
Gm30563	ENSMUST00000227720.1	-3,593327388	31437	chr15	64364043	64364941
Banp	ENSMUST00000173201.1	-3,593142616	110071	chr8	1,22E+08	1,22E+08
Gm26296	ENSMUST00000179457.1	-3,587737215	16622	chr15	55245483	55245919
Gm47175	ENSMUST00000216940.1	-3,585732192	40538	chr9	56463863	56464286
Pdlim7	ENSMUST00000153426.1	-3,585725703	1881	chr13	55510849	55511083
Rps13-ps6	ENSMUST00000120038.1	-3,585315521	2591	chr2	1,44E+08	1,44E+08
Tkt	ENSMUST00000223633.1	-3,585030776	1935	chr14	30561627	30561936
Gm15419	ENSMUST00000148654.1	-3,584440233	16440	chr8	11382356	11382745
Gm50212	ENSMUST00000236488.1	-3,581294356	2857	chr19	19246896	19247303
Mtmr7	ENSMUST00000174537.1	-3,575534643	11506	chr8	40595162	40595398
Traf1	ENSMUST00000135870.1	-3,575010326	118	chr2	34958272	34958644
Gm16046	ENSMUST00000128138.1	-3,57487401	3700	chr17	13683679	13684033
Gm27926	ENSMUST00000157180.2	-3,571484558	124355	chr15	53259349	53259563
Psd3	ENSMUST00000185416.2	-3,570789004	16884	chr8	68082258	68082569
Cpm	ENSMUST00000141991.1	-3,569429299	5906	chr10	1,18E+08	1,18E+08
AA986860	ENSMUST00000186185.1	-3,5651494	2528	chr1	1,31E+08	1,31E+08
Myocd	ENSMUST00000150823.1	-3,559007403	7870	chr11	65215386	65215863
Lax1	ENSMUST00000189524.1	-3,55561783	5387	chr1	1,34E+08	1,34E+08
Tmem220	ENSMUST00000061786.5	-3,55276465	6045	chr11	67031201	67031735
n-R5s47	ENSMUST00000122516.1	-3,551455764	10045	chr14	62879761	62880773
Gm15990	ENSMUST00000132223.1	-3,547479194	47684	chr10	86173513	86173856
Rhbdl2	ENSMUST00000148797.1	-3,546646417	639	chr4	1,24E+08	1,24E+08
Gmds	ENSMUST00000179717.1	-3,545665875	42256	chr13	32267490	32267843
Sox9	ENSMUST00000000579.2	-3,543770684	23674	chr11	1,13E+08	1,13E+08
Gm45819	ENSMUST00000210940.1	-3,5433563	10487	chr7	1,1E+08	1,1E+08
Ier5	ENSMUST00000055322.5	-3,543098856	13087	chr1	1,55E+08	1,55E+08
Cwc27	ENSMUST00000022228.12	-3,540712008	137589	chr13	1,04E+08	1,04E+08
Srrm2	ENSMUST00000233636.1	-3,532891952	0	chr17	23803035	23803406
Chchd3	ENSMUST00000115091.1	-3,531263952	84199	chr6	32935644	32935954
Gm44705	ENSMUST00000206719.1	-3,530340479	8783	chr7	79780782	79781503
Rai14	ENSMUST00000227366.1	-3,528705085	3239	chr15	10685841	10686051
6430562O15Rik	ENSMUST00000180491.1	-3,526441236	4964	chr13	99391490	99391959
Kcnn3	ENSMUST00000000811.7	-3,520893742	14501	chr3	89534667	89535066
Sik2	ENSMUST00000176824.7	-3,519725629	7397	chr9	50900200	50900677
Gng12	ENSMUST00000204862.1	-3,518867868	14019	chr6	66910912	66911130
Gm45781	ENSMUST00000212860.1	-3,517723463	16928	chr8	1,24E+08	1,24E+08
Capg	ENSMUST00000126124.7	-3,516625432	0	chr6	72547895	72548163
Gm45166	ENSMUST00000209073.1	-3,516555969	10879	chr8	23740853	23741203

A230083G16Rik	ENSMUST00000069553.2	-3,513724698	2603	chr6	1,25E+08	1,25E+08
A230083G16Rik	ENSMUST00000069553.2	-3,513724698	2603	chr6	1,25E+08	1,25E+08
Sap130	ENSMUST00000234846.1	-3,508708115	84	chr18	31634786	31635000
Fam83b	ENSMUST00000098546.3	-3,507848947	50141	chr9	76540848	76541186
Gm27931	ENSMUST00000184976.1	-3,506157609	4711	chr16	38202442	38202751
Pfkfb4	ENSMUST00000198140.4	-3,506080901	0	chr9	1,09E+08	1,09E+08
Lrmda	ENSMUST00000226003.1	-3,504480799	8402	chr14	22586982	22588110
Cavin1	ENSMUST00000132934.1	-3,504216544	1575	chr11	1,01E+08	1,01E+08
Abtb2	ENSMUST00000138580.1	-3,503499776	44499	chr2	1,04E+08	1,04E+08
Gm19692	ENSMUST00000203856.1	-3,496982242	7284	chr6	1,01E+08	1,01E+08
4933407I08Rik	ENSMUST00000151215.1	-3,496633219	57950	chr18	42876861	42877519
Gm25070	ENSMUST00000175237.1	-3,490402387	6834	chr15	95886552	95886854
Slc39a14	ENSMUST00000142598.1	-3,48594128	2409	chr14	70333488	70333700
7530414M10Rik	ENSMUST00000229317.1	-3,482731605	1104	chr15	83174302	83174718
7530414M10Rik	ENSMUST00000229317.1	-3,482731605	1104	chr15	83174302	83174718
Oit3	ENSMUST00000162493.1	-3,480011444	2578	chr10	59430873	59431052
Gm10811	ENSMUST00000221985.1	-3,478450215	0	chr13	13168587	13168987
1700015H07Rik	ENSMUST00000227291.1	-3,478204028	3285	chr15	53617851	53618369
Gm48969	ENSMUST00000227341.1	-3,47798515	13254	chr14	76874827	76875697
Anpep	ENSMUST00000149164.1	-3,476908639	7704	chr7	79850320	79850518
9830132P13Rik	ENSMUST00000198240.1	-3,476374458	13849	chr3	1,28E+08	1,28E+08
Rps15a-ps5	ENSMUST00000132586.1	-3,475436894	17393	chr5	1,06E+08	1,06E+08
Rbks	ENSMUST00000201744.1	-3,474918105	5657	chr5	31630098	31630285
Rbks	ENSMUST00000201744.1	-3,474918105	5657	chr5	31630098	31630285
Rbks	ENSMUST00000201744.1	-3,474918105	5657	chr5	31630098	31630285
Avpi1	ENSMUST00000161873.1	-3,470177285	4221	chr19	42129002	42129182
Gm24497	ENSMUST00000177868.1	-3,465806426	182	chr1	72244397	72245043
Gm24497	ENSMUST00000177868.1	-3,465806426	182	chr1	72244397	72245043
Gm26626	ENSMUST00000180989.3	-3,465348207	2960	chr16	92609315	92609853
Camta2	ENSMUST00000108544.7	-3,461044335	206	chr11	70668514	70669256
Bcl9l	ENSMUST00000218183.1	-3,459686063	1387	chr9	44488636	44488949
Tmem154	ENSMUST00000107682.1	-3,457700965	19605	chr3	84646250	84646586
Exosc7	ENSMUST00000161569.1	-3,456460927	1838	chr9	1,23E+08	1,23E+08
Gm17251	ENSMUST00000170103.2	-3,454408479	74	chr17	33760068	33760626
Gm17251	ENSMUST00000170103.2	-3,454408479	74	chr17	33760068	33760626
Gm36994	ENSMUST00000192941.1	-3,448878951	14540	chr13	1,01E+08	1,01E+08
Pptc7	ENSMUST00000053426.14	-3,448856851	4049	chr5	1,22E+08	1,22E+08
Sertad2	ENSMUST00000109586.2	-3,444811658	114	chr11	20631471	20631864
Gm25939	ENSMUST00000179876.1	-3,444150218	285	chr1	72255295	72256221
Rps19	ENSMUST00000108430.9	-3,443156123	0	chr7	24884095	24884424
Slc12a8	ENSMUST00000122314.7	-3,442233738	1858	chr16	33594260	33594703
Sfr1	ENSMUST00000099353.5	-3,441590576	0	chr19	47731216	47731699
Gm17268	ENSMUST00000171515.1	-3,440917242	16143	chr11	82011611	82012080

Lmntd1	ENSMUST00000111708.8	-3,439863248	3902	chr6	1,45E+08	1,45E+08
Lmntd1	ENSMUST00000111708.8	-3,439863248	3902	chr6	1,45E+08	1,45E+08
Pmepa1os	ENSMUST00000150185.1	-3,438030281	9852	chr2	1,73E+08	1,73E+08
Crybg1	ENSMUST00000239010.1	-3,437086893	35084	chr10	44039261	44039728
Pnrc1	ENSMUST00000137589.1	-3,43615952	15322	chr4	33261336	33261753
Sh3rf2	ENSMUST00000074679.3	-3,436090456	6181	chr18	42059893	42060109
Ccdc162	ENSMUST00000160751.2	-3,435947992	7047	chr10	41587274	41587469
Prdm11	ENSMUST00000111274.8	-3,429097939	23673	chr2	92948059	92948344
Nr1d1	ENSMUST00000139220.1	-3,425914748	5944	chr11	98775293	98775908
Gm13816	ENSMUST00000152230.1	-3,424174385	8632	chr2	92750864	92751383
1700126H18Rik	ENSMUST00000201903.1	-3,422045833	262	chr5	66165957	66166344
1700126H18Rik	ENSMUST00000201903.1	-3,422045833	262	chr5	66165957	66166344
Deptor	ENSMUST00000226687.1	-3,421650269	1414	chr15	55134869	55135122
Tmcc3	ENSMUST00000121471.7	-3,420306418	12	chr10	94574967	94575244
Tmcc3	ENSMUST00000121471.7	-3,420306418	12	chr10	94574967	94575244
Lamc2	ENSMUST00000027753.12	-3,418723551	0	chr1	1,53E+08	1,53E+08
Capn5	ENSMUST00000040971.13	-3,414947806	4160	chr7	98117213	98117398
Gipc2	ENSMUST00000197813.1	-3,4133924	407	chr3	1,52E+08	1,52E+08
Angptl4	ENSMUST00000174872.1	-3,412746052	2417	chr17	33779431	33779901
Pik3r5	ENSMUST00000126876.7	-3,408448624	5504	chr11	68467553	68468290
Grhl2	ENSMUST00000161933.1	-3,40570102	138	chr15	37317870	37318264
Tmem211	ENSMUST00000086615.2	-3,404308535	10185	chr5	1,13E+08	1,13E+08
Gm36677	ENSMUST00000229684.1	-3,403727072	98229	chr15	59520164	59520360
Gm37053	ENSMUST00000192399.1	-3,40169946	17650	chr1	1,07E+08	1,07E+08
Cflar	ENSMUST00000114309.7	-3,401544763	583	chr1	58712456	58712710
Bhlhe40	ENSMUST00000032194.10	-3,400016962	130	chr6	1,09E+08	1,09E+08
Ttc28	ENSMUST00000129017.1	-3,396403226	12464	chr5	1,11E+08	1,11E+08
Fbxl13	ENSMUST00000137788.7	-3,388448715	1914	chr5	21492116	21492378
Fbn1	ENSMUST00000103234.1	-3,38767671	1573	chr2	1,25E+08	1,25E+08
Prkcz	ENSMUST00000123652.7	-3,386577463	8662	chr4	1,55E+08	1,55E+08
Gm43430	ENSMUST00000196513.1	-3,38650738	24212	chr3	1,36E+08	1,36E+08
Ptprs	ENSMUST00000224193.1	-3,384461909	4744	chr17	56462361	56462640
4833419O12Rik	ENSMUST00000231314.1	-3,382972914	0	chr16	20800085	20800388
Gm19276	ENSMUST00000228418.1	-3,382896617	3436	chr15	10818265	10818535
Gm16537	ENSMUST00000161529.1	-3,38273016	60	chr15	99715605	99716268
Nemp1	ENSMUST00000142580.1	-3,382613365	749	chr10	1,28E+08	1,28E+08
Nemp1	ENSMUST00000142580.1	-3,382613365	749	chr10	1,28E+08	1,28E+08
Gm44013	ENSMUST00000204392.1	-3,381549075	593	chr6	1,41E+08	1,41E+08
Ppp1r10	ENSMUST00000130124.1	-3,381235619	0	chr17	35916489	35916730
Gm30726	ENSMUST00000232268.1	-3,380939405	48671	chr16	76499788	76500123
Megf10	ENSMUST00000139892.1	-3,380479212	24993	chr18	57167781	57167977
Gramd2	ENSMUST00000123914.7	-3,379698103	11596	chr9	59691742	59692427
Gm32025	ENSMUST00000217960.1	-3,373215733	780	chr10	71147180	71147379
Gm44893	ENSMUST00000207259.1	-3,3722647	17676	chr7	1,32E+08	1,32E+08

Gm22518	ENSMUST00000158267.1	-3,370363151	10643	chr4	45489296	45489841
Gm26805	ENSMUST00000181885.1	-3,368621224	214	chr1	37864890	37865084
Lamc3	ENSMUST00000135995.1	-3,366388102	9686	chr2	31934745	31935274
Oard1	ENSMUST00000167180.7	-3,364264735	0	chr17	48409693	48410044
Oard1	ENSMUST00000167180.7	-3,364264735	0	chr17	48409693	48410044
Rnf220	ENSMUST00000138435.1	-3,361276184	5964	chr4	1,17E+08	1,17E+08
Gm39164	ENSMUST00000211519.1	-3,35911836	210	chr8	36175220	36175835
Zbtb44	ENSMUST00000216649.1	-3,35911778	20304	chr9	31009813	31010339
St5	ENSMUST00000207745.1	-3,358313605	6284	chr7	1,1E+08	1,1E+08
Eml1	ENSMUST00000054955.13	-3,354385283	16399	chr12	1,08E+08	1,08E+08
Actb	ENSMUST00000164765.1	-3,352086301	1013	chr5	1,43E+08	1,43E+08
Gm25313	ENSMUST00000101817.1	-3,351755872	72	chr9	65195725	65196499
Gramd3	ENSMUST00000237716.1	-3,346554774	7932	chr18	56477201	56477414
Parva	ENSMUST00000106640.1	-3,339463647	27804	chr7	1,12E+08	1,12E+08
Mindy1	ENSMUST00000133762.1	-3,339379532	1617	chr3	95293605	95293868
Gm3513	ENSMUST00000161319.1	-3,335501947	7987	chr3	79559303	79559550
Gm27203	ENSMUST00000183677.1	-3,331349318	10765	chr9	72838024	72838510
Acot7	ENSMUST00000124548.7	-3,328343322	0	chr4	1,52E+08	1,52E+08
Gm34636	ENSMUST00000235198.1	-3,325784313	4652	chr19	43551071	43551274
Gm34636	ENSMUST00000235198.1	-3,325784313	4652	chr19	43551071	43551274
Ptgfrn	ENSMUST00000102694.3	-3,32158922	7594	chr3	1,01E+08	1,01E+08
2810006K23Rik	ENSMUST00000111477.1	-3,320909168	236	chr5	1,24E+08	1,24E+08
Tcf7l2	ENSMUST00000111662.10	-3,32056364	170714	chr19	55570634	55571105
Gm49211	ENSMUST00000226266.1	-3,320366634	0	chr15	56003943	56004193
Gm23513	ENSMUST00000158181.2	-3,320200449	25458	chr16	52079083	52079556
1700110K17Rik	ENSMUST00000140807.8	-3,313671698	1726	chr9	40321497	40321699
P2ry14	ENSMUST00000197841.1	-3,313496639	276	chr3	59114133	59114335
P2ry14	ENSMUST00000197841.1	-3,313496639	276	chr3	59114133	59114335
Mpp2	ENSMUST00000132687.1	-3,311034277	6511	chr11	1,02E+08	1,02E+08
Gm28096	ENSMUST00000191391.1	-3,309238847	9708	chr4	95161450	95161814
Gm28644	ENSMUST00000191364.1	-3,306266274	17147	chr1	31059068	31059299
Gm13833	ENSMUST00000146766.1	-3,305792295	1996	chr6	31100303	31100849
Agfg2	ENSMUST00000138604.1	-3,303944845	8713	chr5	1,38E+08	1,38E+08
Ddi1	ENSMUST00000051706.5	-3,301555581	80066	chr9	6342801	6342988
Gm13216	ENSMUST00000117145.1	-3,298138508	10413	chr2	5613986	5614239
Gm14508	ENSMUST00000127049.1	-3,297996339	246	chr5	1,16E+08	1,16E+08
Zfp407	ENSMUST00000182849.1	-3,296057072	16143	chr18	84413526	84413843
A530053M12Rik	ENSMUST00000187918.1	-3,295646811	901	chr1	1,06E+08	1,06E+08
Gm22083	ENSMUST00000157586.1	-3,294484379	965	chrX	8084959	8085258
Gm41724	ENSMUST00000235622.1	-3,293398325	6881	chr18	52535544	52535839
Mgat4c	ENSMUST00000020039.12	-3,292939564	15532	chr10	1,02E+08	1,02E+08
Gm15169	ENSMUST00000130794.1	-3,28917575	9104	chrX	1,58E+08	1,58E+08
Anxa8	ENSMUST00000022519.14	-3,287036865	8	chr14	34085499	34085972
Slc25a25	ENSMUST00000194756.1	-3,284175292	1091	chr2	32428442	32428720

Mir26b	ENSMUST00000083534.1	-3,284049915	2962	chr1	74397274	74397518
Wdr70	ENSMUST00000045766.7	-3,283917449	4843	chr15	7877907	7878283
Prl	ENSMUST00000110369.9	-3,280140669	29796	chr13	27027523	27027773
Samd8	ENSMUST00000022292.9	-3,278885146	2496	chr14	21747830	21748034
Rpl21-ps8	ENSMUST00000130005.2	-3,276282219	418	chr18	82522594	82523120
Ensa	ENSMUST00000197050.1	-3,274979414	0	chr3	95624874	95625253
Mllt3	ENSMUST00000128930.7	-3,265333124	7670	chr4	87799525	87799903
Fam129b	ENSMUST00000150875.1	-3,265096771	1082	chr2	32917354	32917558
Hsd17b12	ENSMUST00000146580.1	-3,263777985	11863	chr2	94045387	94045629
Tsc2	ENSMUST00000227607.1	-3,261994744	662	chr17	24600274	24600544
Siva1	ENSMUST00000021728.11	-3,260464252	0	chr12	1,13E+08	1,13E+08
Gm41495	ENSMUST00000232211.1	-3,256755045	9440	chr16	92163689	92164075
Gm41495	ENSMUST00000232211.1	-3,256755045	9440	chr16	92163689	92164075
BC048644	ENSMUST00000181944.1	-3,25581857	9270	chr8	1,22E+08	1,22E+08
BC048644	ENSMUST00000181944.1	-3,25581857	9270	chr8	1,22E+08	1,22E+08
Slc34a2	ENSMUST00000170523.7	-3,251213106	4239	chr5	53042322	53042664
Lhfpl2	ENSMUST00000121618.1	-3,250021278	10861	chr13	94184855	94185088
Gm43128	ENSMUST00000202212.1	-3,249951589	9558	chr2	35270629	35270831
Pde4b	ENSMUST00000097950.8	-3,249379574	0	chr4	1,03E+08	1,03E+08
Atg7	ENSMUST00000182771.3	-3,24474653	2228	chr6	1,15E+08	1,15E+08
Ablim1	ENSMUST00000134430.7	-3,244494163	2020	chr19	57079324	57079530
Acp5	ENSMUST00000216684.1	-3,242737673	4632	chr9	22137254	22137763
Clec2g	ENSMUST00000203405.2	-3,242185006	416	chr6	1,29E+08	1,29E+08
Clec2g	ENSMUST00000203405.2	-3,242185006	416	chr6	1,29E+08	1,29E+08
Vmp1	ENSMUST00000153971.1	-3,237894944	1804	chr11	86587078	86587465
Zmynd12	ENSMUST00000134147.1	-3,229048185	2655	chr4	1,19E+08	1,19E+08
5430403N17Rik	ENSMUST00000210717.1	-3,228487855	1896	chr8	35865983	35866168
Morrbid	ENSMUST00000225087.1	-3,225901363	1478	chr2	1,28E+08	1,28E+08
Gm45941	ENSMUST00000223144.1	-3,224878653	2414	chr12	27977946	27978182
Ston2	ENSMUST00000166967.1	-3,222597072	4729	chr12	91748349	91748698
Nln	ENSMUST00000224475.1	-3,216871134	1497	chr13	1,04E+08	1,04E+08
Gm45366	ENSMUST00000209828.1	-3,216654328	53688	chr8	56070407	56070969
Mical3	ENSMUST00000207993.1	-3,216432339	1657	chr6	1,21E+08	1,21E+08
Gm28981	ENSMUST00000190863.1	-3,213790607	40150	chr1	64226143	64226549
Chp1	ENSMUST00000154406.7	-3,212794016	4494	chr2	1,2E+08	1,2E+08
Morf4l1-ps1	ENSMUST00000231737.1	-3,208856977	30855	chr16	24498344	24498538
Pakap	ENSMUST00000238994.1	-3,207712391	15962	chr4	57804856	57805087
Pakap	ENSMUST00000238994.1	-3,207712391	15962	chr4	57804856	57805087
Gm36970	ENSMUST00000193688.1	-3,207364007	1169	chr1	90838446	90838675
Gm36970	ENSMUST00000193688.1	-3,207364007	1169	chr1	90838446	90838675
Camk2d	ENSMUST00000198637.1	-3,203120442	24385	chr3	1,27E+08	1,27E+08
Ttll1	ENSMUST00000016897.11	-3,199271339	32003	chr15	83451552	83451765
Fgf7	ENSMUST00000138576.1	-3,198839446	2996	chr2	1,26E+08	1,26E+08
Gm35360	ENSMUST00000227155.1	-3,194657753	0	chr14	47805181	47805472
Gm35360	ENSMUST00000227155.1	-3,194657753	0	chr14	47805181	47805472

Gm36026	ENSMUST00000230247.1	-3,193882285	2774	chr15	1,02E+08	1,02E+08
4930486F22 Rik	ENSMUST00000218077.1	-3,190553329	4618	chr10	86100479	86100944
4930486F22 Rik	ENSMUST00000218077.1	-3,190553329	4618	chr10	86100479	86100944
Nbr1	ENSMUST00000147239.7	-3,188731542	0	chr11	1,02E+08	1,02E+08
Ido1	ENSMUST00000033956.6	-3,186151774	10734	chr8	24573216	24573401
Gm14012	ENSMUST00000136025.1	-3,183519792	7611	chr2	1,28E+08	1,28E+08
Fermt1	ENSMUST00000144342.1	-3,182258615	13541	chr2	1,33E+08	1,33E+08
Lrp8	ENSMUST00000146552.8	-3,179967961	758	chr4	1,08E+08	1,08E+08
Fstl1	ENSMUST00000135668.7	-3,178986082	11075	chr16	37788229	37788626
Nup62	ENSMUST00000057195.16	-3,176575632	0	chr7	44815818	44816164
Nup62	ENSMUST00000057195.16	-3,176575632	0	chr7	44815818	44816164
Ano6	ENSMUST00000226932.1	-3,176538786	3716	chr15	95794728	95794932
Atl1	ENSMUST00000222141.1	-3,17389227	7367	chr12	69885065	69885246
Rnu3b3	ENSMUST00000093738.2	-3,171226015	306	chr11	87448935	87449413
Elmo1	ENSMUST00000180626.1	-3,168420746	3936	chr13	20115450	20115689
Foxn1	ENSMUST00000108294.1	-3,168362886	5612	chr11	78363191	78363758
Nop10	ENSMUST00000028553.3	-3,162354756	6678	chr2	1,12E+08	1,12E+08
Erlin1	ENSMUST00000112028.9	-3,159403082	11458	chr19	44023239	44023485
Src	ENSMUST00000109531.7	-3,156509764	7730	chr2	1,57E+08	1,57E+08
Hnf1b	ENSMUST00000108113.2	-3,154529677	43714	chr11	83896676	83896908
BC037704	ENSMUST00000238165.1	-3,152524331	0	chr19	43675003	43675309
Gm7775	ENSMUST00000218871.1	-3,152492336	6455	chr10	77129962	77130644
Arhgef10	ENSMUST00000162444.1	-3,152247968	1872	chr8	14992233	14992485
Gm12530	ENSMUST00000152074.1	-3,151512355	31502	chr4	57139455	57140568
Dlx4os	ENSMUST00000156477.3	-3,149987466	12634	chr11	95157688	95158116
Gm15506	ENSMUST00000208646.1	-3,149155369	4684	chr7	98694253	98694540
Gm44361	ENSMUST00000197960.1	-3,147696749	41030	chr3	1,34E+08	1,34E+08
Cald1	ENSMUST00000127055.1	-3,14490459	11658	chr6	34610280	34610646
Arhgdia	ENSMUST00000106197.9	-3,143440169	4769	chr11	1,21E+08	1,21E+08
9130015A2 1Rik	ENSMUST00000222153.1	-3,139824649	24	chr12	35672406	35672672
9130015A2 1Rik	ENSMUST00000222153.1	-3,139824649	24	chr12	35672406	35672672
1700011C1 1Rik	ENSMUST00000205268.1	-3,134502331	89	chr7	71960835	71961083
Tmem45a	ENSMUST00000135672.1	-3,131454404	24785	chr16	56850501	56850868
Gm35721	ENSMUST00000220294.1	-3,125458289	61439	chr10	76902715	76903213
Galnt9	ENSMUST00000040001.13	-3,121676394	20856	chr5	1,11E+08	1,11E+08
Nedd4l	ENSMUST00000237644.1	-3,11746284	4341	chr18	65169530	65169725
Gm2553	ENSMUST00000215986.1	-3,117154518	54659	chr9	63745969	63746533
Setd5	ENSMUST00000143998.7	-3,11464643	145	chr6	1,13E+08	1,13E+08
2210409D0 7Rik	ENSMUST00000236285.1	-3,114453868	8271	chr18	57623552	57623807
Sgms1	ENSMUST00000141252.1	-3,114430018	15684	chr19	32304965	32305152
Mrpl33	ENSMUST00000031024.13	-3,109496347	7475	chr5	31606269	31606463
Mrpl33	ENSMUST00000031024.13	-3,109496347	7475	chr5	31606269	31606463
Gm44792	ENSMUST00000207008.1	-3,10921723	10931	chr7	65468444	65468662

Mxi1	ENSMUST00000237480.1	-3,10669173	918	chr19	53313986	53314612
Sap30bpos	ENSMUST00000132264.1	-3,106460799	8481	chr11	1,16E+08	1,16E+08
Junb	ENSMUST00000064922.6	-3,105941716	292	chr8	84976363	84976617
Hdgf	ENSMUST00000160198.1	-3,104838706	598	chr3	87910634	87911001
Gm973	ENSMUST00000212835.1	-3,103579621	3072	chr1	59578750	59579323
Gm4828	ENSMUST00000231980.1	-3,103393729	12092	chr17	4868325	4868533
Myzap	ENSMUST00000166112.1	-3,097145238	3602	chr9	71568096	71568474
Zscan2	ENSMUST00000155128.2	-3,096871992	3797	chr7	80866695	80866887
Trim8	ENSMUST00000026008.8	-3,092360686	2137	chr19	46503841	46504306
Adgre5	ENSMUST00000149368.1	-3,091925528	4509	chr8	83738045	83738375
5430402P08 Rik	ENSMUST00000211799.1	-3,091623247	908	chr7	1,11E+08	1,11E+08
0610009E0 2Rik	ENSMUST00000133463.1	-3,091347432	0	chr2	26445448	26445873
Gm15402	ENSMUST00000114051.2	-3,089207575	12190	chr6	72711406	72712018
Ing1	ENSMUST00000054399.5	-3,088536723	0	chr8	11555455	11556099
Gm14011	ENSMUST00000145178.1	-3,088525095	12115	chr2	1,29E+08	1,29E+08
Gm44835	ENSMUST00000205544.1	-3,088320689	14630	chr7	75575246	75575510
Gm16350	ENSMUST00000162958.1	-3,083940898	4366	chr8	14965434	14965835
4930580E0 4Rik	ENSMUST00000197377.1	-3,083454672	0	chr5	23387021	23387265
4930580E0 4Rik	ENSMUST00000197377.1	-3,083454672	0	chr5	23387021	23387265
Gm16096	ENSMUST00000132716.1	-3,083359376	737	chr9	40781487	40781713
Gm16096	ENSMUST00000132716.1	-3,083359376	737	chr9	40781487	40781713
Upp2	ENSMUST00000135737.1	-3,081767495	20543	chr2	58546558	58546754
Tlcd1	ENSMUST00000147819.7	-3,081496062	315	chr11	78177631	78177814
Zdhhc1	ENSMUST00000044286.5	-3,080341582	1673	chr8	1,05E+08	1,05E+08
Opa3	ENSMUST00000161711.1	-3,07993964	0	chr7	19228049	19228835
Acsbg1	ENSMUST00000132914.1	-3,074856608	3268	chr9	54642607	54642914
Gm6327	ENSMUST00000171185.2	-3,073606731	16735	chr16	12742756	12743296
Tafa5	ENSMUST00000068088.7	-3,071972632	29148	chr15	87654380	87654855
Gm45555	ENSMUST00000210028.1	-3,070968718	8750	chr8	22882341	22882876
Gm27151	ENSMUST00000183473.1	-3,070499396	94212	chr19	22343824	22344307
Cst6	ENSMUST00000025764.5	-3,067646521	855	chr19	5345562	5345965
Gm50372	ENSMUST00000236726.1	-3,066917782	6038	chr19	4673872	4674076
C530044C1 6Rik	ENSMUST00000204275.1	-3,065912725	815	chr6	50777141	50777430
Mannr	ENSMUST00000193549.1	-3,065902477	10681	chr3	29879768	29880332
Adgrg1	ENSMUST00000093271.7	-3,060059737	184	chr8	94983995	94984186
4930554I06 Rik	ENSMUST00000237768.1	-3,057363558	0	chr19	21104553	21105163
Gm12796	ENSMUST00000131864.1	-3,056691558	14081	chr4	1,01E+08	1,01E+08
Slc43a2	ENSMUST00000145901.7	-3,055597636	1367	chr11	75564220	75564639
Hspa12a	ENSMUST00000236839.1	-3,053405304	25512	chr19	58853070	58853381
Otop2	ENSMUST00000106544.1	-3,049966871	3731	chr11	1,15E+08	1,15E+08
Gm43707	ENSMUST00000196451.1	-3,046640118	3026	chr3	1,44E+08	1,44E+08
Gm43707	ENSMUST00000196451.1	-3,046640118	3026	chr3	1,44E+08	1,44E+08
Rubcn	ENSMUST00000149621.1	-3,044160862	2052	chr16	32859076	32859407

Vps36	ENSMUST00000153949.1	-3,043420322	1702	chr8	22215064	22215290
Rpl31-ps13	ENSMUST00000060994.5	-3,041703341	3829	chr13	1E+08	1E+08
Gm572	ENSMUST00000105698.2	-3,039706885	3432	chr4	1,49E+08	1,49E+08
Kank1	ENSMUST00000155788.1	-3,038322821	6183	chr19	25428424	25428746
Cic	ENSMUST00000005578.12	-3,036383519	0	chr7	25281932	25282428
Dock4	ENSMUST00000220912.1	-3,032495523	92459	chr12	40538797	40539033
Rnf40	ENSMUST00000205694.1	-3,032265509	220	chr7	1,28E+08	1,28E+08
Gm43323	ENSMUST00000202328.1	-3,02956474	17892	chr5	66098103	66098846
Gm17227	ENSMUST00000166590.1	-3,028071438	4390	chr19	7302126	7302417
Gm17227	ENSMUST00000166590.1	-3,028071438	4390	chr19	7302126	7302417
Ints6	ENSMUST00000225193.1	-3,02697174	3638	chr14	62761253	62761501
Ell	ENSMUST00000210155.1	-3,026197594	0	chr8	70538912	70539589
P2ry10b	ENSMUST00000067249.2	-3,025224707	1322	chrX	1,07E+08	1,07E+08
Cox14	ENSMUST00000023761.3	-3,024845669	794	chr15	99724642	99724852
Krt78	ENSMUST00000164932.2	-3,018101364	6579	chr15	1,02E+08	1,02E+08
Rnu7	ENSMUST00000158458.1	-3,013968362	0	chr6	1,25E+08	1,25E+08
Rnu7	ENSMUST00000158458.1	-3,013968362	0	chr6	1,25E+08	1,25E+08
Rnu7	ENSMUST00000158458.1	-3,013968362	0	chr6	1,25E+08	1,25E+08
Itpripl1	ENSMUST00000132773.1	-3,008850878	477	chr2	1,27E+08	1,27E+08
Dync1i1	ENSMUST00000203520.1	-3,007747137	13598	chr6	5991952	5992467
Gm13328	ENSMUST00000120816.1	-3,005118413	66620	chr2	20167508	20168424
Gm10097	ENSMUST00000238588.1	-3,002775859	1916	chr10	5071113	5071343
Gm10097	ENSMUST00000238588.1	-3,002775859	1916	chr10	5071113	5071343
Lhfp	ENSMUST00000196913.1	-2,99842999	33301	chr3	53195817	53196023
Tec	ENSMUST00000202547.1	-2,997067581	31965	chr5	72855429	72856030
Synj2	ENSMUST00000115784.7	-2,99310891	1209	chr17	6006181	6006370
Sp4	ENSMUST00000221844.1	-2,991229787	39288	chr12	1,18E+08	1,18E+08
Eps8	ENSMUST00000134630.5	-2,989358572	0	chr6	1,38E+08	1,38E+08
Itgb3	ENSMUST00000127140.2	-2,988882115	12236	chr11	1,05E+08	1,05E+08
Scd1	ENSMUST00000235741.1	-2,982135594	1185	chr19	44407872	44408427
Magi1	ENSMUST00000203034.1	-2,977872152	92441	chr6	94225366	94225598
Utrn	ENSMUST00000219660.1	-2,976261608	25771	chr10	12694761	12695053
Tnrc6a	ENSMUST00000206458.1	-2,974465045	13383	chr7	1,23E+08	1,23E+08
Gm22532	ENSMUST00000158822.1	-2,974260906	39565	chr8	73135296	73135553
Tmem69	ENSMUST00000106478.8	-2,97029969	14804	chr4	1,17E+08	1,17E+08
Heca	ENSMUST00000218268.1	-2,967626507	23762	chr10	17926050	17926345
Gm37113	ENSMUST00000195675.1	-2,965327435	4788	chr9	98498758	98498974
Gm19798	ENSMUST00000201502.1	-2,960049736	16488	chr5	34077251	34077477
Gm44127	ENSMUST00000203565.1	-2,957379941	46630	chr6	84271884	84272429
Gm29487	ENSMUST00000190146.1	-2,950670172	13752	chr1	1,31E+08	1,31E+08
Arrb1	ENSMUST00000162043.7	-2,949239365	7684	chr7	99586080	99586908
Gm43066	ENSMUST00000199589.1	-2,948826984	401	chr3	1,04E+08	1,04E+08
Gm48646	ENSMUST00000214468.1	-2,943906581	15281	chr9	22385835	22386113
Laptm4a	ENSMUST00000020909.3	-2,936931577	0	chr12	8921637	8921878
Bco1	ENSMUST00000034308.14	-2,933886257	21114	chr8	1,17E+08	1,17E+08
Nmnat2	ENSMUST00000190960.1	-2,931585338	1121	chr1	1,53E+08	1,53E+08

Birc6	ENSMUST00000180037.7	-2,93135862	0	chr17	74528042	74528310
Birc6	ENSMUST00000180037.7	-2,93135862	0	chr17	74528042	74528310
Gm42809	ENSMUST00000198429.1	-2,929076535	16069	chr3	89896570	89896846
Gm47014	ENSMUST00000225869.1	-2,924462093	20126	chr13	1,03E+08	1,03E+08
Gm16191	ENSMUST00000148464.1	-2,92355684	0	chr17	28925165	28925494
Gm36199	ENSMUST00000234384.1	-2,922997734	5156	chr17	29440020	29440220
Ier2	ENSMUST00000060427.5	-2,92209896	29	chr8	84661362	84661630
Casq2	ENSMUST00000164123.1	-2,920497297	17352	chr3	1,02E+08	1,02E+08
4933413L06Rik	ENSMUST00000111588.8	-2,918980998	47334	chr13	1,18E+08	1,18E+08
Neat1	ENSMUST00000174287.1	-2,913107084	327	chr19	5842636	5842914
Rasgef1b	ENSMUST00000146396.7	-2,908564949	31775	chr5	99329021	99329247
Smad3	ENSMUST00000137065.1	-2,90013452	10168	chr9	63677983	63678277
Kctd10	ENSMUST00000123538.1	-2,899174701	5519	chr5	1,14E+08	1,14E+08
Fmnl2	ENSMUST00000050719.12	-2,898416553	72594	chr2	52930464	52930672
Gm45667	ENSMUST00000210380.1	-2,897168585	53	chr7	1,05E+08	1,05E+08
Gm45667	ENSMUST00000210380.1	-2,897168585	53	chr7	1,05E+08	1,05E+08
Gm27813	ENSMUST00000184343.1	-2,893885331	11122	chr6	1,08E+08	1,08E+08
Gm42751	ENSMUST00000198065.1	-2,891458119	3868	chr3	37978703	37978923
Ano1	ENSMUST00000131731.1	-2,888701766	29106	chr7	1,45E+08	1,45E+08
Hmgcll1	ENSMUST00000008052.12	-2,88059496	11301	chr9	76003373	76003553
1110002E22Rik	ENSMUST00000163080.2	-2,879774398	3377	chr3	1,38E+08	1,38E+08
Gm47468	ENSMUST00000215971.1	-2,87554497	20918	chr9	1,03E+08	1,03E+08
Frmd6	ENSMUST00000057859.8	-2,871620749	30056	chr12	70795179	70795457
Lancl3	ENSMUST00000069763.2	-2,864331824	22860	chrX	9222764	9223041
Mir5123	ENSMUST00000177794.1	-2,863981264	12297	chr4	40837540	40837758
4921509A06Rik	ENSMUST00000171278.1	-2,860898566	5774	chr9	78623370	78623599
Hrh1	ENSMUST00000161220.1	-2,860572763	101	chr6	1,14E+08	1,14E+08
Ifnk	ENSMUST00000058595.6	-2,857380506	5297	chr4	35146553	35146758
Gm15572	ENSMUST00000119248.1	-2,850756742	17075	chr6	52825845	52826033
Rnu1a1	ENSMUST00000093684.1	-2,848960545	330	chr11	87423199	87423538
Mgat4b	ENSMUST00000147468.7	-2,847111431	2097	chr11	50212989	50213503
Gm7473	ENSMUST00000224513.1	-2,846804518	40384	chr14	22815404	22815962
Gm3470	ENSMUST00000160819.1	-2,844235454	10867	chr5	99010494	99010935
Cep295nl	ENSMUST00000103024.3	-2,843224571	4713	chr11	1,18E+08	1,18E+08
Gm39384	ENSMUST00000213703.1	-2,837786105	7338	chr9	83564042	83564221
Coro6	ENSMUST00000079770.2	-2,828861548	785	chr11	77464698	77465270
Ing1	ENSMUST00000210041.1	-2,824348804	0	chr8	11558095	11558388
Gm11266	ENSMUST00000139835.1	-2,819443994	3039	chr4	82511057	82511437
Gm11266	ENSMUST00000139835.1	-2,819443994	3039	chr4	82511057	82511437
Hdac8	ENSMUST00000154872.7	-2,818135056	21747	chrX	1,02E+08	1,02E+08
4933402D24Rik	ENSMUST00000027102.1	-2,817965619	11439	chr1	63766348	63766554
4732471J01Rik	ENSMUST00000205787.1	-2,811933738	0	chr7	25376782	25377021
3110001I22Rik	ENSMUST00000223666.1	-2,808805843	1818	chr16	13678859	13679187

Gm16253	ENSMUST00000148290.1	-2,80729223	0	chr3	96576234	96577199
Dhx34	ENSMUST00000133518.1	-2,802404489	2684	chr7	16208675	16209174
Gm44105	ENSMUST00000204401.1	-2,800719424	2638	chr6	90796536	90796972
1700086D15Rik	ENSMUST00000108696.6	-2,799222368	22671	chr11	65128615	65129238
2700054A10Rik	ENSMUST00000185626.6	-2,798686852	3594	chr17	13492869	13493057
2700054A10Rik	ENSMUST00000185626.6	-2,798686852	3594	chr17	13492869	13493057
Tbc1d1	ENSMUST00000147348.5	-2,797259464	4575	chr5	64279997	64280423
4930599A14Rik	ENSMUST00000186724.1	-2,797245535	115823	chr1	1,27E+08	1,27E+08
4632428C04Rik	ENSMUST00000181485.2	-2,794982743	12	chr16	30008681	30009092
Hs3st3a1	ENSMUST00000058652.5	-2,793955462	67450	chr11	64502784	64503259
Eif5a	ENSMUST00000152589.1	-2,789206459	3967	chr11	69921426	69921661
Cdk2ap2	ENSMUST00000174799.1	-2,785520173	0	chr19	4097270	4097557
Gsr	ENSMUST00000149528.1	-2,784283593	5346	chr8	33674350	33674646
Rad51b	ENSMUST00000221257.1	-2,778901898	25008	chr12	79352363	79353006
Ralgds	ENSMUST00000238699.1	-2,778541403	2088	chr2	28525504	28525761
Myl1	ENSMUST00000186346.1	-2,778447096	1670	chr1	66931864	66932070
Gm45185	ENSMUST00000207556.1	-2,771308054	0	chr7	99141568	99141788
Ehbp1	ENSMUST00000137594.1	-2,770982742	55190	chr11	22287194	22287710
Specc1	ENSMUST00000201624.3	-2,768396393	70	chr11	62005422	62005660
Adamts6	ENSMUST00000224504.1	-2,762488371	160588	chr13	1,04E+08	1,04E+08
Ptprq	ENSMUST00000218399.1	-2,745160403	17084	chr10	1,08E+08	1,08E+08
Thsd4	ENSMUST00000146099.1	-2,74500928	4856	chr9	60504655	60505018
Rdh8	ENSMUST00000066387.5	-2,744811918	5455	chr9	20812534	20813047
Hip1r	ENSMUST00000167879.1	-2,742460919	962	chr5	1,24E+08	1,24E+08
Sytl2	ENSMUST00000207455.1	-2,737477203	5116	chr7	90370445	90370668
Junos	ENSMUST00000125917.1	-2,730616596	311	chr4	95052283	95052639
Galk2	ENSMUST00000110454.1	-2,724305415	56576	chr2	1,26E+08	1,26E+08
Sorcs2	ENSMUST00000135324.7	-2,720113865	10040	chr5	36075418	36075645
Apbb2	ENSMUST00000159847.1	-2,715703083	9525	chr5	66550229	66550712
Fmnl1	ENSMUST00000107027.8	-2,706926469	3182	chr11	1,03E+08	1,03E+08
Rnu3b2	ENSMUST00000196353.1	-2,704855107	727	chr11	87472097	87472884
Gm29641	ENSMUST00000188955.1	-2,69780401	3578	chr1	62091314	62091502
Atoh8	ENSMUST00000206553.1	-2,697712778	6773	chr6	72216618	72216968
Lpar3	ENSMUST00000039164.3	-2,692608077	30708	chr3	1,46E+08	1,46E+08
Gm15809	ENSMUST00000159361.1	-2,691243798	7863	chr13	42990306	42990647
Has2os	ENSMUST00000165880.1	-2,686361512	1	chr15	56764908	56765458
Gm31105	ENSMUST00000211421.1	-2,684666098	21713	chr8	80857990	80858226
Gm47851	ENSMUST00000224348.1	-2,678245716	1712	chr13	1,04E+08	1,04E+08
Tatdn2	ENSMUST00000089018.10	-2,676198418	0	chr6	1,14E+08	1,14E+08
Pvt1	ENSMUST00000182141.1	-2,673897182	64647	chr15	62287252	62287472
Gm37101	ENSMUST00000191656.1	-2,672866993	28548	chr1	1,39E+08	1,39E+08
Vim	ENSMUST00000028062.7	-2,661992954	0	chr2	13573309	13574147
Fam149b	ENSMUST00000224930.1	-2,661736696	1974	chr14	20346002	20346187
Abcb8	ENSMUST00000136414.1	-2,659943448	0	chr5	24394407	24394618

Mir125b-2	ENSMUST00000083538.1	-2,659912435	2341	chr16	77643750	77643931
Slc12a7	ENSMUST00000223454.1	-2,658964536	735	chr13	73803636	73803873
Zfp365	ENSMUST00000138543.1	-2,653220607	2225	chr10	67898921	67899456
Runx1	ENSMUST00000189679.1	-2,651926423	34943	chr16	92780283	92780484
Gm23523	ENSMUST00000180190.1	-2,646564126	9813	chr1	1,71E+08	1,71E+08
Rftn2	ENSMUST00000132055.7	-2,639846574	2307	chr1	55174057	55174248
Gm20412	ENSMUST00000173249.1	-2,637321169	4290	chr2	1,58E+08	1,58E+08
Gm20412	ENSMUST00000173249.1	-2,637321169	4290	chr2	1,58E+08	1,58E+08
Tns3	ENSMUST00000134823.1	-2,63728358	36774	chr11	8582469	8582781
Gsg1	ENSMUST00000111910.3	-2,630784186	12724	chr6	1,35E+08	1,35E+08
Midn	ENSMUST00000151202.1	-2,629747582	0	chr10	80149037	80149412
Gm10773	ENSMUST00000218071.1	-2,625429057	42308	chr10	83070071	83070262
Cacna1a	ENSMUST00000129620.1	-2,621231853	44911	chr8	84501908	84502182
Trib1	ENSMUST00000067543.7	-2,620615603	0	chr15	59647674	59648553
Sec31a	ENSMUST00000094578.10	-2,619002059	25431	chr5	1E+08	1E+08
Zcwpw1	ENSMUST00000035852.13	-2,606674282	153	chr5	1,38E+08	1,38E+08
Dnm1	ENSMUST00000139238.1	-2,606204453	2792	chr2	32342734	32343126
Aldh2	ENSMUST00000152945.2	-2,598949446	7599	chr5	1,22E+08	1,22E+08
Cpt1a	ENSMUST00000237562.1	-2,594954204	197	chr19	3333924	3334409
Gm11366	ENSMUST00000119279.1	-2,594917629	13236	chr13	30194954	30195171
Tmem184b	ENSMUST00000226929.1	-2,593755607	83	chr15	79368823	79369005
Inpp5f	ENSMUST00000130789.1	-2,587856293	4373	chr7	1,29E+08	1,29E+08
Mirt1	ENSMUST00000236257.1	-2,587832582	551	chr19	53450894	53451089
Phf2os1	ENSMUST00000123212.1	-2,587226697	28691	chr13	48842058	48842420
Rdh10	ENSMUST00000027053.7	-2,583722985	3601	chr1	16109377	16109557
Aknad1	ENSMUST00000147916.1	-2,574200276	7017	chr3	1,09E+08	1,09E+08
Angptl6	ENSMUST00000043726.7	-2,572501888	4310	chr9	20878120	20878441
5031415H12Rik	ENSMUST00000181546.1	-2,572167228	8387	chr17	70753718	70754025
5031415H12Rik	ENSMUST00000181546.1	-2,572167228	8387	chr17	70753718	70754025
Efcab1	ENSMUST00000229121.1	-2,572030017	32904	chr16	14939571	14940018
Efcab1	ENSMUST00000229121.1	-2,572030017	32904	chr16	14939571	14940018
Gm25828	ENSMUST00000157087.1	-2,569592508	22632	chrX	1,02E+08	1,02E+08
Gm11476	ENSMUST00000127006.1	-2,568181556	16900	chr2	1,68E+08	1,68E+08
C1qtnf1	ENSMUST00000017590.8	-2,56691017	1903	chr11	1,18E+08	1,18E+08
Rcan1	ENSMUST00000232457.1	-2,566166027	24452	chr16	92423128	92423498
Gm37352	ENSMUST00000193908.1	-2,564036746	2213	chr8	84197433	84198655
Gm9530	ENSMUST00000181160.2	-2,563961653	11308	chr1	1,55E+08	1,55E+08
Mir344i	ENSMUST00000175488.2	-2,562692971	0	chr7	62085070	62085318
Armc9	ENSMUST00000123618.7	-2,562574389	3249	chr1	86151273	86151530
Gm24371	ENSMUST00000122662.1	-2,56203276	4308	chr4	32519097	32519304
Gm26569	ENSMUST00000232356.1	-2,559019665	0	chr16	29944183	29944407
Sox5	ENSMUST00000129050.7	-2,555737427	10030	chr6	1,44E+08	1,44E+08
Gm22053	ENSMUST00000083394.1	-2,553518048	3036	chr14	10481712	10482007
Ltbp3	ENSMUST00000237280.1	-2,549934529	163	chr19	5743504	5743739
Rras	ENSMUST00000044111.9	-2,549636421	1484	chr7	45016158	45016476

Med24	ENSMUST00000144720.1	-2,547999164	0	chr11	98709376	98709727
Ergic1	ENSMUST00000237902.1	-2,542840746	258	chr17	26602484	26602703
Ttll5	ENSMUST00000220906.1	-2,539079454	1650	chr12	85959719	85960350
Gpr176	ENSMUST00000039160.2	-2,535789082	10685	chr2	1,18E+08	1,18E+08
Gm17546	ENSMUST00000168960.2	-2,534300616	99	chr15	95824153	95824358
Nampt	ENSMUST00000020886.8	-2,520168333	22	chr12	32820036	32820312
Nat9	ENSMUST00000103041.7	-2,510868267	9643	chr11	1,15E+08	1,15E+08
Gm47198	ENSMUST00000214736.1	-2,50508562	36757	chr9	47734062	47734383
Gm47134	ENSMUST00000216699.1	-2,502131407	4520	chr9	1,23E+08	1,23E+08
Cdt1	ENSMUST00000006760.2	-2,49529827	18779	chr8	1,23E+08	1,23E+08
Cdt1	ENSMUST00000006760.2	-2,49529827	18779	chr8	1,23E+08	1,23E+08
Card19	ENSMUST00000223159.1	-2,494443195	5807	chr13	49213647	49213842
Ctdspl	ENSMUST00000174132.1	-2,484955072	4288	chr9	1,19E+08	1,19E+08
Gm37607	ENSMUST00000194925.1	-2,481791537	23822	chr1	58359254	58359689
Gm12343	ENSMUST00000138817.1	-2,472755214	2880	chr11	77005818	77006033
Arhgap23	ENSMUST00000152933.1	-2,471980169	94	chr11	97435987	97436190
Tmem267	ENSMUST00000178948.1	-2,468786182	7166	chr13	1,2E+08	1,2E+08
Osbp	ENSMUST00000025590.10	-2,467563288	4027	chr19	11969970	11970185
Cubn	ENSMUST00000195447.1	-2,461518547	10881	chr2	13410546	13411147
Otud5	ENSMUST00000152899.1	-2,454407644	0	chrX	7841234	7841626
Atp2b4	ENSMUST00000128692.1	-2,449532583	1692	chr1	1,34E+08	1,34E+08
Grhl1	ENSMUST00000020985.8	-2,446501621	12612	chr12	24593363	24593561
Spaar	ENSMUST00000131248.1	-2,433738309	1297	chr4	43728490	43728736
Gm13594	ENSMUST00000137582.1	-2,420567599	11034	chr2	65227468	65227721
Egr1	ENSMUST00000165033.1	-2,41526406	102	chr18	34860569	34861104
Usp36	ENSMUST00000106296.8	-2,406132794	10787	chr11	1,18E+08	1,18E+08
Ick	ENSMUST00000142402.1	-2,400942272	7996	chr9	78147127	78147326
Lmna	ENSMUST00000149068.1	-2,397312466	658	chr3	88503413	88503962
Osbpl8	ENSMUST00000217693.1	-2,384624869	1012	chr10	1,11E+08	1,11E+08
Pmp22	ENSMUST00000108702.7	-2,383428762	749	chr11	63129733	63129945
Gm24436	ENSMUST00000157889.1	-2,37561566	6821	chr5	31862708	31863224
Pgbd5	ENSMUST00000140126.1	-2,373528572	3615	chr8	1,24E+08	1,24E+08
Lncpint	ENSMUST00000230874.1	-2,36828333	12317	chr6	31126649	31126855
Gsta2	ENSMUST00000140498.1	-2,36631918	14513	chr9	78348362	78348676
Aldh3a1	ENSMUST00000108716.7	-2,364116255	250	chr11	61208174	61208370
Ksr1	ENSMUST00000129463.1	-2,363496102	26260	chr11	79071445	79071740
Efna5	ENSMUST00000078839.4	-2,359193121	217	chr17	62607538	62607766
Hmga1b	ENSMUST00000105046.3	-2,35032117	0	chr11	1,21E+08	1,21E+08
Hk1	ENSMUST00000142527.1	-2,342558488	3210	chr10	62300170	62300611
S100a3	ENSMUST00000200290.4	-2,337960652	245	chr3	90587199	90587397
Irak2	ENSMUST00000203381.1	-2,335682731	0	chr6	1,14E+08	1,14E+08
Irak2	ENSMUST00000203381.1	-2,335682731	0	chr6	1,14E+08	1,14E+08
Knstrn	ENSMUST00000148877.1	-2,334703902	7998	chr2	1,19E+08	1,19E+08
Gm17501	ENSMUST00000197148.1	-2,314073679	5094	chr3	1,46E+08	1,46E+08
Dclk2	ENSMUST00000193769.1	-2,305484913	4319	chr3	86793667	86793908
Rnu3b4	ENSMUST00000199460.1	-2,304442168	230	chr11	87462518	87463432

Serf2	ENSMUST00000139253.7	-2,298541748	0	chr2	1,21E+08	1,21E+08
Serf2	ENSMUST00000139253.7	-2,298541748	0	chr2	1,21E+08	1,21E+08
Gm10804	ENSMUST00000167654.1	-2,292580804	5447	chr2	93462743	93462991
Gm10804	ENSMUST00000167654.1	-2,292580804	5447	chr2	93462743	93462991
St3gal1	ENSMUST00000230150.1	-2,286177902	32349	chr15	67146589	67146832
Gm15775	ENSMUST00000159796.1	-2,281981651	7469	chr8	1,24E+08	1,24E+08
Gm15775	ENSMUST00000159796.1	-2,281981651	7469	chr8	1,24E+08	1,24E+08
Ppcdc	ENSMUST00000215299.1	-2,279311956	848	chr9	57421036	57421266
Iqgap1	ENSMUST00000205540.1	-2,276709683	6711	chr7	80789748	80790003
Gm42909	ENSMUST00000196982.1	-2,269921471	12007	chr5	12542415	12542624
Ap2a2	ENSMUST00000201261.1	-2,260169105	529	chr7	1,42E+08	1,42E+08
9330111N05Rik	ENSMUST00000181043.7	-2,258536332	247	chr13	80963137	80963474
9330111N05Rik	ENSMUST00000181043.7	-2,258536332	247	chr13	80963137	80963474
Arhgef18	ENSMUST00000238786.1	-2,239995704	8922	chr8	3417429	3417777
Ptges	ENSMUST00000126588.1	-2,238305884	7084	chr2	30899574	30899844
Dusp8	ENSMUST00000143661.1	-2,237334272	648	chr7	1,42E+08	1,42E+08
Ccdc178	ENSMUST00000115837.2	-2,236111805	81295	chr18	21892595	21892790
5430435K18Rik	ENSMUST00000202597.1	-2,222018838	1410	chr5	1,51E+08	1,51E+08
5430435K18Rik	ENSMUST00000202597.1	-2,222018838	1410	chr5	1,51E+08	1,51E+08
Gm45904	ENSMUST00000213082.1	-2,218896182	55649	chr8	1,13E+08	1,13E+08
Ccdc30	ENSMUST00000135997.2	-2,217794903	1071	chr4	1,19E+08	1,19E+08
Ebf1	ENSMUST00000140822.1	-2,212329808	32001	chr11	44944141	44944395
Uqcr11	ENSMUST00000020372.5	-2,209429102	3250	chr10	80398911	80399746
Il6	ENSMUST00000195978.4	-2,208970377	0	chr5	30012822	30013247
Pdgfc	ENSMUST00000143721.1	-2,207430429	64923	chr3	81102349	81102676
Gm24061	ENSMUST00000157489.1	-2,182980897	14587	chrX	1,01E+08	1,01E+08
Smim7	ENSMUST00000163745.1	-2,178518238	237	chr8	72570827	72571017
Vav3	ENSMUST00000137871.1	-2,147327128	27681	chr3	1,09E+08	1,09E+08
Adam10	ENSMUST00000145377.1	-2,139262806	42735	chr9	70733109	70733317
Hr	ENSMUST00000022691.13	-2,135910245	0	chr14	70553913	70554163
Ptprt	ENSMUST00000109441.1	-2,127213522	66183	chr2	1,62E+08	1,62E+08
5033430I15Rik	ENSMUST00000189843.1	-2,115047578	0	chr13	45965130	45965382
Gpa33	ENSMUST00000166860.1	-2,111204021	7425	chr1	1,66E+08	1,66E+08
Itgb5	ENSMUST00000148462.1	-2,108723115	0	chr16	33891904	33892143
Gm50351	ENSMUST00000235424.1	-2,091551282	100	chr18	82717608	82717867
Gm2895	ENSMUST00000171591.1	-2,083943943	293	chr15	66968567	66969029
Gm47076	ENSMUST00000221366.1	-2,060224576	6955	chr13	34112550	34112745
Gm23479	ENSMUST00000158876.1	-2,053424287	7026	chr7	98928508	98928867
1700052H01Rik	ENSMUST00000196142.1	-2,040745505	9465	chr3	41377093	41377326
Nprl3	ENSMUST00000129010.1	-2,01096638	1027	chr11	32251192	32251420
Tns4	ENSMUST00000123303.1	-1,997447935	3244	chr11	99081757	99082226
Ermard	ENSMUST00000186130.1	-1,995874604	378	chr17	15065995	15066439
Iba57	ENSMUST00000137433.1	-1,975881075	2049	chr11	59163406	59163856

Frmd8	ENSMUST00000025728.12	-1,965745042	19	chr19	5849188	5849682
Plcxd2	ENSMUST00000130481.1	-1,96226002	9437	chr16	45968702	45968982
Plcxd2	ENSMUST00000130481.1	-1,96226002	9437	chr16	45968702	45968982
Nsmce2	ENSMUST00000079703.10	-1,948114123	37832	chr15	59412121	59412485
Apol6	ENSMUST00000142405.7	-1,920165778	7218	chr15	77037280	77037510
C330002G04Rik	ENSMUST00000236056.1	-1,893774251	61065	chr19	22916954	22917164
Rapgef4	ENSMUST00000102698.9	-1,865929449	585	chr2	71981874	71982069
Disp1	ENSMUST00000192915.1	-1,82146789	17542	chr1	1,83E+08	1,83E+08
St3gal6	ENSMUST00000232391.1	-1,80356889	9482	chr16	58512351	58512580
Fbln2	ENSMUST00000137029.1	-1,798959514	3801	chr6	91269682	91270182
Lncppara	ENSMUST00000230913.1	-1,796870848	15513	chr15	85669131	85669313
Mir1954	ENSMUST00000175595.1	-1,778392821	1579	chr2	32653910	32654097
Oma1	ENSMUST00000133178.1	-1,758668541	18303	chr4	1,03E+08	1,03E+08
Dusp1	ENSMUST00000146077.1	-1,739949393	2154	chr17	26508672	26508856
Gnat3	ENSMUST00000030561.8	-1,727275886	37774	chr5	18000325	18000562
Abhd8	ENSMUST00000212321.1	-1,718504613	0	chr8	71457163	71457724
Tle3	ENSMUST00000159386.7	-1,690840678	0	chr9	61372239	61372543
Pde4d	ENSMUST00000135275.7	-1,630876284	26265	chr13	1,1E+08	1,1E+08
Alkbh5	ENSMUST00000044250.3	-1,602926179	43	chr11	60537718	60537934
Gm47311	ENSMUST00000198228.1	-1,578301341	554	chr3	96240424	96240693
Gm43242	ENSMUST00000199507.1	-1,562389599	3314	chr3	1,02E+08	1,02E+08
Gm17484	ENSMUST00000167899.2	-1,561311817	0	chr8	25754458	25754733
Gm17484	ENSMUST00000167899.2	-1,561311817	0	chr8	25754458	25754733
Arid3a	ENSMUST00000131118.1	-1,559507536	6676	chr10	79937102	79937406
Ccl9	ENSMUST00000136131.1	-1,558045222	2954	chr11	83578274	83578564
Nr3c2	ENSMUST00000126697.1	-1,546302704	5177	chr8	77133192	77133445
Hist1h1d	ENSMUST00000045301.8	-1,531274673	1908	chr13	23555172	23555452
Hist1h2bn	ENSMUST00000091709.2	-1,476280752	0	chr13	21753948	21754243
Gm43184	ENSMUST00000197247.1	-1,442076056	107	chr5	43948229	43948622
Gm43184	ENSMUST00000197247.1	-1,442076056	107	chr5	43948229	43948622
Gm43811	ENSMUST00000202862.1	-1,404820531	9154	chr5	31935853	31936047
Per1	ENSMUST00000021271.13	-1,385569013	1359	chr11	69097345	69097588
Hist1h2bc	ENSMUST00000018246.5	-1,347803971	0	chr13	23684027	23684215
Hes1	ENSMUST00000160592.2	-1,341610421	0	chr16	30063648	30064627
Frmd8os	ENSMUST00000135347.1	-1,317314441	0	chr19	5848240	5848727
Gm43274	ENSMUST00000202545.1	-1,294428558	3130	chr5	1,19E+08	1,19E+08
Gm43274	ENSMUST00000202545.1	-1,294428558	3130	chr5	1,19E+08	1,19E+08
Hist1h2bk	ENSMUST00000110455.3	-1,147773255	89	chr13	22035444	22035780
Hist2h2aa1	ENSMUST00000078756.6	-0,887663836	89	chr3	96245153	96245407
Hist1h2ad	ENSMUST00000090776.6	-0,721396172	0	chr13	23574228	23574544
Hist1h2bb	ENSMUST00000099703.4	-0,679585429	37	chr13	23746365	23746696
Gm10721	ENSMUST00000143083.2	-0,621097781	437	chr9	3012502	3012702

Supplementary Table 4. Clinicial and pathology data for PDAC patients

Internal identifier	pT	Size	pN	pM	Grade	Age	Gender	PanIn hi	Tu central
H 20944/13	3	6.8	2		3	70	male	0	0
H 23510/06	1c	1.5	1		2	56	female	0	0
SS 70093/99	2	4	2		2	62	male	0	0
H 13936/01	3	4.8	2		2	60	female	0	0
SS 72365/01	2	2.9	1		3	76	female	0	0
SS 70682/02	3	4.8	1		2	70	male	0	0
SS 71299/02	2	2.2	0		1	82	female	0	0
H 18448/03	3	4.5	2		2	73	male	0	0
SS 72272/03	3	4.5	1		3	69	female	0	0
H 15364/05	2	4	0		2	82	male	0	0
SS 72116/05	3	6	1		2	78	female	0	0
H 17445/97	2	3	1		2	50	female	0	0
SS 70964/97	2	2.5	1		2	64	male	0	0
SS 71594/97	2	3.5	0		2	70	male	0	0
H 26137/98	2	2.5	2		2	37	female	0	0
SS 72296/98	3	5	1		2	64	female	0	0
H 9850/09	3	6	2		3	76		1	0
H 22982/06			1	1	2	59	female	1	0
H 15760/07	2	2.9	2		3	76	male	1	0
H 1917/11	3	9	0	0	2	74	male	1	0
H 3984/12	2	3.5	2	0	2	70	male	1	0
SS 72813/01	2	3.5	0		2	66	female	1	0
SS 73223/01	2	2.5	0		2	70	male	1	0
H 27973/02	2	3.2	0		2	68	male	1	0
H 18721/03	1c	1.8	1		1	62	female	1	0
SS 73459/04	1c	2	0		2	63	female	1	0
H 9822/05	1c	1.2	0		2	79	male	1	0
SS 73295/05	3	8.5	2		3	61	male	1	0
H 72296/99	2	3	0		3	73	male	1	0
H 24983/99	2	4	0		3.00	70	male	1	0
H 14330/00	2	2.2	2		2	57	male	1	0
H 14455/00	2	3.5	2		2	67	female	1	0
H 21847/00								1	0
SS 73246/00	2	2.5	1		1	61	male	1	0
SS 73275/00	2	3	2		3	52	female	1	0
H 38817/08	1c	2	0		2	71	male	2	0
H 23349/06	2	2.5	0		2	70	female	2	0
H 10516/06	3	8.5	2	1	4	39	female	2	0
H 12850/10	1c	1.6	2	0	1	67	male	2	0
H 16563/11	2	3	1		2	74	female	2	0
H 4739/12	2	2.4	2	0	3	65	female	2	0
SS 72363/99	3	7	2		3	65	male	2	0
SS 73517/99	3	4.5	2		3	65	male	2	0
SS 71829/01	3	8	0		2	60	male	2	0
SS 72734/01	2	2.1	0		3	72	female	2	0

SS 72839/01	2	3.5	2		2	76	female	2	0
SS 73427/01	3	4.5	1		3	70	female	2	0
SS 70073/98	2	3.2	1		3	66	female	2	0
SS 72249/00	2	3.8	1		2	70	male	2	0
H 13410/12	3	5	0	0	3	52	male	0	1
H 14540/01			0		3	69	male	0	1
H 26109/05	2	2.5	0		2	66	female	0	1
SS 72908/05	2	2.5	0		2	59	female	0	1
SS 70576/98	2	3	0		3	67	female	0	1
SS 70379/00	2	2.5	0		1	69	female	0	1
H 39657/13	2	2.8	1	0	2	76	female	1	1
H 15374/13	2	4	1	1	2	76	male	1	1
H 4175/09	2	2.8	0		2	73	male	1	1
H 8869/10	2	3.8	0	0	3	67	female	1	1
SS 70862/99	2	2.5	1		2	71	female	1	1
H 3518/01			1		3	59	female	1	1
H 15896/02	2	3.2	0		2	66	male	1	1
H 12022/03	3	4.5	0		2	68	male	1	1
SS 70533/04	1c	2	0		2	63	female	1	1
SS 70995/05	2	3.5	0		2	75	female	1	1
SS 71066/05	2	3	1		2	74	male	1	1
SS 72500/97	2	3.7	1		2	46	female	1	1
H 6963/00			0			61	female	1	1
H 14495/09	2	2.4	2		2	68	male	2	1
H 23077/09	3	4.7	0		3.00	82	female	2	1
SS 71459/08	3	5.3	1		3	76	male	2	1
H 22193/06	3	5	2		3	65	male	2	1
H 6509/12	3	7.5	2	1	2	49	female	2	1
H 18485/12	2	4	2	0	3.00	80	female	2	1
H 30759/04	3	4.5	2		3	63	female	2	1
SS 73564/05	2	3.1	0		3	72	male	2	1
H 15377/09	2	2.2	0		3	79	male	3	1
H 29671/09	2	4	1		2	75	male	3	1
H 25773/12	2	3.8	2		2	74	female	1	2
SS 73033/13								2	2
SS 70004/02	1c	1.5	0		2	62	female	2	2
SS 73813/05	2	3	2		3	70	female	2	2
H 23794/09	2	2.7	1		3	72	female	3	2
SS 73291/98	2	3	2		2	56	female	3	3

Supplementary Table 5. List of DNA oligos

qPCR primers	Sequence (5' to 3')
mKDM6A_F	AAGGCTGTTCGCTGCTACG
mKDM6A_R	GGATCGACATAAAGCACCTCC
mTSC1_F	ACTCTCCCTTCTACCGAGACA
mTSC1_R	GAGGCTGCCGAATGAGTCTTC
mTSC2_F	GAGCTGATTAACTCGGTGGTC
mTSC2_R	GGCCAGGTCCCTTTCTTCC
mDEPTOR_F	ATAGACGGCACCATCTCAAAAC
mDEPTOR_R	GTCGGCTAATTTCTGCATGAGT
mTubulin_F	TCACTGTGCCTGAACTTACC
mTubulin_R	GGAACATAGCCGTAAACTGC

Primers for T7 assay	Sequence (5' to 3')
T7_mKdm6a.1_F	CAACAACTTTGTGCTGGTGCC
T7_mKdm6a.1_R	GTTTGCCTTACCTTGCCCAGCAG
T7_mKdm6a.2_F	CTGACTCCTCACTAATGCAG
T7_mKdm6a.2_R	CATGCCCTGCTGGTTGAAGTGG

Oligos for shRNA cloning	Sequence (5' to 3')
mouse_shKdm6a.1	TGCTGTTGACAGTGAGCGATGCTACGAATCTCTAATCTTATAGTGAAG CCACAGATGTATAAGATTAGAGATTCGTAGCAGTGCCTACTGCCTCGGA
mouse_shKdm6a.2	TGCTGTTGACAGTGAGCGAACAGACTATGAGTCTAGTTTATAGTGAAG CCACAGATGTATAAACTAGACTCATAGTCTGTGTGCCTACTGCCTCGGA
mouse_shRenilla	TGCTGTTGACAGTGAGCGCAGGAATTATAATGCTTATCTATAGTGAAG CCACAGATGTATAGATAAGCATTATAATTCCTATGCCTACTGCCTCGGA

Oligos for guides	Sequence (5' to 3')
mKdm6a.1 guide	TCATCACCGAAAGCGGCGG
mKdm6a.2 guide	CCCAGCTTTTGTCGAGCCA
mTp53 guide	ACCCTGTCACCGAGACCCC
mDeptor.1 guide	TGCAGAAATTAGCCGACCG
mDeptor.2 guide	CAGAATGAACTTCCGTCGG
mS6K1 guide	TTTATGCCTTTCAGACCGG
mMtor guide	GATACGAACTAGCTCGTTG

Supplementary Table 6. List of antibodies

Antibodies for Western Blotting	Source	Identifier
Kdm6a (UTX)	Cell Signaling Technology	33510
Deptor	Novus Biological	NBP1-49674
Tsc1	Cell Signaling Technology	6935
pS6RP S235/6	Cell Signaling Technology	4858
pS6RP S240/4	Cell Signaling Technology	5364
S6RP	Cell Signaling Technology	2217
Actin	Sigma-Aldrich	PA5-22325
Vinculin	Sigma-Aldrich	V9131

Antibodies for CUT&RUN	Source	Identifier
Kdm6a (UTX)	Cell Signaling Technology	33510
H3K27me3	Cell Signaling Technology	9733
H3K27ac	Abcam	ab4729
H3K4me1	Abcam	ab8895
H3K4me3	Abcam	ab8580
IgG	Abcam	ab37415

Antibodies for IHC	Source	Identifier
Kdm6a (UTX)	Cell Signaling Technology	33510
Deptor(human)	Cell Signaling Technology	11816
pS6RP S235/6 (human)	Cell Signaling Technology	2211
CK19 (human)		
Deptor (mouse)	Novus biological	NBP1-49674
pS6RP S235/6 (mouse)	Cell Signaling Technology	4857
pS6RP S240/4 (mouse)	Cell Signaling Technology	5364
cleaved Caspase 3 (mouse)	Cell Signaling Technology	9661
GFP (mouse)	Cell Signaling Technology	2956
